# An experimental comparison of composite and grab sampling of stream water for metagenetic analysis of environmental DNA

**DOI:** 10.7717/peerj.5871

**Published:** 2018-12-05

**Authors:** Robert S. Cornman, James E. McKenna, Jennifer Fike, Sara J. Oyler-McCance, Robin Johnson

**Affiliations:** 1 Fort Collins Science Center, U.S. Geological Survey, Fort Collins, CO, USA; 2 Great Lakes Science Center, Tunison Aquatic Laboratory, U.S. Geological Survey, Cortland, NY, USA; 3 Integrated Statistics, Woods Hole, MA, USA

**Keywords:** Metagenetics, Environmental DNA, Sampling methods, Biodiversity

## Abstract

Use of environmental DNA (eDNA) to assess distributions of aquatic and semi-aquatic macroorganisms is promising, but sampling schemes may need to be tailored to specific objectives. Given the potentially high variance in aquatic eDNA among replicate grab samples, compositing smaller water volumes collected over a period of time may be more effective for some applications. In this study, we compared eDNA profiles from composite water samples aggregated over three hours with grab water samples. Both sampling patterns were performed with identical autosamplers paired at two different sites in a headwater stream environment, augmented with exogenous fish eDNA from an upstream rearing facility. Samples were filtered through 0.8 μm cellulose nitrate filters and DNA was extracted with a cetyl trimethylammonium bromide procedure. Eukaryotic and bacterial community profiles were derived by amplicon sequencing of 12S ribosomal, 16S ribosomal, and cytochrome oxidase I loci. Operational taxa were assigned to genus with a lowest common ancestor approach for eukaryotes and to family with the RDP Classifier software for prokaryotes. Eukaryotic community profiles were more consistent with composite sampling than grab sampling. Downstream, rarefaction curves suggested faster taxon accumulation for composite samples, and estimated richness was higher for composite samples as a set than for grab samples. Upstream, composite sampling produced lower estimated richness than grab samples, but with overlapping standard errors. Furthermore, a bimodal pattern of richness as a function of sequence counts suggested the impact of clumped particles on upstream samples. Bacterial profiles were insensitive to sample method, consistent with the more even dispersion expected for bacteria compared with eukaryotic eDNA. Overall, samples composited over 3 h performed equal to or better than triplicate grab sampling for quantitative community metrics, despite the higher total sequencing effort provided to grab replicates. On the other hand, taxon-specific detection rates did not differ appreciably and the two methods gave similar estimates of the ratio of the common fish genera *Salmo* and *Coregonus* at each site. Unexpectedly, *Salmo* eDNA dropped out substantially faster than *Coregonus* eDNA between the two sites regardless of sampling method, suggesting that differential settling affects the estimation of relative abundance. We identified bacterial patterns that were associated with eukaryotic diversity, suggesting potential roles as biomarkers of sample representativeness.

## Introduction

While genetic analysis of environmental microbial diversity has been maturing over the past two decades, a flood of recent literature has documented the persistence of environmental DNA (eDNA) deriving from macroscopic organisms as well. “eDNA” appears to be dominated by cell-sized particles patchily distributed in the environment (Pilliod et al., 2014; Turner et al., 2014; Wilcox et al., 2015). While factors influencing eDNA persistence and movement within aquatic systems, and ultimately the relation between eDNA detection and the distribution of source organisms, remain topics of active research, eDNA has nonetheless been shown to be a useful means to infer occupancy of a target species, once validated (Goldberg et al., 2016).

Assays based on various quantitative PCR (qPCR) strategies can achieve low limits of detection and currently predominate for single-species monitoring. However, there is a burgeoning effort to more broadly characterize the diversity of eDNA sources present, via barcode sequencing (Bohmann et al., 2014). While barcode sequencing will typically be less sensitive for a given target than a qPCR assay, and is often biased in taxonomic recovery (Clarke et al., 2014; Krehenwinkel et al., 2017), it nonetheless provides a rapid and rich assessment of taxonomic diversity within major clades. The utility of eDNA barcodes to approximate biodiversity and ecosystem condition appears promising (Laroche et al., 2017; Stat et al., 2017; O’Donnell et al., 2017; Bista et al., 2017), either as the primary goal of eDNA sampling or in conjunction with other objectives.

While many sampling methods are in use and eDNA-specific technology is being explored, “grab” sampling (i.e., a sample volume taken at a single point in time and space with a pump, dipping device, van dorn sampler, or comparable means) remains common and attractive for its simplicity and versatility. However, grab samples may not representatively capture clumped or temporally variable templates, resulting in greater technical variation among replicates for heterogeneously distributed classes of eDNA and lower precision of metrics such as richness or pairwise multivariate distance. Alternatively, analysis samples can be aggregated from numerous smaller volumes or aliquots (“composite” samples) collected over spatial or temporal intervals. The benefits and limitations of composite sampling have been examined in a number of microbiological applications (Jarvis, 2007; Reicherts & Emerson, 2010) that seek reduced cost or increased representativeness of the data.

Given the frequently variable nature of eDNA detections among replicate grab samples, we hypothesized that composite sampling might detect rarer species more consistently, detect higher taxonomic richness overall, and produce more consistent community profiles among replicates. However, composite sampling by human hands would likely be more expensive, logistically more challenging, and more susceptible to error than an automated process. We therefore evaluated composite and grab sampling using a widely available automated water sampling device (Teledyne ISCO model 6712). Similar automated systems are frequently used to obtain water samples for chemical analyses in industrial and public-health applications (U.S. Geological Survey, 2006, 2010). Some systems conduct analyses in situ, including prototypes for genetic analysis (Yamahara et al., 2015), whereas others store samples for transport to a laboratory. The models deployed here were programmed to collect either triplicate grab samples collected in separate bottles or single sample bottles composited from 12 equal volumes collected at 15-min intervals, as representative sampling schemes for comparison.

We evaluated these sampling schemes in a natural environment, but one supplemented with a strong ex situ eDNA source. Specifically, we deployed pairs of autosamplers at two sites (four autosamplers total) along a small woodland stream of the northeast US, downstream of the effluent of a facility that rears Atlantic Salmon (*Salmo salar*), Cisco (*Coregonus artedi*), and Bloater (*C. hoyi*) at high density. This design allowed us to compare sampling effects for these known, high-abundance species as well as the unknown but presumably more rare and variable eDNA pool shed by resident species. The primary metrics of the comparison were: detection rates of individual taxa, estimated sample richness with rarefaction, quantitative relative abundance of the most common taxa (based on actual proportions), and variance in overall taxonomic composition (based on Morisita’s dissimilarity index).

To identify eDNA sources, we sequenced multiple genetic barcodes targeting different classes of DNA template that we expected to differ qualitatively in the homogeneity of their dispersion: a 12S ribosomal locus targeting vertebrates, a cytochrome oxidase 1 (COI) locus targeting metazoans (particularly invertebrates), and a 16S ribosomal locus targeting the phytoplankton. The 16S primers predominately amplify bacteria (Needham & Fuhrman, 2016), which reproduce by fission and occur at densities on the scale of 10^6^–10^7^ per mL in freshwater (Bird & Kalff, 1984), providing a strong contrast to the more clumped distribution expected of eukaryotic eDNA. Moreover, bacterial profiles are themselves of interest as potential biomarkers of sample condition or representativeness, because changing profiles may indicate unobserved environmental factors that could impact eDNA detection probabilities. For example, microbial profiles could potentially reveal changing biofilms, sediment loads, or water chemistry, which could in turn influence levels, persistence, or detection of eDNA (Barnes et al., 2014; Barnes & Turner, 2016). Additionally, as components of metazoan microbiomes, specific microbial taxa could potentially corroborate or qualify the interpretation of co-occurring metazoan eDNA. Therefore, in addition to contrasting sampling effects between bacterial and eukaryotic groups, we investigated whether samplers at a site recovered similar bacterial profiles—evaluating the assumption that the sampled waters were comparable—and used ordination to examine which bacterial taxa most differentiated sites and samples.

## Materials and Methods

### Study area

**Figure 1:**
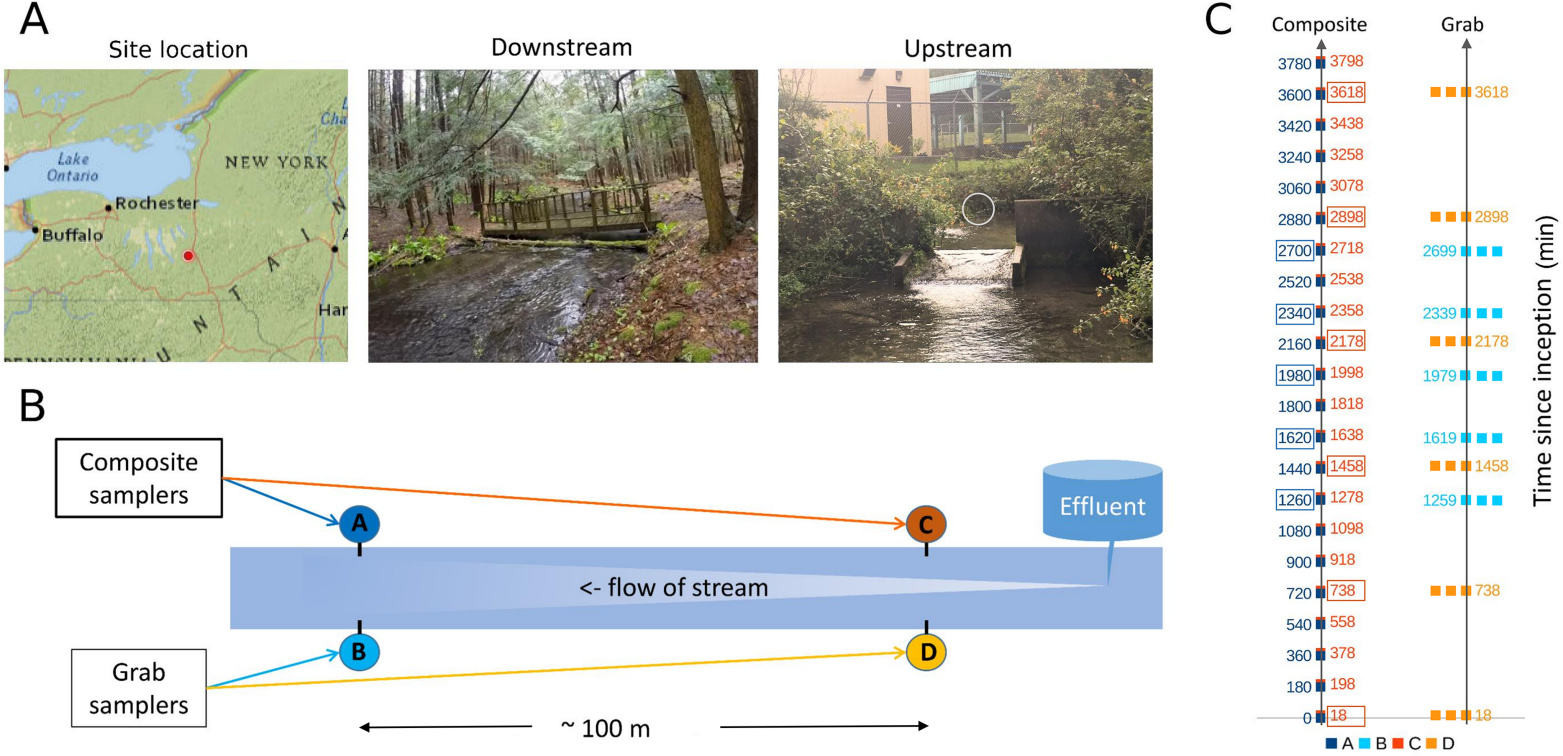
Sampling location and design. (A) Location of sampling area within New York state and images of upstream and downstream sampling sites. The location of the effluent pipe of the fish rearing facility is circled in the upstream image. Photo credit: J. E. McKenna. (B) Schematic of relative positions of paired downstream and upstream autosamplers. For brevity, samplers are referred to by letter codes (A–D) in subsequent figures, and similar color schemes are also used throughout to link figure elements to the sampler from which they derive. Effluent denotes the outflow from the Tunison Aquatic Laboratory fish rearing facility. (C) Tempo of sampling by each autosampler. Each composite sample is initiated at a time in minutes since the start of the experiment, and consists of 12 45-mL volumes composited over 3 h. These are indicated by single points of the appropriate color. Triplicate grab samples (represented by three points) were taken consecutively at the intervals indicated. Composite samples initiated at approximately the same time as a set of triplicate grab samples, and paired with those for some analyses, are indicated by colored boxes.

Sampling occurred at two locations in Tunison Brook (approximately 42.56 N, 76.25 W; Fig. 1A) downstream of the outfall from the US Geological Survey, Tunison Laboratory of Aquatic Science fish culture facility, Cortland, New York, USA. Tunison Brook is a first order headwater stream flowing through hemlock and deciduous forest in the Oswego River watershed, which drains into Lake Ontario (McKenna, 2017). Previous observations of the fish assemblage in Tunison Brook were used to constrain taxonomic assignments of eDNA sequences (see below).

Sites approximately 100 m apart were selected based on accessibility (Fig. 1B), primarily for the purpose of replicating the methodological comparison. Differences in eDNA communities at this scale were of general interest but not germane to the hypotheses tested, excepted were noted in the Results. The upstream site was approximately eight m below the hatchery effluent outfall. At the beginning of the experiment, flow at the outfall was 0.41 m/s and flow at the downstream site was 0.18 m/s. Stream flow appeared stable over the course of the study but no repeated measures were taken.

### Autosampler deployment

Four ISCO model 6712 samplers (Teledyne ISCO, Lincoln, NE, USA) were deployed using 60-foot long, 3/8-inch diameter vinyl intake tubing and sets of 24 one-L bottles. Collection bottles were chilled with an internal ice basin but no preservative or buffering solution was pre-loaded in the bottles for these trials. The intake line of the sampler was evacuated prior to acquiring any new sample volume, such that the new sample is water currently present in the stream and not residual sample in the intake line. However, the samplers do not ensure strict containment, in that residues from the intake tubing, valves, or other components can potentially cross-contaminate samplers. We explicitly assume that this route of cross-contamination contributes negligible numbers of sequence reads relative to the total eDNA in a sample, and occurs randomly with respect to sample type.

At each of the two locations, one autosampler was programmed to take three 500-mL samples consecutively, as a representative grab sample set. Each grab sample set was to be taken every 12 h for the course of the experiment. This program was executed as intended on the upstream grab sampler, but due to an equipment error at the beginning of the run, the downstream grab sampler was reprogrammed on the fly to take the grab replicate set every 6 h. A second autosampler at each site was programmed to composite 45-mL volumes every 15 min, proceeding to the next sample bottle after 3 h (total volume of 540 mL). Grab sample replicates can be considered paired with a “time-matched” composite sample that was initiated at approximately the same time (± 1 min), whereas other composite samples were initiated between two different sets of grab samples and are not paired with a set of replicate grab samples in this sense (Fig. 1C). The timing of samples taken by each method at each site is illustrated in Fig. 1C; composite samples that are matched with grab replicates in paired analyses are indicated by boxes.

### Sample processing

Prior to the experiment, sample collection bottles were soaked in 10% sodium hypochlorite and repeatedly rinsed in water. After the experiment, approximately 300 mL from each sample bottle was filtered through a 0.8-μm cellulose nitrate filters of 25-mm diameter (Whatman item number 7188-002). Filtration occurred under 30 psi negative pressure (Thomas Industries model 907CA18-554 air compressor) using standard filtration flasks and fittings. The filtering apparatus was soaked in 10% sodium hypochlorite between uses, washed twice with tap water and once with deionized water, then air-dried. Filters were handled with ethanol-sterilized forceps. Processed filters were folded into a 15-mL Falcon tube (Corning) and stored in a freezer rated at −20 °C (operational temperature was measured to be approximately −12 °C). Filters were later transported on ice to the US Geological Survey, Leetown Science Center, Kearneysville, West Virginia, for nucleic acid extraction with the cetyl trimethylammonium bromide protocol of Renshaw et al. (2015). Extraction yield was measured with a Qubit fluorometer using the High-sensitivity DNA kit (ThermoFisher, Waltham, Massachusetts, USA).

### Barcode amplification and sequencing

Three genetic barcodes were investigated that have been developed for major organismal groups representing distinct trophic levels: a cytochrome oxidase barcode targeting metazoans broadly but which is particularly effective for invertebrate clades (primers “MICO1int” and “jgHCO2198” of Leray et al., 2013); a mitochondrial ribosome barcode targeting vertebrates (the “12S-v5” primer set of Riaz et al., 2011); and a 16S ribosomal barcode targeting phytoplankton, including plastids of eukaryotic phytoplankton (Needham & Fuhrman, 2016).

An initial amplification was performed for each barcode on each sample with unmodified primers. Amplifications were performed in 25 μL volumes with two ng of DNA extract, 0.2 mM of each dNTP, 0.5 μM forward primer, 0.5 μM reverse primer, 1.25 U GoTaq Flexi DNA polymerase (Promega, Madison, WI, USA), 1.5 mM MgCl_2_, and 1X GoTaq Flexi Buffer (Promega, Madison, WI, USA). Amplification conditions were as follows: 95 °C for 2 min, then 95 °C for 45 s, 50° for 45 s, 72 °C for 1 min 30 s for either 20 cycles (16S primers) or 25 cycles (12S and COI primers), followed by 72 °C for 2 min. A second PCR was performed to add sequencing adaptors for the Illumina MiSeq platform, using three μL of the pre-amplification product as template. This was done in 25 μL reactions with 0.5 μM modified forward primer, 0.5 μM modified reverse primer, 12.5 μL KAPA2G Fast HotStart ReadyMix (KAPA Biosystems, Wilmington, MA, USA), and 0.25 μL BSA (New England Biolabs, Ipswich, MA, USA). Amplification conditions for this PCR were: 95 °C for 2 min, then 95 °C for 45 s, 50° for 45 s, 72 °C for 1 min 30 s for 25 cycles, followed by 72 °C for 2 min. To evaluate amplification strength, five μL of reaction product was visualized in 2% agarose gels stained with ethidium bromide.

Equal volumes of all three amplicons were combined per sample, cleaned with an UltraClean HTP 96-well PCR clean-up kit (MoBio, Carlsbad, CA, USA) and eluted in 30 μL water. Each sample was barcoded with dual Nextera XT indexes. The 50 μL indexing reactions used five μL of cleaned adaptor PCR product, five μL of each index, 25 μL of 2xKAPA HiFi HotStart ReadyMix, and 10 μL water. Amplification conditions were 95 °C for 3 min, then eight cycles of 95 °C for 30 s, 55 °C for 30 s, and 72 °C for 30 s, with a final extension at 72 °C for 5 min. The indexed PCR product was again processed with the UltraClean kit and eluted in 50 μL water. Samples were quantified using a Qubit High Sensitivity DNA Assay (Life Technologies, Carlsbad, CA, USA), pooled proportionately to achieve equal concentrations, and diluted to a concentration of four nM. The final library prep was denatured and diluted to 12 pM, spiked with 30% phiX control sequence and sequenced on an Illumina MiSeq with a 600-cycle Version 3 chip (Illumina) to produce 300-bp paired reads. Raw, demultiplexed sequence reads are available through the National Center for Biotechnology Information (NCBI) BioProject PRJNA431582. In accordance with U.S. Geological Survey policy, these NCBI accessions as well as the operational taxonomic units (OTUs) identified from the data have been deposited in an approved federal repository (Cornman et al., 2018).

### Read processing and selection of operational taxonomic units

Because 12S amplicons (∼135–150 bp) were shorter than a single read length (300 bp), they were processed differently than the 16S and COI loci. To identify the latter, read pairs were imported into CLC Genomics v. 9.5 (Qiagen, Valencia, CA, USA) and trimmed of low-quality base calls and exogenous sequences. The maximum Phred-scaled error probability of base calls was set to 10, and at most two ambiguous characters were allowed per read. Multiple explicit variants were used as search motifs to trim degenerate primer sequence. Trimmed read pairs were then merged in CLC Genomics using the default scoring scheme (a minimum score of 25, with each overlapped position contributing +2 for a match or −3 for a mismatch). Merged reads were exported in fasta format and clustered with cd-hit-est (Fu et al., 2012) at 97% identity and a reciprocal overlap of 90%. The size of each cluster was assessed by mapping merged reads to clusters with bowtie2, using the “fast” and “end-to-end” parameter switches. The 19,781 OTUs with 10 or more sequence counts in the entire sequence run were retained for further analysis. While it is common to remove singleton OTUs, for the present study we preferred a more conservative approach (i.e., increasing the minimum cluster size from two to 10) because of the recognized hazards of sample cross-talk (MacConaill et al., 2018) and PCR artifacts (Haas et al., 2011) in inflating diversity. Given the study aims, inflation of diversity seemed more detrimental to us than any potential biases arising from this censoring that would accrue equally to each sampling method.

After purging OTUs with ambiguous characters in the overlap region (which indicate discordant or undetermined base calls in the overlapped region of each pair), 12,693 OTUs remained. A chimera-removal step was performed with vsearch (Rognes et al., 2016) using the “uchime_denovo” algorithm (Edgar et al., 2011), but no chimeras were flagged by this method. OTUs were then assigned to either the 16S or COI locus using BLASTN alignments (BLAST+ v. 2.3.0) to locus-specific databases. These databases were the OTUs themselves that had the terms “16S ribo” or “cytochrome” in the top three matches to NCBI’s nt database (6,788 and 843 OTUs, respectively). This binning method was used because it is difficult to specify NCBI database searches that retrieve only sequences of the targeted loci, properly bounded. Note that this binning has no bearing on the eventual taxonomic assignment of an OTU, it merely groups OTUs based on NCBI annotations so that locus-specific filters can be imposed. OTUs that failed to match a locus-specific database at a minimum bit score of 100 were presumed to be low-complexity or off-target sequence. Finally, the length distributions of OTUs in each bin were examined for outliers. The 1,461 COI-binned OTUs ranged from 312 to 379 bases, none of which were excluded as outliers. The 10,770 16S OTUs ranged from 327 to 422 bases; one of these was excluded after imposing a minimum length of 350 bases.

12S OTUs were identified using only the forward read of read pairs. Candidate 12S reads were considered those that did not map as a valid pair to a COI or 16S cluster (see below). Primers and low-quality bases were trimmed with the bbduk package (Joint Genome Institute, 2018) with a minimum kmer match to primers of 11 and a minimum Phred-scaled base quality of 10. Clustering was performed with vsearch (Rognes et al., 2016) using a 98% identity threshold, to accommodate the lower level of differentiation expected at this locus for speciose fish clades (e.g., salmonids and cyprinids). At this step, OTU representative sequences were required to be >75 bases and ≤170 bases in length, and singleton 12S OTUs were discarded. The representative sequences for all 7,355 retained 12S OTUs are provided in Table S1.

### Taxonomic assignment of OTUs

Distinct taxonomic assignment methods were used for each genetic barcode. Taxonomic assessment of 12S and COI OTUs was performed using the lowest common ancestor (LCA) method (Huson et al., 2007) as modified below. Taxonomic assessment of 16S OTUs was performed with the RDP Classifier v. 2.11 (Wang et al., 2007) using the native training for bacteria (version 16). 16S OTUs assigned to the chloroplast clade were considered eukaryotic phytoplankta and re-analyzed using the LCA method. For bacterial 16S, we retained 5,279 OTUs with an RDP classification of 0.80 or higher at the family level. Bacterial taxa with a genus assignment but lacking a family designation were given an operational family designation by appending “incertae sedis” to the genus. As some taxa present in the RDP Classifier scheme were not present in the NCBI taxonomy scheme, these taxa were assigned to synonymous or more inclusive NCBI taxa as indicated in Table S2.

We investigated the RDP Classifier as an alternative to LCA for COI classification, using COI reference sequences from the Barcode of Life Database (BOLD) (downloaded on April 24, 2017) (Ratnasingham & Hebert, 2007) for phyla Chordata, Arthropoda, Mollusca, Annelida, Nematoda, and Rotifera. While we found strong agreement between RDP and LCA assignments for chordates, many high-scoring LCA assignments for invertebrates were of taxa not present in the RDP training sets. We therefore used only the LCA method for eukaryotic taxonomy, as COI accessions available for RDP training did not appear to be sufficiently representative of this environment (LCA does not require training or strictly bounded reference sequences).

The LCA method is a heuristic classification based on the distribution of alignment scores of OTU representative sequences to a reference sequence database. For each OTU, the algorithm identifies the taxon that is root to all matches scoring within a specified percentage of the best score. The BLASTN program is a commonly used alignment tool for LCA and the bit score of high-scoring pairwise alignments (HSPs), a commonly used scoring metric. We used NCBI’s Nucleotide (nt) database (downloaded on December 27, 2016) as the reference database. The NCBI taxonomy scheme used to associate matched accessions to taxa was downloaded on March 17, 2017. For COI and eukaryotic 16S assignments, a 5% LCA threshold was used and only HSPs with bit scores of 250 or greater were retained. While the percentage identity of matches reported by BLASTN is not directly useful for LCA, because it is not scaled to the fraction of the read that actually aligns (“query coverage”), it can be used secondarily as a filter to remove or demote poor-quality assignments. Here we required that for species-level LCA assignments, the mean percentage identity for contributing HSPs be 95% or greater. For genus level assignments, this value was required to be 90% or greater. The final number of OTUs above these thresholds was 149 and 18 for COI and eukaryotic 16S, respectively. Implementation of the LCA method is described in detail in File S1.

Assignment at the 12S locus followed a modified LCA procedure that further differentiated fish taxa from other vertebrates. 12S OTUs were first assigned by LCA analysis of BLASTN alignments to the nt database as described above, but using a minimum bit score of 200 and a 2% LCA threshold. Taxa falling within the Actinopterygii (ray-finned fish) were then re-evaluated after filtering alignments to taxa not present on a list of expected fish taxa (Table S3). This constraint was necessary because the 12S locus lacks resolution to discriminate among all species represented within nt. The list of expected fish taxa included species observed to be present in Tunison Brook, as well as species observed to occur in the St. Regis River of St. Lawrence and Franklin Counties, New York, which drains into the St. Lawrence River (J. McKenna, 2018, personal communication). The latter were included because samples from this study were sequenced on the same flow cell as a set of samples from a different study of St. Regis River eDNA. OTU clustering and picking was performed with all samples from this shared flow-cell, so that potential cross-talk between multiplexed samples could be evaluated (details below). OTU taxonomic assignments are summarized in Table S1.

Several post hoc adjustments were made to taxonomic assignments. A large proportion of 12S reads and a smaller number of COI reads mapped to chicken (*Gallus gallus*). Chicken is a major component of fish feed used at the Tunison facility (Finfish High-Performance, Ziegler), and relative abundance of *G. gallus* 12S reads was strongly correlated with those of *Salmo* and *Coregonus* (Pearson’s *R* = 0.923 for log-transformed sequence counts, *P* < 1*E*-31). The *G. gallus* OTUs were removed from the final analysis. Fish meal is also a component of the feed, which is typically derived from menhaden, *Brevoortia tyrannus* (LaBudde Group, Inc., 2018). Inspection of fish HSPs excluded by our watershed filter revealed the large majority of these to be matches to menhaden. HSPs to pearl dace (*Margariscus margarita*) were considered to be equivalent to daces of the genus *Chrosomus* for the purposes of this study. 12S OTUs matching these taxa were indistinguishable phylogenetically (i.e., had no fixed differences and formed a polytomy in a neighbor-joining tree; File S2). Only pearl dace has been observed in Tunison brook, whereas multiple *Chrosomus* species have been observed in the St. Regis River (J. McKenna, 2018, personal communication). One eukaryotic phytoplankton assignment was reclassified from *Pseudo-nitzschia* to Bacilliaraceae *incertae sedis* (*Pseudo-nitzchia*-like) because *Pseudo-nitzchia sensu stricto* is considered marine but may be paraphyletic with genus *Nitzschia*, which includes freshwater species (Lundholm, Daugbjerg & Moestrup, 2002).

The final abundance of assigned OTUs was determined by re-mapping merged reads to 16S and COI OTUs and counting the number of reads aligning to each at 97% identity, with a maximum of three indel positions and a minimum alignment length of 150. The 12S OTU abundances were counted at 98% identity, with a maximum of two indels and a minimum alignment length of 100. Reads were mapped with bowtie2 using the “end-to-end” and “sensitive” parameter settings. After inspecting the resulting sequence counts, we retained 12S taxa that had 10 or more sequence counts among all study samples and had two or more reads in at least one sample (i.e., were not exclusively singletons). This threshold was based on the distribution of sequence counts of fish known to be present in the St. Regis River (and in fact found in those samples at high levels) but expected to be absent from Tunison Creek. Almost all censored taxa occurred exclusively as singletons in individual Tunison Creek samples, with the exception of one doubleton. While this threshold was empirically informed by the prior knowledge available for fish, it was applied to all 12S taxa including non-fish species. The 12S taxa removed as probable sequencer cross-talk, along with the number of sequence counts from Tunsion Creek samples vs St. Regis River samples, are given in Table S4. Raw counts for all retained taxa by barcode or clade are given in Table S5.

### Statistical analysis

Bacterial sequence counts were collapsed to the family level. Analysis of bacterial diversity was limited to the 150 most abundant families (based on library-normalized abundance), which accounted for >99.9% of bacterial sequence counts. Eukaryotic taxa were collapsed to genus level. For four species-level assignments referenced only by a BOLD database accession and lacking a genus-level rank, an operational genus was used (e.g., “Hymenoptera sp. BOLD:ACC6795” was analyzed as “Hymenoptera genus BOLD:ACC6795”).

Megan v. 5.10.6 (Huson et al., 2007) was used to visualize cladograms of relative sequence counts attributed to taxa, but not for data analysis. Rarefaction, dissimilarity, and homogeneity of dispersion were computed with the R language *vegan* package, v. 2.3-5 (Oksanen et al., 2016). We selected the Morisita index for dissimilarity because it is little influenced by sample size (Morisita, 1962; Wolda, 1981) a key consideration given that the number of sequence reads varied systematically by sampling method. ANOVA, *F*- and *t*-tests, and correlation-based principal components analyses (PCA) were performed with PAST3 (Hammer, Harper & Ryan, 2001). Prior to PCA, taxon relative abundance was logratio-transformed to mitigate the non-linearity of compositional data (Aitchison, 1986), with a pseudocount equal to half the smallest non-zero proportion added to each cell to eliminate zero values. Diversity analyses used raw counts as inputs; however, comparisons of sequence counts across loci and samples were normalized to total sequencing effort using the commonly employed counts per million (cpm) format. Correlation matrices of bacterial cpm were computed with the base R package v. 3.2.2 (R Core Team, 2015) using Spearman’s rank correlation and plotted with the *corrplot* package, v. 0.77 (Wei & Simko, 2017). Code and input files for all R analyses are provided in File S3.

Incidence of individual taxa per sample were calculated directly for each sampler as the number of non-zero abundances divided by the total number of samples considered. To maintain parity of both sample number and filtered sample volume for the calculation of detection rates, we chose to omit some composite samples at both ends of the experimental period to match the number of grab samples available. As there were 22 composite samples at each site but 15 downstream and 18 upstream grab samples, the first three and last four downstream composite samples were omitted, and the first two and last two upstream composite samples were omitted. This approach also maximized the temporal overlap, and thus comparability, of the composite and grab samples.

The abundance of *Salmo* reads relative to *Coregonus* was calculated to assess the precision of each sampling method in estimating the relative amounts of common taxa, assuming the flux of each was approximately constant over the study period. As the primary sources of these two eDNAs are exogenous, and presumably homogenized to some degree in the effluent of the rearing facility, variation in source distributions within the study environment could be disregarded as a contributing factor. We calculated the relative proportion of *Salmo* in each sample as S/(S + C), where S and C are the number of reads assigned at the genus level to *Salmo* and *Coregonus*, respectively. Ratios were calculated for 12S and COI reads separately, and samples with low sequence counts (S + C < 20) were omitted from statistical analysis because of the high standard error of proportions calculated from few observations. In principle, *Salmo* and *Coregonus* reads could have different amplification efficiencies that might affect the ratio S/(S + C). However, we assume any such bias is approximately constant and thus immaterial to the comparison of sampling methods.

## Results

### Patterns of DNA capture

**Figure 2:**
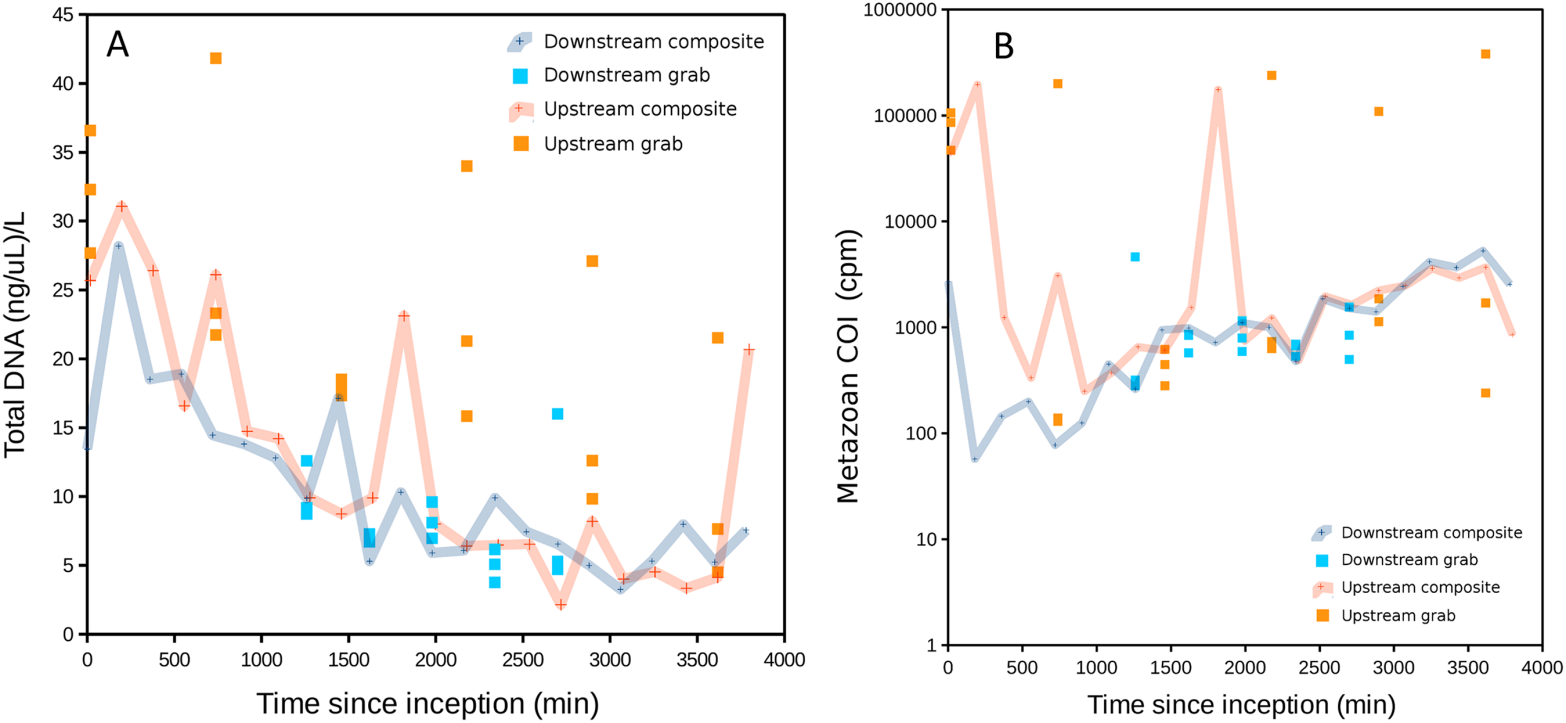
DNA recovered as a function of time of sample initiation, starting at time zero. High values of time indicate samples initiated later in the experimental time series and are approximately the complement of time held in storage in an ice-cooled bin after collection (composite samples are taken over 12 15-min intervals and thus have no singular value for storage duration). (A) Total DNA concentration per filtered volume. (B) Total metazoan sequence counts from the cytochrome oxidase 1 (COI) locus, in counts per million sequence reads (cpm), increase with sample initiation time, or decrease with time held in storage until recovery at the end of the experiment. A similar relationship is also observed for eukaryotic 16S sequence reads but generally not for 12S (see Fig. S1).

The range of DNA concentrations in sample bottles, adjusted to actual filtered volume (approximately 300 mL but slightly less for some samples due to filter saturation), was 2.1–41.8 ng/μL. DNA acquisition was very similar between the two composite samplers, but substantially different between the two grab samplers (Fig. 2A). Upstream grab replicates also showed greater variation than downstream grab replicates.

Total DNA concentration increased with the duration of storage of sample bottles during the experiment, particularly after ∼30 h. The negative correlation between initiation time (approximately the complement of storage time in the autosampler) and DNA concentration was significant for both downstream and upstream composite samplers (Pearson’s *R* = −0.78 and −0.70, respectively, *P* < 0.001 for both). This correlation was significant for the upstream grab sampler but not the downstream one (Pearson’s *R* = −0.67 and −0.22, *P* = 0.0021 and 0.44, respectively). Growth of a flavobacterium that is presumably psychrophilic (i.e., grows competitively at cold temperatures) appears to be a major driver of this pattern, as will be shown in a later section. Bacterial growth within collected samples held in storage within the autosampler would be expected to actively degrade eukaryotic eDNA as well as supplant it when DNA extracts are normalized to a common concentration. Indeed, a scatterplot of COI sequence count rate (cpm reads) versus time of sampling indicates that COI count rates in initial samples were less than a tenth of that in samples collected at the end of the experiment (Fig. 2B), consistent with degradation of eDNA as a function of time held in storage. A similar pattern was observed for eukaryotic 16S sequence counts, but much less so for the shorter 12S locus. This difference may reflect proportionally greater decay of the longer loci relative to 12S.

### Sequencing output and taxa identified

The number of read pairs passing filter and successfully demultiplexed by the sequencing software was 20.69 million. After trimming exogenous sequence and low-quality bases, 20.66 million pairs remained with an average length of 231.4 bp. The number of read pairs successfully merged, mapped to COI or 16S OTUs, and assigned a taxonomy was 11.67 million, whereas an additional 0.39 million forward reads were assigned a 12S taxonomy.

Use of equal volumes of each amplicon in the library prep resulted in excessive 16S sequences at the expense of 12S and COI sequence reads. The number of bacterial 16S sequence counts per sample averaged 136,634 (SD 46,979). COI sequence counts per sample averaged 2,076 (SD 5,661), eukaryotic 16S sequence counts averaged 32 (SD 44), and 12S sequence counts averaged 831 (SD 1,545). The higher variance of eukaryotic sequence counts is due in part to sequence counts well above the median in some upstream samples (Fig. 2B). High variation in COI and 12S sequence counts also reflects the differential amplification success of these barcodes that was evident in gels (see ‘Materials and Methods’): 12S and COI sequence counts were rarely similar in scale in a sample, with one or the other predominating (Fig. S1). 12S sequence counts tended to be higher than COI in the earliest samples initiated, which again may reflect proportionally greater decay of the longer COI locus with length of storage in the autosampler. A table of sequence counts by taxon and sample is provided in Table S5.

The eukaryotic taxa identified by the three barcodes are summarized in Fig. S2. The fish genera *Salmo* and *Coregonus* were the dominant taxa in most samples, as expected. Other vertebrates were generally rare. Invertebrate sequence counts were dominated by several genera of non-biting midges (Insecta: Diptera: Chironomoidea). Smaller numbers of reads were derived from amphipods (e.g., *Gammarus*), copepods (e.g., *Acanthocyclops*), and annelids (e.g. *Nais*). A phylogram of bacterial taxa is provided in Fig. S3 and patterns of bacterial distributions are further detailed in a later section. Note that we did not sum 12S and COI sequence counts with the same taxonomic assignment because they represent distinct barcodes with different amplification dynamics (Fig. S1). “*Salmo*-COI” and “*Salmo*-12S,” for example, were considered distinct eDNA classes for the purpose of comparing sampling methods.

### Comparing variance in relative abundance of common fish eDNAs

**Figure 3:**
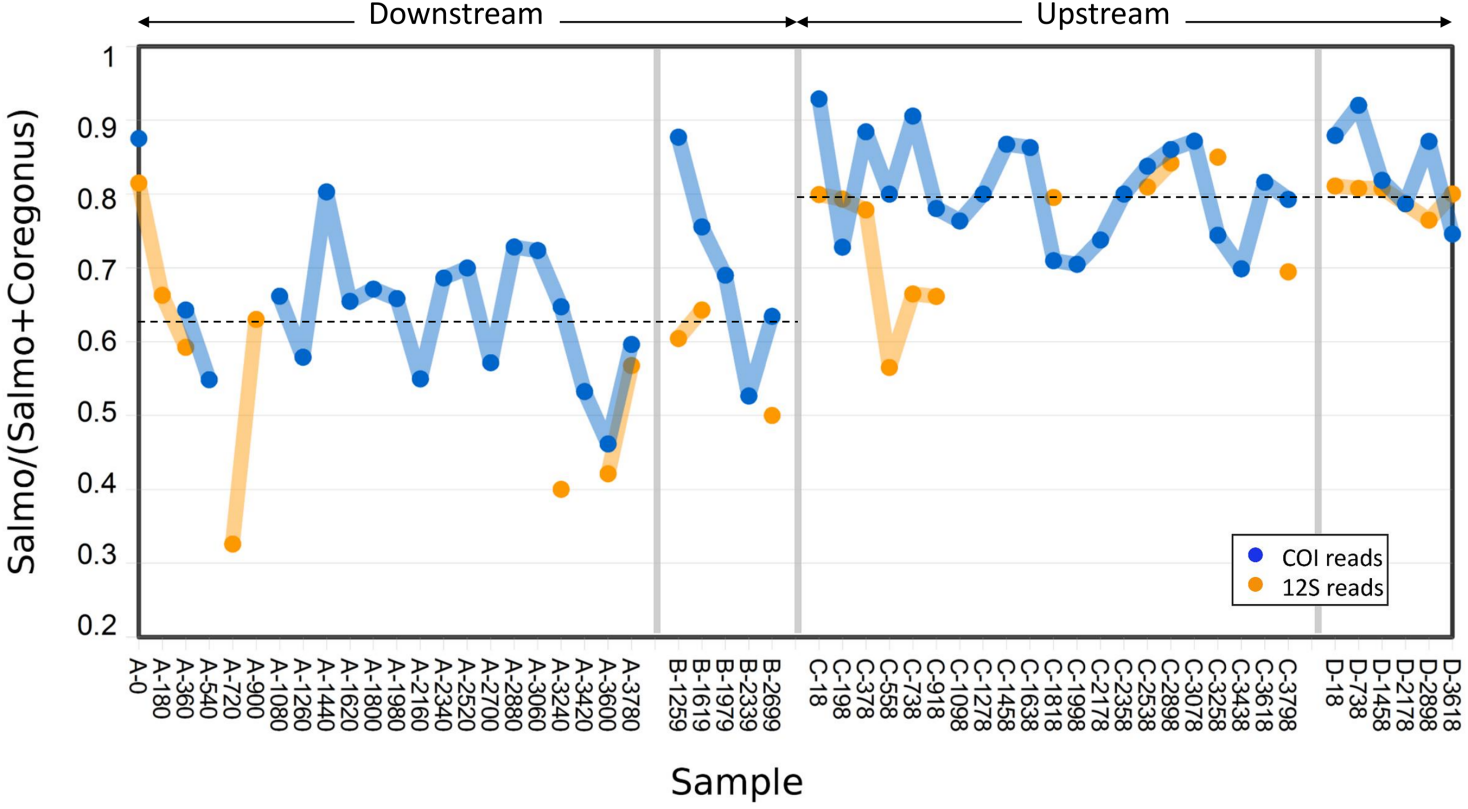
Abundance of Atlantic salmon (genus *Salmo*) sequence reads relative to Cisco and Bloater (genus *Coregonus*) sequence reads at upstream and downstream sites. Grab replicates are pooled. Relative abundance estimates from 12S and COI sequence counts are plotted separately, with the mean values at each site indicated by dashed lines. No significant correlation between relative Salmo eDNA abundance and time was found, whereas the difference in means between sites was statistically significant (see Results).

We calculated the relative abundances of *Salmo* and *Coregonus* reads in each sample to assess the precision of a repeated quantitative estimate by sampling method (Fig. 3). All samples had *Salmo* COI and 12S reads, as well as *Coregonus* COI reads, whereas *Coregonus* 12S reads were detected in 70 of 77 samples. However, we omitted from statistical analysis any estimate that was based on fewer than 20 total reads (since ratios derived from few observations have high error). As the relative abundance of *Salmo* was consistent between loci (Pearson’s *R* = 0.67, *P* < 0.001), we pooled estimates from both for statistical analysis. We also pooled estimates from the two sites by sampling method, after first centering the values at each site around zero; this assumes that variances have the same distribution across sites and any difference is due to sampling method.

The variance in *Salmo* relative abundance was not statistically different between the two sampling methods (Table 1). Although both sampling methods estimated the mean relative abundance of *Salmo* eDNA with similar precision, the means themselves differed between downstream and upstream sites (Fig. 3). The average relative abundance of *Salmo* was 0.627 (SE 0.021) downstream and 0.794 (SE 0.011) upstream. Equality of means among the four sets of samples was rejected by two-way ANOVA (Table 2), with site highly significant (*P* < 0.001) but not sampling method (*P* > 0.05). This observation implies that particles bearing *Salmo* eDNA settle or decay faster than do those bearing *Coregonus* eDNA, since these classes of eDNA derive from the rearing facility effluent upstream and pass the two sites sequentially. Ratios were not significantly correlated with time of sample initiation at either site (Pearson’s *R* = 0.02 downstream and 0.12 upstream, *P* > 0.50 at both sites), indicating stability of eDNA proportions over the study period.

**Table 1:** *F* test of heterogeneity of variance by sampler type in the estimation of *Salmo* relative abundance.

*F* test parameter	Composite samples	Grab samples
*N*	59	20
Variance	0.010	0.007
*F*	1.4961	
*P*	0.333	

**Note:**

Results are shown for samples combined across sites, after centering the distribution at each site.

**Table 2:** Two-way ANOVA of estimates of *Salmo* relative abundance, grouped by site and sampling method.

ANOVA parameter	Sum of squares	d*f*	Mean square	*F*	*P*
Site	0.547559	1	0.547559	55.86	1.189*E*-10
Sample type	0.0265163	1	0.0265163	2.705	0.1042
Interaction	0.0015004	1	0.0015004	0.1531	0.6967
Within	0.735201	75	0.00980268		
Total	1.29862	78			

### Comparing incidence and richness by sampling method

**Figure 4:**
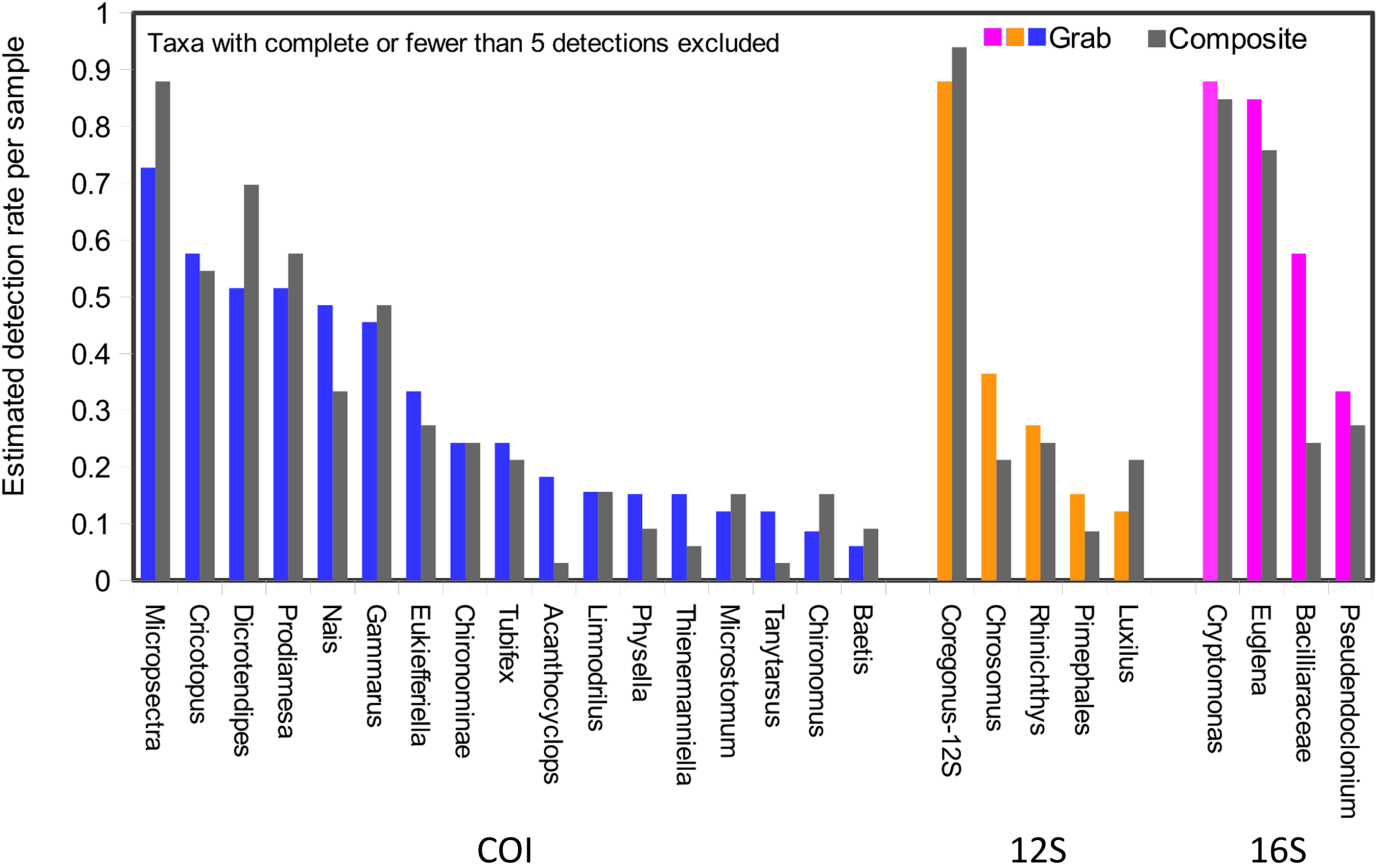
Detection rates per sample unit by method. Taxa detected by grab sampling are represented by colored bars, with each locus colored distinctly, whereas the corresponding gray bars represent composite samples. Note that 16S OTUs assigned as eukaryotes derive from the chloroplast organelle. Detection rates are combined across upstream and downstream samplers of each type, and sample size was trimmed to an equal effort of *N* = 33 filtered sample bottles for each method (see ‘Materials and Methods’ for details), with maximum temporal overlap.

**Figure 5:**
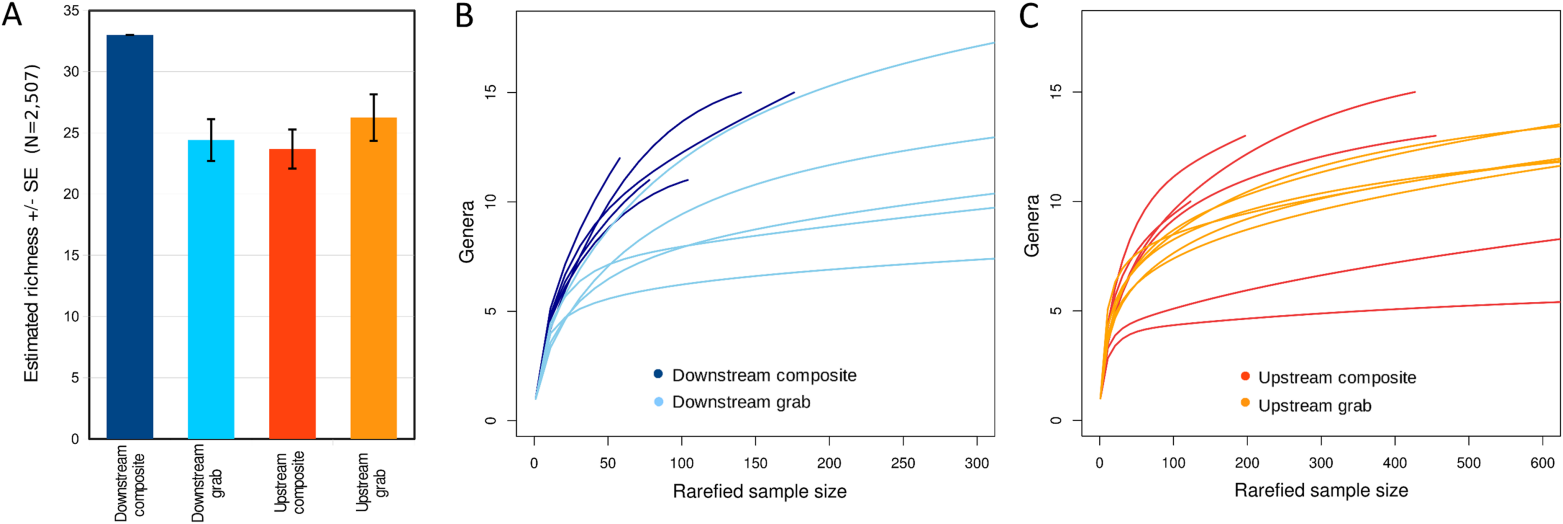
Expected eukaryotic richness by site and sampling method with rarefaction, considering all loci together. (A) Expected total richness with standard errors, calculated at the genus level for 15 downstream or 18 upstream sample units per sampling method. Expected total richness reflects rarefaction to the smallest total eukaryotic sequence counts of the four samplers, which was *N* = 2,507 from the downstream composite sampler. The standard error for this sampler is therefore zero by definition. Sample sizes are the same for each method at a site but not between sites, as there is no expectation that richness should be the same between sites. (B) Taxon accumulation curves for downstream time-matched samples. Curves represent expected richness under different levels of rarefaction, incremented in steps of 10 sequence counts. (C) Taxon accumulation curves for upstream time-matched samples.

**Figure 6:**
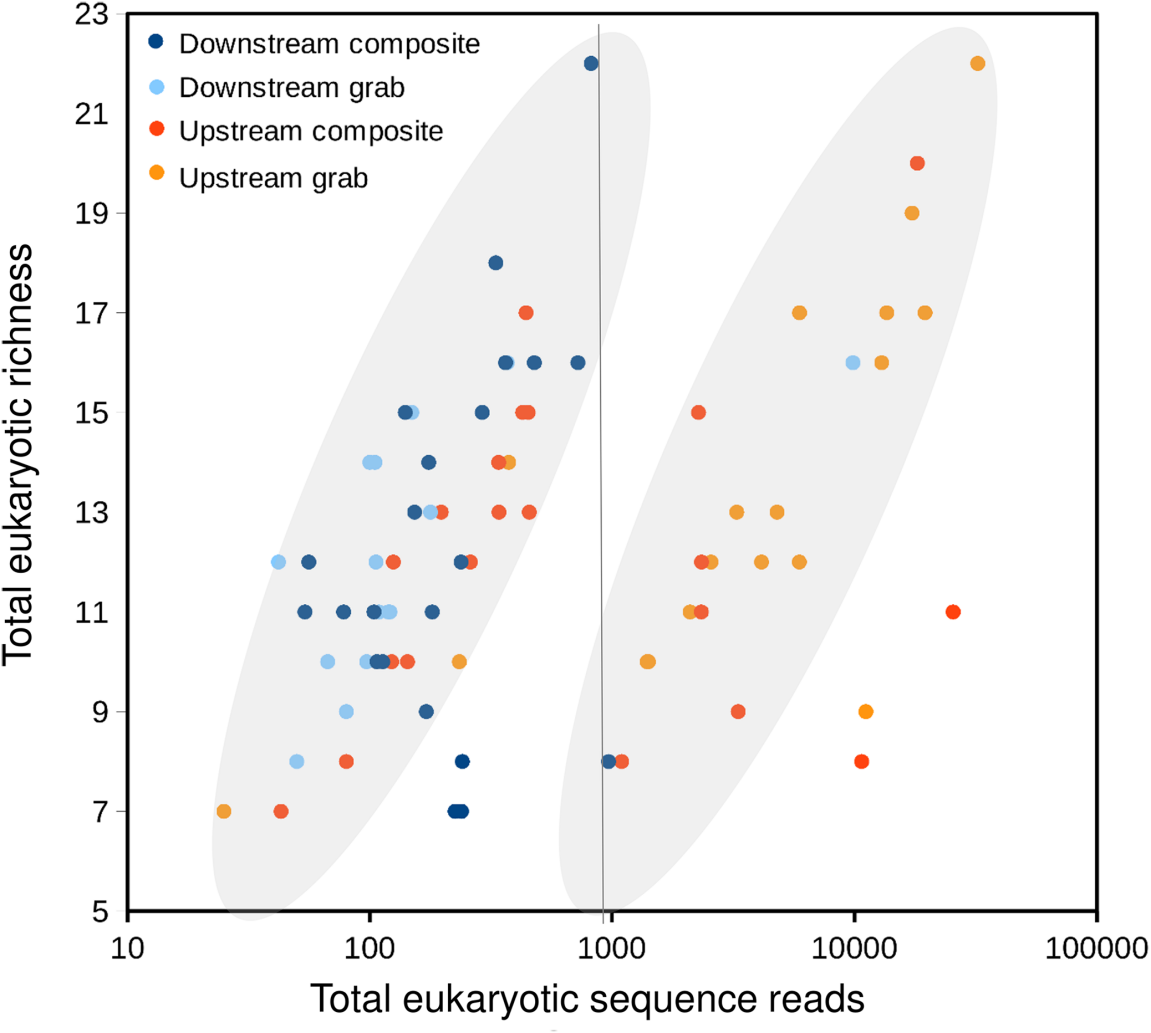
Scatterplot of eukaryotic richness versus eukaryotic sequence counts, log-scaled, by sampler. The gray ovals highlight a natural break in the distribution of points, between modes of relatively low and relatively high sequence counts for equivalent levels of richness. That is, samples plotted to the right of the vertical line as a group have accumulated richness more slowly per sequencing effort than those plotted to the left of the line. While some positive relationship between sequence counts and richness per sample is expected, the existence of two distinct populations of points is subjective and hypothetical, and does not imply that explicit mathematical functions relating sequence counts to richness have been derived.

Taxonomic recovery by sampling method was assessed by estimating taxon-specific detection rates per sample and by estimating paired-sample and total sample-set richness under rarefaction. Estimated detection rates for specific eukaryotic taxa were not consistently higher for either sampling method (Fig. 4). Estimated total richness for the same filtered volume (*N* = 15 samples downstream, *N* = 18 samples upstream, see ‘Materials and Methods’ for details), rarefied to the smallest number of eukaryotic sequence counts (*N* = 2,507 for the downstream composite sampler), was higher for composite than grab samples at the downstream site (Fig. 5). Estimated total richness at the upstream site was higher for grab than for composite sampling, but the standard errors of the two estimates overlapped. Taxon accumulation curves for individual composite samples generally showed equal or more rapid accumulation of taxa than pooled triplicate grab samples, with the exception of two upstream composite samples that had low richness despite high sequence counts. Plotting the eukaryotic richness of all samples as a function of total eukaryotic sequence counts further suggested the occurrence of two distinct modes of count-richness relationship (Fig. 6), primarily impacting individual upstream samples of both types. These observations are also consistent with high values of total DNA in some upstream samples (Fig. 2A**)**. High total DNA coupled with low eDNA diversity may indicate the presence of clumped eDNA particles swamping eDNA of other origins. However, fewer composite samples showed this pattern than did grab samples (Fig. 6). For bacterial taxa, expected richness of samples rarefied to 50,000 sequence counts were similar between the two sampling methods (Fig. S4). Stratifying bacterial taxa into three tiers based on relative abundance affected the variability of estimated richness generally, in terms of increasing standard errors as progressively rarer taxa were included (Fig. S4), but did not affect the equivalence of the two sampling methods in this regard.

### Comparing the precision of taxon relative abundances

**Figure 7:**
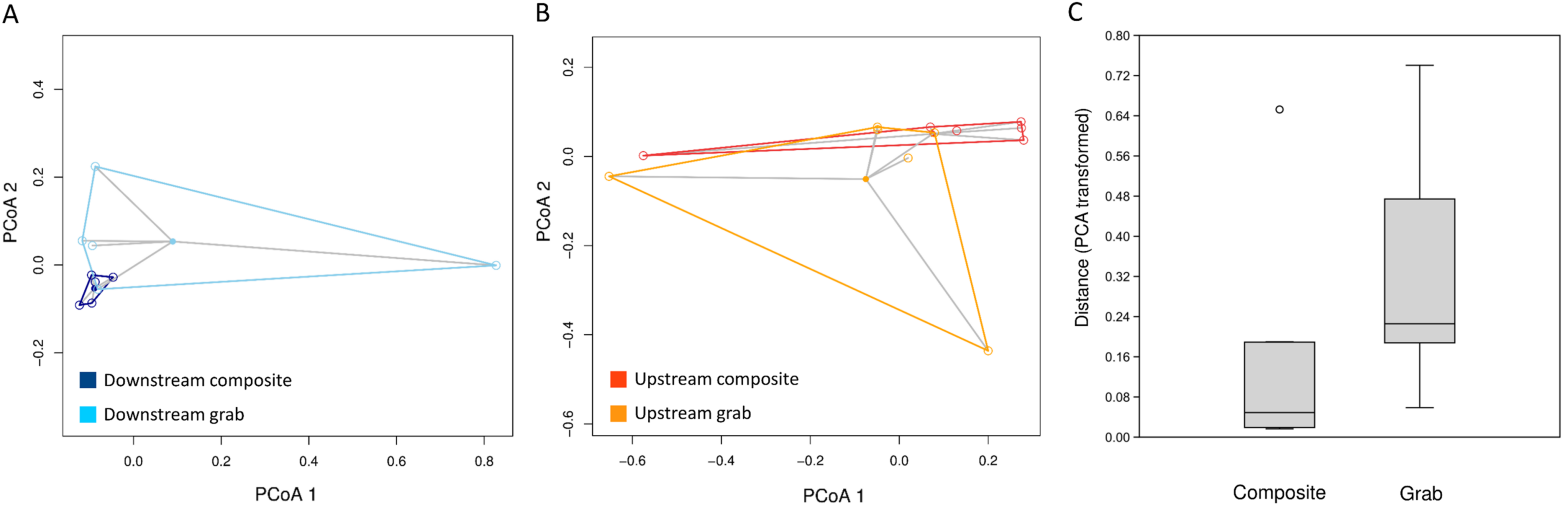
Dispersion of samples around the centroid value of pairwise dissimilarity (Morisita’s index). Relative distances in (A) and (B) are visualized using the first two axes of the PCA transformation, whereas multivariate distance from the centroid for statistical analysis (C) is based on all PCA axes. (A) Downstream site, with composite samples in dark blue and grab samples in light blue. (B) Upstream site, with composite samples in dark orange and grab samples in light orange. (C) Combined mean distances from centroids, by sampling type.

The consistency of taxon relative abundances among samples was evaluated by calculating pairwise dissimilarities and determining the dispersion of these values around the centroid value of each sampling method. For eukaryotic sequence counts, Morisita dissimilarities among time-matched samples (Fig. 7) had significantly lower dispersion around the centroid for composite samples than for pooled grab replicates (*P* < 0.01 for both permutation *t* test and Mann–Whitney non-parametric test), whereas no effect of sampling method was found for bacterial relative abundance (Fig. S5). The pattern observed for bacterial taxa again held true when considering either common or rare taxa: dispersion plots for the bacterial taxa with rank abundance 51–100 and 101–150 (out of 164 total) are comparable to those for the top 50 taxa, and no significant differences in dispersion around the centroid were found between sampling methods.

### Bacterial profiles differentiate sites and samples

At both sites, the relative abundances of most bacterial families were positively correlated with each other but negatively correlated with a small number of taxa, particularly Flavobacteriaceae, (Fig. S6). Overall, Flavobacteriaceae was the bacterial taxon most negatively correlated with sampling time during the experiment (i.e., the earliest collected samples had higher levels of Flavobacteriaceae, *N* = 77, Spearman’s rho = −0.89, *P* < 0.001). These data suggest that growth of Flavobacteriaceae contributed most to the displacement of other microbes and eDNA sources discussed previously.

**Figure 8:**
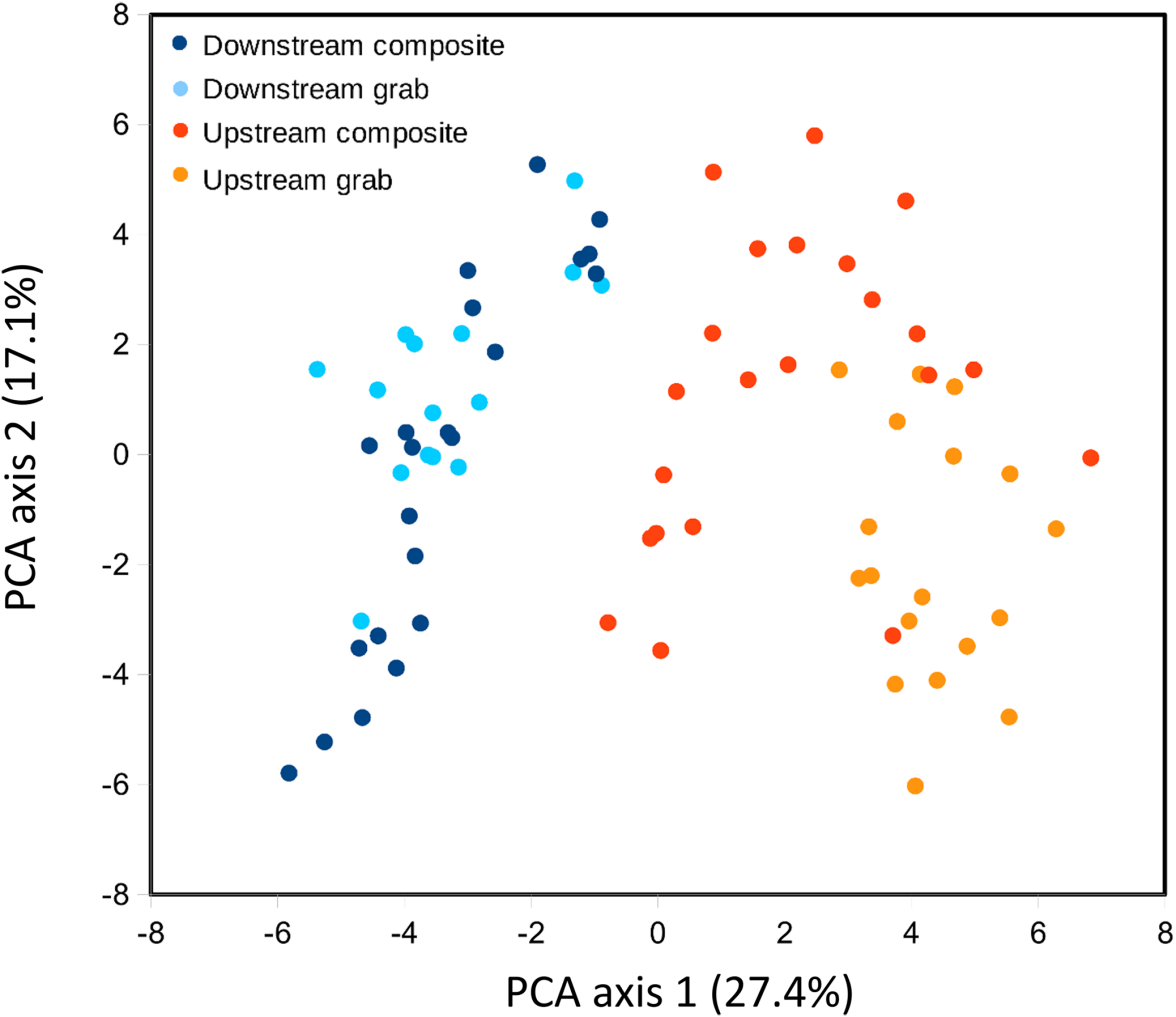
Scatterplot of sample scores by sampler for the top two axes resulting from PCA of the relative abundance (log-ratio scaled) of the top 50 bacterial families.

Ordination was used to evaluate whether samples recovered comparable bacterial profiles, as a marker of potential microenvironmental heterogeneity. PCA of the top 50 bacterial taxa by relative sequence abundance (log-ratio transformed) identified two axes accounting for 44.5% of the total variation (Fig. 8). The first axis separated upstream from downstream sample sets, and further separated upstream grab and upstream composite sample sets, whereas downstream sample sets had similar ranges. By this measure, the two upstream samplers appeared to be sampling different microenvironments, which may have impacted the taxa and richness of recovered eukaryotic eDNA.

**Figure 9:**
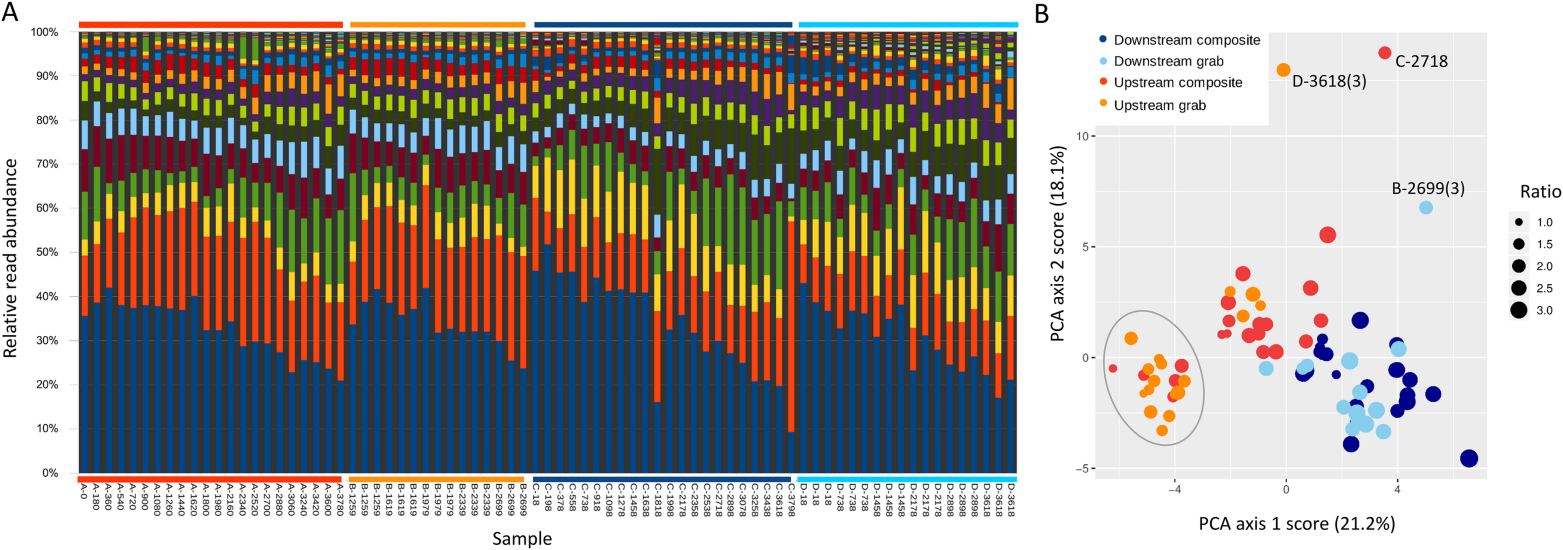
Associations between the top 50 Flavobacteriaceae OTUs and sample collection time and site. (A) Proportional composition of Flavobacteriaceae OTUs in each sample (unit-scaled), revealing a single OTU (bottom blue bars) that is most strongly associated with time of storage in autosamplers. Note each sample name combines the sampler code with the time of sample initiation in minutes, and is therefore time-ordered within each sampler (see Fig. 1 for sample codes). Thus, the earliest-initiated samples were held in storage for the longest period of time. The set of samples derived from each sampler is further delineated by colored bars above and below the plot to help distinguish patterns within and among samplers. (B) Scatterplot of sample scores for the first two PCA axes of relative abundance of Flavobacteriaceae OTUs. Sample labels indicate the replicate number in parentheses, if applicable, and are used to identify individual outliers with low total 16S sequence counts. The circled set of points are all to the right of the line in Fig. 6 that demarks a collection of samples with high sequence counts but not correspondingly higher richness. The size of each point is scaled to the ratio of eukaryotic richness to the natural log of eukaryotic sequence counts, as indicated by the legend.

We examined the relative abundance of OTUs within the Flavobacteriaceae to assess whether specific OTUs contributed disproportionately to the increase in Flavobacteria with storage time. This change was in fact largely attributable to a single OTU (Fig. 9A), indicating that this taxon was strongly competitive under the psychrophilic storage conditions. We also performed a PCA of the top 50 OTUs in this bacterial family by relative read count. A scatterplot of samples by score on the first two PCA axes (Fig. 9B) recovered a division among upstream samples similar to that seen in Fig. 6, in the sense that samples in the cluster of points with higher sequence counts on the right side of Fig. 6 are largely the same as those separated from the main cluster of points in Fig. 9B (circled in that figure). The points in Fig. 9B are weighted by the ratio of eukaryotic richness to the natural log of eukaryotic sequence counts. This pattern suggests that lower-richness samples tend to have a distinctive bacterial profile that is not accounted for by sampler location alone. Note that three samples are labeled as outliers in Fig. 6B that also had low total 16S sequence counts (Table S5), which may have affected compositional correlations.

## Discussion

### Comparison of grab and composite sampling

For some eDNA metrics, the composite sampling scheme implemented here performed better or more consistently than did a typical grab sampling approach. Morisita dissimilarities among repeated samples were lower for composite than grab sampling (Fig. 7), indicating that relative abundance of taxa in sequence reads was assessed more precisely. Total richness in an equal number of water samples was higher with composite sampling at the downstream site (Fig. 5A), and taxon accumulation under rarefaction was faster for individual composite samples at that site than the pooled grab samples they were paired with (Fig. 5B), despite being derived from one-third the filtered volume. At the upstream site, neither total richness nor sample richness consistently differed by sampling scheme (Figs. 5A and 5C). However, the aquatic community collected by the two upstream samplers may not have been entirely equivalent based on differences observed in their bacterial profiles (Figs. 8 and 9B), discussed further below.

While composite sampling appeared to produce more representative taxonomic profiles, there was no gain in the frequency of presence detection for specific taxa, whether rare or common overall (Fig. 4). Grab sampling also acquired the most common eDNA classes, namely *Salmo* and *Coregonus*, in proportions similar to composite sampling (Fig. 3). A caveat of the latter result is that these eDNAs classes entered the stream through the effluent and therefore were likely more evenly mixed than if they had derived from an equivalent number of fish distributed in the actual study environment. Our finding that the two sampling methods produced similar estimates of relative *Salmo* eDNA abundance may therefore not hold in the latter scenario. Even so, composite sampling seems likely to be most useful for applications that are sensitive to relative abundances of diverse eDNAs, such as distance-based analysis of OTU tables.

Importantly, rarity of DNA classes per se did not seem to be important in distinguishing the two sampling methods, as they showed similar dispersion characteristics for both common and rare bacteria (Fig. S4). This implies that unevenness in the distribution of eukaryotic DNA is the more important factor. It is also worth emphasizing that grab sampling required greater sequencing effort (and thus cost) than time-matched composite samples, as each was a pool of three sequenced replicates. In principle, the grab replicates could be combined at several stages prior to sequencing, thereby reducing this drawback. However, rare templates are likely to be lost using this approach if sequencing effort is held fixed (Sato et al., 2017), reducing the diversity detected. On the other hand, our composite-sampling approach required that a specialized and expensive device be left unattended, and its deployment was further constrained by practical considerations such as power supply, number and volume of sample bottles, and positioning of intake tubing. Composite sampling of this nature will only benefit when the potential gains exceed the added costs and constraint, but of course we cannot generalize from one comparison in one environment how this threshold might be judged. Instead, it seems reasonable to recommend that pilot studies for the evaluation of marker loci, eDNA capture, eDNA preservation, and the like also include a spatially and temporally dense series of samples from a test environment, regardless of the acquisition method. Dense sampling can help clarify the distribution of targeted eDNAs, providing a valuable baseline for informing sampling design to meet specific objectives, within financial or logistical constraints. Despite their limitations, stock autosampling equipment such as that used here may be useful for such assessments.

### Other sources of sample variability

Our methodological comparison assumes that equivalent communities were being sampled by each pair of samplers, such that differences can be attributed to method rather than environment. We placed autosampler intakes as close as was feasible without risking fouling, but microenvironmental heterogeneity could have influenced the results. For example, the upstream samplers were approximately eight meters below the site of effluent entry and close to a small spillover dam (Fig. 1), and also experienced higher flows than the downstream site. These hydrological factors could have increased environmental heterogeneity at the upstream site relative to downstream, as suggested by lower among-taxa correlations for bacteria upstream (Fig. S6). Ordination of bacterial composition (Fig. 8) indicated that the two upstream communities were not only more variable but distinct from each other, and therefore potentially distinct with respect to eukaryotic eDNA as well. Of course, distinctiveness of sampled environments does not automatically imply measurable differences in richness or dispersion. Microcosms of known composition and controlled hydrology would be useful for further disentangling sampling method from environmental heterogeneity, but for the current work, we were keen to evaluate differences in a natural environment. It remains possible that differences in eDNA acquisition among samplers is attributable to unknown machine characteristics, rather than sampling scheme per se, as reciprocal runs were not feasible. However, any errors detected by the autosampler during operation would have been logged by the controller, and final volumes within recovered sample bottles were as expected. While these data give cause to interpret the upstream results more cautiously, the observed differences between sample types were broadly similar at both locations in several respects, and it was the downstream site that showed the most significant differences in eukaryotic eDNA between sampling methods, as well as strong concordance of bacterial profiles.

Further evidence of heterogeneity at the upstream site comes from the distribution of eukaryotic sequence counts relative to eukaryotic richness recovered for each sample (Fig. 6). While a positive monotonic relationship between sequencing depth and sample richness is expected, we identified a natural break in the distribution of sample points in Fig. 6 that suggested two distinct empirical relationships among samples. Samples on either side of the break fall in the same overall range of richness, but differ greatly in sequence counts. Assuming actual richness was not this variable during the experiment, an alternative explanation for the observed pattern may relate to the degree of physical aggregation of eDNA particles. As input DNA concentrations were diluted to a common value prior to barcode amplification, more clumped eDNA-bearing particles would produce lower richness values for a given sequencing depth. Variation in the physical aggregation of eDNA particles is not unexpected given the various biological mechanisms by which eDNA may be shed. Importantly, samples fall to the right side of the break in very different proportions among the four autosamplers: 1 of 22 downstream composite samples, 1 of 15 downstream grab samples, 8 of 22 upstream composite samples, and 15 of 18 upstream grab samples. These results again suggest that the downstream sample sets were more comparable to each other than the upstream ones. They also indicate that upstream composite samples tended to have higher richness per sequencing effort than did upstream grab samples, suggesting that composite sampling mitigated this swamping effect.

### Other technical considerations and caveats

This study focused on the effects of sampling scheme only and did not employ strict sample containment and preservation methods to maximize eDNA yield and minimize contamination. We do not believe eDNA degradation or carryover is material to this comparison as long as it is equivalent across sample types. Furthermore, the number of sequence counts are intrinsically linked to variation in barcode amplification, sequencing library quality, and library loading. These processes act to decouple total input DNA quantity from the number of sequence reads recovered per sample. While optimization of sample containment, preservation, and barcode design remain critical areas of research, they could not be meaningfully addressed within the scope of this study.

We did not attempt to assess the accuracy of taxonomic recovery or assignments for this study (e.g., with the use of mock communities, spike-ins, or negative controls). We believe the objectives of this study are not predicated on accurate taxonomic assignment, and again assume that such errors are random with respect to sampling scheme. Indeed, OTU picking, counting, and assignment are performed for a run collectively and not separately for each autosampler. On the other hand, differences between sampling methods could conceivably have been influenced by the level of taxonomic binning that was used (i.e., at genus level for eukaryotic eDNA). For example, the genus assignments *Salmo* and *Coregonus* could both have conflated multiple species, thereby reducing the potential richness of samples. However, taxonomic binning is commonly done, and species-level resolution is often difficult to achieve with short barcodes (Yu et al., 2012; Ji et al., 2013; De Barba et al., 2014; Stat et al., 2017), so we believe our approach is relevant to actual practice. We suspect that finer taxonomic resolution would typically act to strengthen differences already observed between sampling methods at a coarser taxonomic resolution, rather than decrease them, but this remains to be demonstrated (by analyzing mesocosm communities of known composition, for example).

For time-matched comparisons (Figs. 5B, 5C and 7), we paired single composite samples with grab triplicates by the time they were initiated, to assess the performance of sample compositing over the following three hours versus the grab replicates taken in immediate succession. This seems to us an apt comparison, as three hours falls within the range of time practitioners might reasonably spend at a site, particularly if sites are infrequently visited and other types of data are collected as well. However, other possible paired comparisons could be made given the structure of our observational time series (Fig. 1C), such as pairing the composite sample initiated three hours *previous* to the initiation of each grab replicates, or comparing equal time intervals using samples from the beginning and end of each interval. While these may be legitimate ways to pair samples by time of collection, we chose our approach because it provided good replication of alternative strategies likely to be implemented in practice.

It would be of interest to determine whether the predominance of Chironomidae among COI reads reflects genuine abundance of this class of eDNA during the study period, or ascertainment biases such as preferential amplification (Clarke et al., 2014) or a more accurate taxonomy. Chironomidae are in fact highly abundant in Appalachian streams and shed exuviae (pupal cases) onto water surfaces at maturation (Hynes, 1970; Wetzel, 1975; Pinder, 1986), such that they may indeed be a dominant invertebrate eDNA source. Interestingly, Bista et al. (2017) also obtained excellent amplification of Chironomids with COI eDNA markers and argued for their value as efficient genetic markers of biodiversity; morphological analysis of exuviae has long been used for such assessments (Wilson & Bright, 1973). Regardless of the underlying cause of skew, it may be preferable to use a suite of narrower barcodes that target distinct indicator groups (Balasingham et al., 2017). Such an approach was recently described for invertebrate clades of the Great Lakes (Klymus, Marshall & Stepien, 2017), for example. Another difficulty of invertebrate metagenetic taxonomy we encountered was that plausible, high-scoring taxonomic assignments deriving from the comprehensive, but very loosely curated, NCBI nt database were absent from the more curated BOLD COI accessions at the time of this analysis (see Materials and Methods). Thus, taxonomic assignment algorithms that could potentially improve upon the heuristic LCA approach are unlikely to be very effective for North American invertebrates without concomitant improvement of reference databases.

We interpret the significant difference in relative *Salmo* eDNA abundance at upstream vs downstream sites as a reflection of faster settling and/or decay of *Salmo* eDNA. We assume that any in situ production of these eDNA types is trivial relative to the contribution from the Tunison effluent, and we have no reason to expect that the latter source changed appreciably in composition over the course of our observations. Had the latter been the case, the expected effect would have been a temporal trend in relative Salmo eDNA abundance at the upstream site and likely the downstream site as well, yet values at both sites were uncorrelated with time. While we certainly expect eDNA persistence to differ by species—for example, fecal settling rate is frequently monitored in the context of aquaculture management and may vary by species or with diet (Magill, Thetmeyer & Cromey, 2006)—the relative rate of *Salmo* dropout evident at 100 m in a flowing stream was unexpected to us. If commonplace, species-specific effects of this scale could complicate interpretations of relative sequence abundance and spatial distribution of aquatic eDNA, such as the mapping of individual species, communities, or ecological transitions in an environment (O’Donnell et al., 2017).

### Potential uses of bacterial profiles

While the concentration of the bacterial amplicon used in this study should clearly be attenuated when eukaryotic eDNA is of primary interest, our results suggest that bacterial signatures may in fact be useful internal controls, for example, to detect samples that have experienced different handling or derive from distinct microenvironments. For example, the proportion of Flavobacteriaceae was strongly correlated with duration of storage within autosamplers, attributable to a single dominant OTU (Fig. 9A). Ordination of Flavobacteriaceae OTUs (Fig. 9B) also differentiated upstream samples previously identified as having relatively low richness (Fig. 6), suggesting that bacterial composition can reveal samples impacted by highly clumped eDNA sources. Speculatively, these compositional differences could have been derived from the microbiome of the reared fish (Loch & Faisal, 2015; Lowrey et al., 2015) or otherwise enriched in the effluent relative to the native stream. While our results generally concord with an expected lower sensitivity of bacterial profiles to sampling method than eukaryotic profiles, it does not follow that shifts in the former are invariably linked with shifts in the latter. Any proposed biomarker application would require empirical validation. Unfortunately, our limited understanding of the ecology of environmental bacteria and the potential for closely related species to be found in very diverse environments (McBride et al., 2014) augur that the interpretation of outliers will often be ad hoc and not necessarily transferable among environments.

## Conclusions

Comparison of one possible composite sampling scheme for eDNA with a typical grab sampling approach provided more consistent recovery of eukaryotic taxon proportions, as assessed by multivariate distances. Composite sampling also recovered greater taxonomic richness at one of the two sites, and captured more consistent levels of total DNA between sites. The two methods produced comparable presence-absence detection rates of individual taxa and estimates of the relative abundance of the two dominant fish taxa were comparable. Our data suggest that microbial profiles as indicators of sample representativeness are worth further exploration, and highlight the potential for differential eDNA settling over relatively small scales to impact estimates of relative abundance and spatial distribution.

## Supplemental Information

10.7717/peerj.5871/supp-1 Supplemental Information 1 Perl-style pseudocode of LCA assignment procedure.

10.7717/peerj.5871/supp-2 Supplemental Information 2 Fasta-formatted12S OTU representative sequences for matches to the fish genera Chrosomus and Margariscus and a phylogeny of those sequences.

10.7717/peerj.5871/supp-3 Supplemental Information 3 R code and input data for all R analyses.

10.7717/peerj.5871/supp-4 Supplemental Information 4 Summary of OTU representative sequences and high-scoring database matches.

10.7717/peerj.5871/supp-5 Supplemental Information 5 Summary of bacterial taxonomic assignments adjusted to conform with NCBI nomenclature.

10.7717/peerj.5871/supp-6 Supplemental Information 6 List of fish taxa used to constrain taxonomic assignments at the 12S locus.

10.7717/peerj.5871/supp-7 Supplemental Information 7 Summary of 12S taxa removed as potential contaminants.

10.7717/peerj.5871/supp-8 Supplemental Information 8 Summary of sequence counts by taxon and sampler, for each barcode.

10.7717/peerj.5871/supp-9 Supplemental Information 9 Sequence abundance by barcode locus. Counts by locus for each sample are shown in panel A and relative amounts of 12S and COI reads are shown in panel B.

10.7717/peerj.5871/supp-10 Supplemental Information 10 Phylogram of eukaryotic taxa identified at each barcode locus. Size of circles is proportional to the log of sequence counts and colored according to the legned. The fish genera Salmo and Coregonus were recovered at both the 12S and cytochrome oxidase 1 loci.

10.7717/peerj.5871/supp-11 Supplemental Information 11 Phylogram of bacterial taxa recovered at the 16S barcode locus. Size of circles is proportional to the square-root of sequence counts, in counts per million and summed across samples.

10.7717/peerj.5871/supp-12 Supplemental Information 12 Expected richness of bacterial taxa in time-matched samples. Richness of bacteria by sampler after stratification into three tiers of overall rank abundance. Rarefaction value is the minimum total count among the four samplers for each comparison. Upstream and downstream sites are shown separately (panels A–E).

10.7717/peerj.5871/supp-13 Supplemental Information 13 Bacterial dispersion around the centroid of pairwise Morisita dissimilarity. Dispersions are shown for each sampler as indicated by the legend, for all taxa as well as progressive tiers of relative abundance.

10.7717/peerj.5871/supp-14 Supplemental Information 14 Correlation matrices of rank abundance of bacterial families by site. Matrices represent Spearman rank correlations between pairs of taxa, the strength and sign of which are indicated by the color scale and the size of the square in each matrix cell.

## Additional Information and Declarations

### Data Availability

The following information was supplied regarding data availability: NCBI Short Read Archive, BioProject accession: PRJNA431582.
