# Supplementary material for: An experimental comparison of composite and grab sampling of stream water for metagenetic analysis of environmental DNA
Source: PeerJ. 2018 Dec 5;6:e5871. doi: 10.7717/peerj.5871 (PMC6286662; doi:10.7717/peerj.5871)

File S2. Part A: Alignment of 12S OTUs with top reported HSPs to *Phoxinus eos* (synonymous with *Chrosomus eos*) and *Margariscus margarita*. Headers are of the form >OTU representative sequence ID:Top HSP sequence header (*nt* nucleotide database).

>M01138:27:000000000-AHTAW:1:1102:17165:8483:dbj|AP009151.1| Phoxinus eos mitochondrial DNA complete genome

TAGAACAGGCTCCTCTAGGGGGGGTTTAAGGCACCGTCAGGTCCTTTGGGTTTCAAGCTA

ATGCTCGTAGTACCCGGGCGGACGTCTAATTGTAGTTGGACATCTAGGTTTATTGCTGAG

CATAGTGGGGTATCTAA

>M01138:27:000000000-AHTAW:1:2115:8081:6120:dbj|AP009151.1| Phoxinus eos mitochondrial DNA complete genome

TAGAACAGGCTCCTCTAGGGGGGGTTTAAGGCACCGCCAGGTCCTTTGGGTTTCAAGCTA

ATGCTCGTAGTACTCGGGCGGACGTCTAATTGTAGTTGGACATCTAGGTTTATGGCTGAG

CATAGTGGGGTATCTAA

>M01138:27:000000000-AHTAW:1:2102:21740:23686:dbj|AP009151.1| Phoxinus eos mitochondrial DNA complete genome

TAGAACAGGCTCCTCTAGGGGGGGTTTAGGGCACCGTCAGGTCCTTTGGGTTCCAAGCTA

ATGCTCGTAGTACCCGGGCGGACGTCTAATTGTAGTTGGACATCTAGGTTTATGGCTGAG

CATAGTGGGGTATCTAA

>M01138:27:000000000-AHTAW:1:1101:10004:4587:dbj|AP009151.1| Phoxinus eos mitochondrial DNA complete genome

TAGAACAGGCTCCTCTAGGGGGG-TCTAAGGCACCGTCAGGTCCTTTGGGTTTCAAGCTA

ATGCTCGTAGTACCCGGGCGGACGTCTAATTGTAGTTGGACATCTAGGTTTATGGCTGAG

CATAGTGGGGTATCTAA

>M01138:27:000000000-AHTAW:1:1101:10076:10447:dbj|AP009151.1| Phoxinus eos mitochondrial DNA complete genome

TAGAACAGGCTCCTCTAGGGGGG-TTTAAGGCACCGTCAGGTCCTTTGGGTTTCACGCTA

ATGCTCGTAGTACCCGGGCGCACGTCTAATTGTAGTTGGACATCTAGGTTTATGGCTGAG

CATAGTGGGGTATCTAA

>M01138:27:000000000-AHTAW:1:1105:28992:11335:dbj|AP009151.1| Phoxinus eos mitochondrial DNA complete genome

TAGAACAGGCTCCTCTAGGGGGG-TTTAAGGCACCGTCAGGTCCTTTGGGTCTCAAGCTA

ATGCTCGTAGTACCCGGGCGGACGTCTAATTGTAGTTGGCCATCTAGGTTTATGGCTGAG

CATAGTGGGGTATCTAA

>M01138:27:000000000-AHTAW:1:1102:18525:19797:dbj|AP009151.1| Phoxinus eos mitochondrial DNA complete genome

TAGAACAGGCTCCTCTAGGGGGG-TTTAAGGCACCGTCAGGTCCTTTGGGTTTCAAGCTA

ATGCTCGTAGTACCCGGGCGGACGTCTAATTGTAGTTGGACATCTAGGTTTATGACTGGG

CATAGTGGGGTATCTAA

>M01138:27:000000000-AHTAW:1:1102:28462:11695:dbj|AP009151.1| Phoxinus eos mitochondrial DNA complete genome

TAGAACAGGCTCCTCTAGGGGGG-TTTAAGGCACCGTCAGGTCCTTTGGGCTTCAAGCTA

ATGCTCGGAGTACCCGGGCGGACGTCTAATTGTAGTTGGACATCTAGGTTTATGGCTGAG

CATAGTGGGGTATCTAA

>M01138:27:000000000-AHTAW:1:1101:19077:2857:dbj|AP009151.1| Phoxinus eos mitochondrial DNA complete genome

TAGAACAGGCTCCTCTAGGGGGG-TTTAAGGCACCGTCAGGTCCTTTGGGTTTCAAGCCA

ATGCTCGTAGTACCCGGGCGGACGTCTAATTGTAGTTGGGCATCTAGGTTTATGGCTGAG

CATAGTGGGGTATCTAA

>M01138:27:000000000-AHTAW:1:1101:7699:5567:dbj|AP009151.1| Phoxinus eos mitochondrial DNA complete genome

TAGAACAGGCTCCTCTAGGGGGG-TTTAAGGCGCCGTCAGGTCCCTTGGGTTTCAAGCTA

ATGCTCGTAGTACCCGGGCGGACGTCTAATTGTAGTTGGACATCTAGGTTTATGGCTGAG

CATAGTGGGGTATCTAA

>M01138:27:000000000-AHTAW:1:1101:13379:24668:dbj|AP009151.1| Phoxinus eos mitochondrial DNA complete genome

TAGAACAGGCTCCTCTAGGGGGA-TTTAAGGCACCGTCAGGTCCTTTGGGTTTCAAGCTA

ATGCTCGTAGTACCCGGGCGGACGTCTAATTGTAGTTGGACATCTAGGCTTATGGCTGAG

CATAGTGGGGTATCTAA

>M01138:27:000000000-AHTAW:1:1103:13071:3421:dbj|AP009151.1| Phoxinus eos mitochondrial DNA complete genome

TAGAACAGGCTCCTCTAGGGGGG-TTTAAGGCACCGTCAGGCCCTTTGGGTTTCAAGCTA

ATGCTCGTAGTACCCGGGCGGACGTCTAATTGTAGTTGGACATCTAGGTTTATGGCCGAG

CATAGTGGGGTATCTAA

>M01138:27:000000000-AHTAW:1:1101:20431:4289:dbj|AP009151.1| Phoxinus eos mitochondrial DNA complete genome

TAGAACAGGCTCCTCTAGGGGGG-TTTAAGGCACCGTCAGGTCCTTTGGGTTTCAAGCTA

GTGCTCGTAGTACCCGGGCGGGCGTCTAATTGTAGTTGGACATCTAGGTTTATGGCTGAG

CATAGTGGGGTATCTAA

>M01138:27:000000000-AHTAW:1:1101:19632:23484:dbj|AP009151.1| Phoxinus eos mitochondrial DNA complete genome

TAGAACAGGCTCCTCTAGGGGGG-TTTAAGGCACCGTCAGGTCCTTCGGGTTTCAAGCTA

ATGCTCGTAGTACCCGGGCGGACGTCTAATTGTAGTTGGACATCCAGGTTTATGGCTGAG

CATAGTGGGGTATCTAA

>M01138:27:000000000-AHTAW:1:1102:19122:8108:dbj|AP009151.1| Phoxinus eos mitochondrial DNA complete genome

TAGAACAGGCTCCTCTAGGGGGG-TTTAAGGCACCGTCAGGTCCTTTGGGTTTCAAGCTA

ATGCTCGTAGTACCCGGGCGGACGTCTAATTGTAGTTGGACATCTCGGTTTATGGTTGAG

CATAGTGGGGTATCTAA

>M01138:27:000000000-AHTAW:1:1103:19535:11923:dbj|AP009151.1| Phoxinus eos mitochondrial DNA complete genome

TAGAACAGGCTCCTCTAGGGGGG-TTTAAGGCACTGACAGGTCCTTTGGGTTTCAAGCTA

ATGCTCGTAGTACCCGGGCGGACGTCTAATTGTAGTTGGACATCTAGGTTTATGGCTGAG

CATAGTGGGGTATCTAA

>M01138:27:000000000-AHTAW:1:1101:28254:9322:dbj|AP009151.1| Phoxinus eos mitochondrial DNA complete genome

TAGAACAGGCTCCTCTAGGGGGG-TTTAAGGCACCGTCAGGTCCTTTGGGTTTCATGCTA

ATGCTCGTAGTCCCCGGGCGGACGTCTAATTGTAGTTGGACATCTAGGTTTATGGCTGAG

CATAGTGGGGTATCTAA

>M01138:27:000000000-AHTAW:1:1107:26847:12718:dbj|AP009151.1| Phoxinus eos mitochondrial DNA complete genome

TAGAACAGGCTCCTCTAGGGGGG-TTTAAGGCACCGTCAGGTCCTTTGGGTTTCAAGCTA

ATGCTCGTAGTACCCGGGCGGACGTCTAATTGTAGTTGGACATCTAGGTTTAGGGCTGTG

CATAGTGGGGTATCTAA

>M01138:27:000000000-AHTAW:1:1105:20820:18917:dbj|AP009151.1| Phoxinus eos mitochondrial DNA complete genome

TAGAACAGGCTCCTCTAGGGGGG-TTTAAGGCACCGTCAGGTCCTTTGGGTTTCAAGCTA

ATGCTCGCAGTACCCGGGCGGACGTCTAATTGTAGTTGGACATCTAGGTCTATGGCTGAG

CATAGTGGGGTATCTAA

>M01138:27:000000000-AHTAW:1:1103:5382:20040:dbj|AP009151.1| Phoxinus eos mitochondrial DNA complete genome

TAGAACAGGCTCCTCTAGGGGGG-TTCAAGGCACCGTCAGGTCCTTTGGGTTTCAAGCTA

ATGCTCGTAGTACCCGGGCGGACGTCCAATTGTAGTTGGACATCTAGGTTTATGGCTGAG

CATAGTGGGGTATCTAA

>M01138:27:000000000-AHTAW:1:1103:2911:13742:dbj|AP009151.1| Phoxinus eos mitochondrial DNA complete genome

TAGAACAGGCTCCTCTAGGGGGG-TTTAAGGCACCGTCAGTTCCTTTTGGTTTCAAGCTA

ATGCTCGTAGTACCCGGGCGGACGTCTAATTGTAGTTGGACATCTAGGTTTATGGCTGAG

CATAGTGGGGTATCTAA

>M01138:27:000000000-AHTAW:1:1107:15333:2731:dbj|AP009151.1| Phoxinus eos mitochondrial DNA complete genome

TAGAACAGGCTCCTCTAGGGGGG-TTTAAGGCACCGTCAGGTCCTTTGGGTTTCGAGCTA

ATGCTCGTAGTACCCGGGCGGACGTCTAATAGTAGTTGGACATCTAGGTTTATGGCTGAG

CATAGTGGGGTATCTAA

>M01138:27:000000000-AHTAW:1:1103:10637:20872:dbj|AP009151.1| Phoxinus eos mitochondrial DNA complete genome

TAGAACAGGCTCCTCTAGGGGGG-TTTAAGGCATCGTCAGGTCCTTTGGGTTTCAAGCTA

ATGCCCGTAGTACCCGGGCGGACGTCTAATTGTAGTTGGACATCTAGGTTTATGGCTGAG

CATAGTGGGGTATCTAA

>M01138:27:000000000-AHTAW:1:1105:13045:4882:dbj|AP009151.1| Phoxinus eos mitochondrial DNA complete genome

TAGAACAGGCTCCTCTAGGGGGG-CTTAAGGCACCGTCAGGTCCTTTGGGTTTCAAGCTA

ATGCTCGTAGTACCCGGGCGGACGTCTAATTGTAGTTGGACATCAAGGTTTATGGCTGAG

CATAGTGGGGTATCTAA

>M01138:27:000000000-AHTAW:1:1101:8088:3377:dbj|AP009151.1| Phoxinus eos mitochondrial DNA complete genome

TAGAACAGGCTCCTCTAGGGGGG-TTTAAGGCACCGTCAGGTCCTTTAGGTTTCAAGCTA

ATGCTCGTGGTACCCGGGCGGACGTCTAATTGTAGTTGGACATCTAGGTTTATGGCTGAG

CATAGTGGGGTATCTAA

>M01138:27:000000000-AHTAW:1:1107:10469:4487:dbj|AP009151.1| Phoxinus eos mitochondrial DNA complete genome

TAGAACAGGCTCCTCTAGGGGGG-TTTAAGGCACCGTCAGGTCCTTTGGGTTCCAAGCTA

ATGCTCGTAGTACCCGGGCGGACGTCTAATTGTAGTTGGACATCTAGGTTTATGGCTAAG

CATAGTGGGGTATCTAA

>M01138:27:000000000-AHTAW:1:1111:20009:15439:dbj|AP009151.1| Phoxinus eos mitochondrial DNA complete genome

TAGAACAGGCTCCTCTAGGGGGG-TTTAAGGCACCGTCAGGTCCTTTGGGTTTCAAGCTA

ATGCTCGTCGTACCCGGGCGGACGTCTAATCGTAGTTGGACATCTAGGTTTATGGCTGAG

CATAGTGGGGTATCTAA

>M01138:27:000000000-AHTAW:1:1112:24657:7784:dbj|AP009151.1| Phoxinus eos mitochondrial DNA complete genome

TAGAACAGGCTCCTCTAGGGGGG-TTTAAGGCACCGTCAGGTCCTTTGGGTTTCAAGCTA

ATGCTCGTAGTGCCCGGGCGGACGTCTGATTGTAGTTGGACATCTAGGTTTATGGCTGAG

CATAGTGGGGTATCTAA

>M01138:27:000000000-AHTAW:1:1109:6934:14233:dbj|AP009151.1| Phoxinus eos mitochondrial DNA complete genome

TAGAACAGGCTCCTCTAGGGGGG-TTTAAGGTACCGTCAGGTCCTCTGGGTTTCAAGCTA

ATGCTCGTAGTACCCGGGCGGACGTCTAATTGTAGTTGGACATCTAGGTTTATGGCTGAG

CATAGTGGGGTATCTAA

>M01138:27:000000000-AHTAW:1:1102:21894:7921:dbj|AP009151.1| Phoxinus eos mitochondrial DNA complete genome

TAGAACAGGCTCCTCTAGGGGGG-TTTAGGGCACCGTCAGGTCCTTTGGGTTTCAAGCTA

ATGCTCGTAGTACCCGGGCGGACGTCTAATTGTAGTTGGACATCTAGGTTTATGCCTGAG

CATAGTGGGGTATCTAA

>M01138:27:000000000-AHTAW:1:1104:24691:17761:dbj|AP009151.1| Phoxinus eos mitochondrial DNA complete genome

TAGAACAGGCTCCTCTAGAGAGG-TTTAAGGCACCGTCAGGTCCTTTGGGTTTCAAGCTA

ATGCTCGTAGTACCCGGGCGGACGTCTAATTGTAGTTGGACATCTAGGTTTATGGCTGAG

CATAGTGGGGTATCTAA

>M01138:27:000000000-AHTAW:1:1109:5013:21054:dbj|AP009151.1| Phoxinus eos mitochondrial DNA complete genome

TAGAACAGGCTCCTCTAGGGGGG-TTTAAGGCACCGTCAGGTCCTTTGGGTTTCAAGCTA

ATACTCGTAGTACCCAGGCGGACGTCTAATTGTAGTTGGACATCTAGGTTTATGGCTGAG

CATAGTGGGGTATCTAA

>M01138:27:000000000-AHTAW:1:1101:9689:18953:dbj|AP009151.1| Phoxinus eos mitochondrial DNA complete genome

TAGAACAGGCTCCTCTAGGGGGG-TTTAAGACACCGTCAGGTCCTTTGGGTTTCAAGCTA

ATGCTCGTAGTACCCGGGCGGACGTCTAATTGTAGTTGGACAACTAGGTTTATGGCTGAG

CATAGTGGGGTATCTAA

>M01138:27:000000000-AHTAW:1:1107:15792:17894:dbj|AP009151.1| Phoxinus eos mitochondrial DNA complete genome

TAGAACAGGCTCCTCTAGGGGGG-TTTAAGGCACCGTCAGGTCCTTTGGATTTCAAGCTA

ACGCTCGTAGTACCCGGGCGGACGTCTAATTGTAGTTGGACATCTAGGTTTATGGCTGAG

CATAGTGGGGTATCTAA

>M01138:27:000000000-AHTAW:1:1102:3950:14589:dbj|AP009151.1| Phoxinus eos mitochondrial DNA complete genome

TAGAACAGGCTCCTCTAGGGGGG-TTTAAGGCACCGGCAGGTCCTTTGGGTTTCAAGCTA

ATGCTCGTAGTACCCGGGCGGACGTCTAATGGTAGTTGGACATCTAGGTTTATGGCTGAG

CATAGTGGGGTATCTAA

>M01138:27:000000000-AHTAW:1:2101:3213:15750:dbj|AP009151.1| Phoxinus eos mitochondrial DNA complete genome

TAGAACAGGCTCCTCTAGGGGGG-TTTAAGGCACCGTCAGGTCCTTTGGGTTTCAGGCTA

ATGCTCGTAGTACCTGGGCGGACGTCTAATTGTAGTTGGACATCTAGGTTTATGGCTGAG

CATAGTGGGGTATCTAA

>M01138:27:000000000-AHTAW:1:1108:23591:24672:dbj|AP009151.1| Phoxinus eos mitochondrial DNA complete genome

TAGAACAGGCTCCTCTAGGGGGG-TTTAAGGCACCGTCAGGTCCTTTGGGTTTCAAGCTG

ATGCTCGTAGTACCCGGGCGGACGTCTAATTGTAGTTGGACATATAGGTTTATGGCTGAG

CATAGTGGGGTATCTAA

>M01138:27:000000000-AHTAW:1:1102:26159:4447:dbj|AP009151.1| Phoxinus eos mitochondrial DNA complete genome

TAGAACAGGCTCCTCTAGGGGGG-TTTAAGGCACCGTCAGGTCCTTTGGGTTTCAAGCTA

ATGCTCGTAGTACCCGGGCGGACGTCTAATTGTAGTTGGAAATCTAGGTTTCTGGCTGAG

CATAGTGGGGTATCTAA

>M01138:27:000000000-AHTAW:1:1107:9844:20932:dbj|AP009151.1| Phoxinus eos mitochondrial DNA complete genome

TAGAACAGGCTCCTCTAGGGGGG-TTTAAGGCACCGTCTGGTCCTTTGGGTTTCAAGCTA

ATGCTCGTAGTACCCGGGCGGACGTCTAATTGTAGTCGGACATCTAGGTTTATGGCTGAG

CATAGTGGGGTATCTAA

>M01138:27:000000000-AHTAW:1:1101:15353:3708:dbj|AP009151.1| Phoxinus eos mitochondrial DNA complete genome

TAGAACAGGCTCCTCTAGGGGGG-TTTAAGGCACCGTCAGGTCCTTTGGGTTTCAAGCTA

ATGCTCGTAGCACCCGGGCGGACGTGTAATTGTAGTTGGACATCTAGGTTTATGGCTGAG

CATAGTGGGGTATCTAA

>M01138:27:000000000-AHTAW:1:1111:9747:18477:dbj|AP009151.1| Phoxinus eos mitochondrial DNA complete genome

TAGAACAGGCTCCTCTAGGGGGG-TTTAAGGCACCGTCAGGTCCTTTGGGTTTCAAGCTA

ATGCTCGTAGTACCCGGGCGGACGTCTAATTGTGGTTGGACATCTAGGTTTATAGCTGAG

CATAGTGGGGTATCTAA

>M01138:27:000000000-AHTAW:1:1114:12474:2387:dbj|AP009151.1| Phoxinus eos mitochondrial DNA complete genome

TAGAACAGGCTCCTCTAGGGGGG-TTTAAGGCACCGTCAGGTCCTTTGGGTTTCAAGCTA

ATGCTCGTAGTACTCGGGCGGACATCTAATTGTAGTTGGACATCTAGGTTTATGGCTGAG

CATAGTGGGGTATCTAA

>M01138:27:000000000-AHTAW:1:2107:23770:14593:dbj|AP009151.1| Phoxinus eos mitochondrial DNA complete genome

TAGAACAGGCTCCTCTAGGGGGG-TTTAAGGCACCATCAGGTCCTTTGGGTTTCAAGCTA

ATGCTCGTAGTACCCGGGCGGACGTCTAGTTGTAGTTGGACATCTAGGTTTATGGCTGAG

CATAGTGGGGTATCTAA

>M01138:27:000000000-AHTAW:1:1115:13311:16047:dbj|AP009151.1| Phoxinus eos mitochondrial DNA complete genome

TAGAACAGGCTCCTCTAGGGGGG-TTTAAGGCACCGTCAGGTCCTTTGGGTTTCAAGCTA

ATGCTCGTAGTACCCGGGCGGACGTCTAACTGTAGTTGGACATCTAGGTATATGGCTGAG

CATAGTGGGGTATCTAA

>M01138:27:000000000-AHTAW:1:1103:21674:1104:dbj|AP009151.1| Phoxinus eos mitochondrial DNA complete genome

TAGAACAGGCTCCTCTAGGGGGG-TTTAAGGCACCGTCAGGTCCGTTGGGTTTCAAGCTA

ATGCTCGTAGTACCCGGGCGGACGTCTAATTGTAGATGGACATCTAGGTTTATGGCTGAG

CATAGTGGGGTATCTAA

>M01138:27:000000000-AHTAW:1:1103:21961:24412:dbj|AP009151.1| Phoxinus eos mitochondrial DNA complete genome

TAGAACAGGCTCCTCTAGGTGGG-TTTAAGGCACCGTCCGGTCCTTTGGGTTTCAAGCTA

ATGCTCGTAGTACCCGGGCGGACGTCTAATTGTAGTTGGACATCTAGGTTTATGGCTGAG

CATAGTGGGGTATCTAA

>M01138:27:000000000-AHTAW:1:1112:19318:10447:dbj|AP009151.1| Phoxinus eos mitochondrial DNA complete genome

TAGAACAGGCTCCTCTAGGGGGG-TTTAAGGCACCGTCAGGTCCTTTGGGTTTTAAGCTA

ATGCTCGTAGTACGCGGGCGGACGTCTAATTGTAGTTGGACATCTAGGTTTATGGCTGAG

CATAGTGGGGTATCTAA

>M01138:27:000000000-AHTAW:1:1115:7537:10223:dbj|AP009151.1| Phoxinus eos mitochondrial DNA complete genome

TAGAACAGGCTCCTCTAGGAGGG-TTTAAGGCACCGTCAGGTCCTTTGGGTTTCAAGCTA

ATGCTCGTAGTACCCGGGCGGACGTCTAATTGTAGTTGGACATTTAGGTTTATGGCTGAG

CATAGTGGGGTATCTAA

>M01138:27:000000000-AHTAW:1:1109:7804:7139:dbj|AP009151.1| Phoxinus eos mitochondrial DNA complete genome

TAGAACAGGCTCCTCTAGGGGGG-TTTAAGGCACCGTCAGGTCCTTTGGGTTTCAAGCTA

ATGCTCGTAGTACCCGGGCGGACGTCTAATTGTATTTGGTCATCTAGGTTTATGGCTGAG

CATAGTGGGGTATCTAA

>M01138:27:000000000-AHTAW:1:1102:15667:1827:dbj|AP009151.1| Phoxinus eos mitochondrial DNA complete genome

TAGAACAGGCTCCTCTAGGGGGG-TTTAAGGCACCGTCAGGTCCTTTGGGTTTCAAGCTA

ATGCTCGTAGTACCCGGTCGGACGTCTAAGTGTAGTTGGACATCTAGGTTTATGGCTGAG

CATAGTGGGGTATCTAA

>M01138:27:000000000-AHTAW:1:1114:16153:24323:dbj|AP009151.1| Phoxinus eos mitochondrial DNA complete genome

TAGAACAGGCTCCTCTAGGGGGG-GTTAAGGCACCGTTAGGTCCTTTGGGTTTCAAGCTA

ATGCTCGTAGTACCCGGGCGGACGTCTAATTGTAGTTGGACATCTAGGTTTATGGCTGAG

CATAGTGGGGTATCTAA

>M01138:27:000000000-AHTAW:1:1109:8620:14388:dbj|AP009151.1| Phoxinus eos mitochondrial DNA complete genome

TAGAACAGGCTCCTCTAGGGGGG-TTTAAGGCACCGTCAGGTCCTTTGGGTTTCAAGCTA

ATGCTCGTAGTACCCGGGAGGACGTCTAATTGTAGTTGGACATCTAGGTTTATGGATGAG

CATAGTGGGGTATCTAA

>M01138:27:000000000-AHTAW:1:1102:21147:15744:dbj|AP009151.1| Phoxinus eos mitochondrial DNA complete genome

TAGAACAGGCTCCTCTAGGGGGG-TTTATGGCACCGTCAAGTCCTTTGGGTTTCAAGCTA

ATGCTCGTAGTACCCGGGCGGACGTCTAATTGTAGTTGGACATCTAGGTTTATGGCTGAG

CATAGTGGGGTATCTAA

>M01138:27:000000000-AHTAW:1:1105:21541:1827:dbj|AP009151.1| Phoxinus eos mitochondrial DNA complete genome

TAGAACAGGCTCCTCTAGGGGGG-TTTAAGGCACCGTCAGGTCCTTTGGGTTTCAAGCTA

ATGCTCTTAGTACCCGGACGGACGTCTAATTGTAGTTGGACATCTAGGTTTATGGCTGAG

CATAGTGGGGTATCTAA

>M01138:27:000000000-AHTAW:1:1116:26452:16326:dbj|AP009151.1| Phoxinus eos mitochondrial DNA complete genome

TAGAACAGGCTCCTCTAGGGGGG-TTTAAGGCACCGTCAGGTCCTTTGGGTTTCAAGCGA

ATGCTCGTAGTACCCGGGCGGACGTCTAATTGTAGTTGGACATCTAGGTTCATGGCTGAG

CATAGTGGGGTATCTAA

>M01138:27:000000000-AHTAW:1:1118:26999:11893:dbj|AP009151.1| Phoxinus eos mitochondrial DNA complete genome

TAGAACAGGCTCCTCTAGGGGGG-TTTACGGCACCGTCAGGTCCTTTGGGTTTCAAGCTA

ATGCTCGTAGTACCCGGGTGGACGTCTAATTGTAGTTGGACATCTAGGTTTATGGCTGAG

CATAGTGGGGTATCTAA

>M01138:27:000000000-AHTAW:1:1108:25434:20550:dbj|AP009151.1| Phoxinus eos mitochondrial DNA complete genome

TAGAACAGGCTCCTCTAGGGGGG-TTTAAGGCACCGTCAGGTCCTGTGGGTTTCAAGCTA

ATGCTCGTAGTACCCGGGCGGACGTCTAATTGTAGTTGGACATCTAGATTTATGGCTGAG

CATAGTGGGGTATCTAA

>M01138:27:000000000-AHTAW:1:1110:2234:9989:dbj|AP009151.1| Phoxinus eos mitochondrial DNA complete genome

TAGAACAGGCTCCTCTAGGGGGG-TTTAAGGCACCGTCAGGTCCTTTGGGTTTCAAGCTA

ATGCTCGTAGGACCCGGGCGGACGTCTAATTTTAGTTGGACATCTAGGTTTATGGCTGAG

CATAGTGGGGTATCTAA

>M01138:27:000000000-AHTAW:1:1110:14859:20080:dbj|AP009151.1| Phoxinus eos mitochondrial DNA complete genome

TAGAACAGGCTCCTCTAGGGGAG-TTTAAGGCACCGTCAGGTCCTTTGGGTTTCAAGCTA

ATGCTCGTAGTACCCGGGCGGACGTCTAATTGTAGTTGGACATCTAGGGTTATGGCTGAG

CATAGTGGGGTATCTAA

>M01138:27:000000000-AHTAW:1:2108:14585:16654:dbj|AP009151.1| Phoxinus eos mitochondrial DNA complete genome

TAGAACAGGCTCCTCTAGGGGGG-TTTAAGGCACCGTCAGGTCCTTTGGGTTTCAAGCTA

ATGCTCGTAGTACCCGGGCGTACGTCTAATTGTAGTTGGACATCTAGGTTTGTGGCTGAG

CATAGTGGGGTATCTAA

>M01138:27:000000000-AHTAW:1:1106:16965:24642:dbj|AP009151.1| Phoxinus eos mitochondrial DNA complete genome

TAGAACAGGCTCCTCTAGGGGGG-TTTAAGGCACCGTCAGGTCCTTTGGGTTGCAAGCTA

ATGCTCGTAGTACCCGGGCGAACGTCTAATTGTAGTTGGACATCTAGGTTTATGGCTGAG

CATAGTGGGGTATCTAA

>M01138:27:000000000-AHTAW:1:2112:5724:5650:dbj|AP009151.1| Phoxinus eos mitochondrial DNA complete genome

TAGAACAGGCTCCTCTAGGGGGG-TTTAAGGCACCGTCAGGTCCTTTGGGTTTCAAGCTA

ATGCTCGTAGTACCCGTGCGGACGTCTAATTGTAGTTGGACATCTTGGTTTATGGCTGAG

CATAGTGGGGTATCTAA

>M01138:27:000000000-AHTAW:1:1103:27459:18315:dbj|AP009151.1| Phoxinus eos mitochondrial DNA complete genome

TAGAACAGGCTCCTCTAGGGGGG-TTTAAGGCACCGTCAGGTCCTTTGGGCTTCACGCTA

ATGCTCGTAGTACCCGGGCGGACGTCAAATTGTAGTTGGACATCTAGGTTTATGGCTGAG

CATAGTGGGGTATCTAA

>M01138:27:000000000-AHTAW:1:1107:24986:3809:dbj|AP009151.1| Phoxinus eos mitochondrial DNA complete genome

TAGAACAGGCTCCTCTAGGGGGG-TTTAAGGCACCGTCAGGTCCTTTGGGTTTCACGCTA

ATGCTCGTCGTACCCGGGTGGACGTCTAATTGTAGTTGGACATCTAGGTTTATGGCTGAG

CATAGTGGGGTATCTAA

>M01138:27:000000000-AHTAW:1:1114:8713:2641:dbj|AP009151.1| Phoxinus eos mitochondrial DNA complete genome

TAGAACAGGCTCCTCTAGGGGGG-TTTAAGGCACCGTCAGGTCCTTTGGGTTTGAAGCTA

ATGCTCGTAGTACCCGGGCGGACGTCTAATTGTAGTTGGACATGTAGGTTTATGGCTGAG

CATAGTGGGGTATCTAA

>M01138:27:000000000-AHTAW:1:1117:11359:15512:dbj|AP009151.1| Phoxinus eos mitochondrial DNA complete genome

TAGAACAGGCTCCTCTAGGGGGG-TTTAAAGCACCGTCAGGTCCTTTGGGTTTCAAGCTA

ATGCTCGTAGTACCCGGGCCGACGTCTAATTGTAGTTGGACATCTAGGTTTATGGCTGAG

CATAGTGGGGTATCTAA

>M01138:27:000000000-AHTAW:1:1111:14912:19332:dbj|AP009151.1| Phoxinus eos mitochondrial DNA complete genome

TAGAACAGGCTCCTCTAGGGGGG-TTTAAGGCACCGTCAGGTCCTTTGGGTTTCAAGCTA

ATGCGCGTAGTACCCGAGCGGACGTCTAATTGTAGTTGGACATCTAGGTTTATGGCTGAG

CATAGTGGGGTATCTAA

>M01138:27:000000000-AHTAW:1:1113:16488:18192:dbj|AP009151.1| Phoxinus eos mitochondrial DNA complete genome

TAGAACAGGCTCCTCTAGGGGGG-TTTAAGGCACCGTCAGGTCATTTGGGTTTCAAGCTA

ATGCTTGTAGTACCCGGGCGGACGTCTAATTGTAGTTGGACATCTAGGTTTATGGCTGAG

CATAGTGGGGTATCTAA

>M01138:27:000000000-AHTAW:1:2101:18521:17958:dbj|AP009151.1| Phoxinus eos mitochondrial DNA complete genome

TAGAACAGGCTCCTCTAGGGGGG-TTTAAGGCACCGTCAGGTCCTATGGGTTTCAAGCTA

ATGCTCGTAGTACCCGGGCGGACGTCTAATTGTAGTTGGAGATCTAGGTTTATGGCTGAG

CATAGTGGGGTATCTAA

>M01138:27:000000000-AHTAW:1:1105:12617:23308:dbj|AP009151.1| Phoxinus eos mitochondrial DNA complete genome

TAGAACAGGCTCCTCTAGGGGGG-TTTAAGGCACCGTCAGGTCCTTTGGGTTTCAAGCTG

ATGCTCGTAGTACCCGGGCGGACGTCTAATTGTTGTTGGCCATCTAGGTTTATGGCTGAG

CATAGTGGGGTATCTAA

>M01138:27:000000000-AHTAW:1:1106:28929:10858:dbj|AP009151.1| Phoxinus eos mitochondrial DNA complete genome

TAGAACAGGCTCCTCTAGGGGGG-TTTAAGGCACCGTCAGGTCCTTTGGGTTTCAAGCTA

ATGCTCGTAGTACCAGGGCGGACGTCTAATTGTTGTTGGACATCTAGGTTTATGGCTGAG

CATAGTGGGGTATCTAA

>M01138:27:000000000-AHTAW:1:1102:3607:15984:dbj|AP009151.1| Phoxinus eos mitochondrial DNA complete genome

TAGAACAGGCTCCTCTAGGGGGG-TTTAAGGCACCGTCAGGTCCTTTGGGTTTCGAGCTA

ATGCTCGTAGTACCCGGGCGGACGTCTAATTGTAGTTGGACATCTAGGTCCATGGCTGAG

CATAGTGGGGTATCTAA

>M01138:27:000000000-AHTAW:1:1106:9658:5706:dbj|AP009151.1| Phoxinus eos mitochondrial DNA complete genome

TAGAACAGGCTCCTCTAGGGGGG-TTTAAGGCACCGTCAGGTCCTTTGGGTTTCAAGCTA

ATGCTCGTAGTACCCGGGCGGACGTCTGATTGTAGTTGGACATCTTGGTTTAGGGCTGAG

CATAGTGGGGTATCTAA

>M01138:27:000000000-AHTAW:1:1108:5824:5193:dbj|AP009151.1| Phoxinus eos mitochondrial DNA complete genome

TAGAACAGGCTCCTCTAGGGGGG-TTTAAGGCACCGTCAGGTCCTTTGGGTTTCAAGCTA

ATGCTCGTAGTCCCCGGGCGGACGTCTAATTGTAGTTGGCCATCTAGGTTTTTGGCTGAG

CATAGTGGGGTATCTAA

>M01138:27:000000000-AHTAW:1:1115:28885:17706:dbj|AP009151.1| Phoxinus eos mitochondrial DNA complete genome

TAGAACAGGCTCCTCTAGGGGGG-TTTAAGGCACCGTCAGGTCCTTTGGGTGTCAAGCTA

ATGCTCGTAGTATCCGGGAGGACGTCTAATTGTAGTTGGACATCTAGGTTTATGGCTGAG

CATAGTGGGGTATCTAA

>M01138:27:000000000-AHTAW:1:1118:8540:3083:dbj|AP009151.1| Phoxinus eos mitochondrial DNA complete genome

TAGAACAGGCTCCTCTAGGGGGGGTTTAAGGCACCGTCAGGTCCTTTGGGTTTCAAGCTA

ATGTTCGT-GTACCCGGGCGGACGTCTAATTGTAGTTGGACATCTAGGTTTATGGCTGAG

CATAGTGGGGTATCTAA

>M01138:27:000000000-AHTAW:1:1101:28400:14180:dbj|AP009151.1| Phoxinus eos mitochondrial DNA complete genome

TAGAACAGGCTCCTCTAGGGGGG-TTTAAGGCACCGTCAGGTCCTTTGGGTTTCGAGCTA

ATGCTCGTAGTACCCGGGCGGACGTCTAATTTTAGTTGGCCATCTAGGTTTATGGCTGAG

CATAGTGGGGTATCTAA

>M01138:27:000000000-AHTAW:1:1101:17221:14163:dbj|AP009151.1| Phoxinus eos mitochondrial DNA complete genome

TAGAACAGGCTCCTCTAGGGGGG-TTTAAGGCACCGTCAGGTCCTTTGGGTTTCAAGCTA

ATGCTCGTAGTACCCGGGCGGGCGTCTAATTGTGGTTGGACATCTCGGTTTATGGCTGAG

CATAGTGGGGTATCTAA

>M01138:27:000000000-AHTAW:1:1109:23014:19264:dbj|AP009151.1| Phoxinus eos mitochondrial DNA complete genome

TAGAACAGGCTCCTCTAGGGGGG-TTTAAGGCACCGTCAGGTCCTTTGGGTTTCACGCTA

ATGCTCGTAGTACCCGGGCGGACGTCTAATTGTAGTTGGCCATCTAGGTTCATGGCTGAG

CATAGTGGGGTATCTAA

>M01138:27:000000000-AHTAW:1:1110:21161:13616:dbj|AP009151.1| Phoxinus eos mitochondrial DNA complete genome

TAGAACAGGCTCCTCTAGGGGGG-TCTAAGGCACCGTCAGGTCCTTTGGATTTCACGCTA

ATGCTCGTAGTACCCGGGCGGACGTCTAATCGTAGTTGGACATCTAGGTTTATGGCTGAG

CATAGTGGGGTATCTAA

>M01138:27:000000000-AHTAW:1:1111:28131:9589:dbj|AP009151.1| Phoxinus eos mitochondrial DNA complete genome

TAGAACAGGCTCCTCTAGGGGGG-TTTAAGGCACCGGCAGGTCCTTTGGGTTTCAAGCTA

ATGCTCGTAGTACCCGGGCGGACGTCTGATTGTAGTTGGCCATCTAGGTTTATGGCTGAG

CATAGTGGGGTATCTAA

>M01138:27:000000000-AHTAW:1:1113:9655:21992:dbj|AP009151.1| Phoxinus eos mitochondrial DNA complete genome

TAGAACAGGCTCCTCTAGGGGGG-TTTAAGGCACCGTCAGGTCCTTTGGGTTTCACGCTA

ATGCTCGTAGTACCCGGGCGGACGTCTAATTGTAGTCGGACATCTCGGTTTATGGCTGAG

CATAGTGGGGTATCTAA

>M01138:27:000000000-AHTAW:1:1119:7733:7927:dbj|AP009151.1| Phoxinus eos mitochondrial DNA complete genome

TAGAACAGGCTCCTCTAGGGGGG-TTTAAGGCACCGTCAGGGCCTTTGGGGTTCAAGCTA

ATGCTCGTAGTACCCGGGCGGACGTCTAATTGTAGTTGGACATCTAGGTTTATGGCTGAG

CATAGTGGGGTATCTAA

>M01138:27:000000000-AHTAW:1:2101:8713:16391:dbj|AP009151.1| Phoxinus eos mitochondrial DNA complete genome

TAGAACAGGCTCCTCTAGGGGGG-TTTAAGGCACCGTCAGGTCCTTTGGGTTTCAAGCTC

ATGCTCGTAGTAACCGGGCGGACGTCTAATTGTAGTTGGACATCTAGGTTTATGGCTGAG

CATAGTGGGGTATCTAA

>M01138:27:000000000-AHTAW:1:2113:6084:23294:dbj|AP009151.1| Phoxinus eos mitochondrial DNA complete genome

TAGAACAGGCTCCTCTAGGGGGG-TTTAAGGCACCGTCAGGTCCTTTGGGTTTCAAGCTA

ATGCTCGTAGTACCCGGGCGGCCGTCTAATTGTAGTTGGACATCTAGGTTTATGGCAGAG

CATAGTGGGGTATCTAA

>M01138:27:000000000-AHTAW:1:2115:10943:20468:dbj|AP009151.1| Phoxinus eos mitochondrial DNA complete genome

TAGAACAGGCTCCTCTAGGGGGG-TTTAAGGCACCGTCAGGTCCTTTGGGTTTCTAGCTA

ATGCTCGTAGTACCCGGGCGGACGTCTAATTGCAGTTGGACATCTAGGTTTATGGCTGAG

CATAGTGGGGTATCTAA

>M01138:27:000000000-AHTAW:1:1107:20707:16043:dbj|AP009151.1| Phoxinus eos mitochondrial DNA complete genome

TAGAACAGGCTCCTCTAGGGGGG-TTTAAGGCACCGTCAGGTCCTTTGGGTTTTAAACTA

ATGCTCGTAGTACCCGGGCGGACGTCTAATTGTAGTTGGACATCTAGGTTTATGGCTAGG

CATAGTGGGGTATCTAA

>M01138:27:000000000-AHTAW:1:1110:8794:11555:dbj|AP009151.1| Phoxinus eos mitochondrial DNA complete genome

TAGAACAGGCTCCTCTAGGGGGG-TTTAAGGCACCGTCGGGTCCTTTGGGTTTCAAGCTA

CTGATCGTAGTACCCGGGCGGACGTCTAATTGTAGTTGGACATCTAGGTTTATGGCTGAG

CATAGTGGGGTATCTAA

>M01138:27:000000000-AHTAW:1:1112:24311:21496:dbj|AP009151.1| Phoxinus eos mitochondrial DNA complete genome

TAGAACAGGCTCCTCTAGGGGGG-TCTAAGGCACCGTCAGGTCCTTTGGGTTTCAAGCTA

ATGCTCGTAGGACCCGGGCGGACGTCTAAGTGTAGTTGGACATCTAGGTTTATTGCTGAG

CATAGTGGGGTATCTAA

>M01138:27:000000000-AHTAW:1:1114:15297:4448:dbj|AP009151.1| Phoxinus eos mitochondrial DNA complete genome

TAGAACAGGCTCCTCTAGGGGGG-TTTATGGCGCCGTCAGGTCCTTTGGGTTTCACGCTA

ATGCTCGTAGTACCCGGGCGGACGTCTAATTGTAGTTGGACATCTAGGTTTATGGCTGAG

CATAGTGGGGTATCTAA

>M01138:27:000000000-AHTAW:1:1116:4868:19444:dbj|AP009151.1| Phoxinus eos mitochondrial DNA complete genome

TAGAACAGGCTCCTCTAGGTGGG-TTTAAGGCACCGTCAGGTCCTTTGGGTTTCAAGCTA

ATGCTCGTAGTACCCGGGCGGACGTCTAATTGTAGTTGGACATCTAGATTTATGGCTGTG

CATAGTGGGGTATCTAA

>M01138:27:000000000-AHTAW:1:1118:4399:12078:dbj|AP009151.1| Phoxinus eos mitochondrial DNA complete genome

TAGAACAGGCTCCTCTAGGGGGG-TTTAAGGCACCGTCAGCTCCTTTGGGTTTCAAGCTA

ATGCTCGTAGTACCCGGGCGGACGTCTAATTGTACTTGGACATCTAGGTTTATGGCTGAG

CATAGTGGGGTATCTAA

>M01138:27:000000000-AHTAW:1:2101:24352:3473:dbj|AP009151.1| Phoxinus eos mitochondrial DNA complete genome

TAGAACAGGCTCCTCTAGGGGGG-TCTAAGGCACCGTCGGGTCCTTTGGGTTTCAAGCTA

ATGCTCGTAGTACCCGGGCGGACGTCTAATTGTAGTTGGCCATCCAGGTTTATGGCTGAG

CATAGTGGGGTATCTAA

>M01138:27:000000000-AHTAW:1:2107:25023:10393:dbj|AP009151.1| Phoxinus eos mitochondrial DNA complete genome

TAGAACAGGCTCCTCTAGGGGGG-TTTAAGGCACAGTCAGGTCCTTTGGGTATCAAGCTA

ATGCTCGTAGTACCCGGGCGGACGTCTAATTGTAGTTGGACATCTAGGTTTATGGCTGAG

CATAGTGGGGTATCTAA

>M01138:27:000000000-AHTAW:1:2118:15700:24736:dbj|AP009151.1| Phoxinus eos mitochondrial DNA complete genome

TAGAACAGGCTCCTCTAGGGGGG-TTTAAGGCACCGTCAGGTCCGTTGGGCTTCAAGCTA

ATGCTCGTAGTACCCGGGCGGACGTCTAATTGTAGTTGGCCATCTAGGTTTATGGCTGAG

CATAGTGGGGTATCTAA

>M01138:27:000000000-AHTAW:1:1102:25289:22152:dbj|AP009151.1| Phoxinus eos mitochondrial DNA complete genome

TAGAACAGGCTCCTCTAGGGGGG-TTTAAGGCACCGTCAGGTCCTTTGGATTTCACGCTA

ATGCTCGTCGTACCCGGGCGGACGTCTAATTGTAGTTGGCCATCTAGGTTTATGGCTGAG

CATAGTGGGGTATCTAA

>M01138:27:000000000-AHTAW:1:1102:2883:18395:dbj|AP009151.1| Phoxinus eos mitochondrial DNA complete genome

TAGAACAGGCTCCTCTAGGGGGG-TTTAAGGCACCGCCAGGTCCTTTGGGTTTCAAGCTA

ATGCTCGTCGTACCCGGGCGTACGTCTAATTGTAGTTGGACATCTAGGTTTATGGCTGAG

CATAGTGGGGTATCTAA

>M01138:27:000000000-AHTAW:1:1102:5255:20970:dbj|AP009151.1| Phoxinus eos mitochondrial DNA complete genome

TAGAACAGGCTCCTCTAGGGGGG-TTTAAGGCACCGTCAGGTCCTTTGGGTTTCAAGCTA

ATGCTCGTAGTACCCGGGAGGACGTCTAAGTGGAGTTGGACATCTAGGTTTATGGCTGAG

CATAGTGGGGTATCTAA

>M01138:27:000000000-AHTAW:1:1103:27545:16018:dbj|AP009151.1| Phoxinus eos mitochondrial DNA complete genome

TAGAACAGGCTCCTCTAGGGGGG-TTTAAGGCAACGTCAGGTCCTTTGGGTTTCAAGCTA

ATGCTCGTAGTACCCGGGAGGACGTCTAATGGTAGTTGGAAATCTAGGTTTATGGCTGAG

CATAGTGGGGTATCTAA

>M01138:27:000000000-AHTAW:1:1104:26806:14134:dbj|AP009151.1| Phoxinus eos mitochondrial DNA complete genome

TAGAACAGGCTCCTCTAGGGGGG-TCTAAGGCACCGTCAGGTCCTTTGGGTTTCAAGCTA

ATGCTCGTAGTACCCGGGCGGACGTCAAATTGTAGTTGGCCATCTAGGTTTATGGCTGTG

CATAGTGGGGTATCTAA

>M01138:27:000000000-AHTAW:1:1106:27010:19906:dbj|AP009151.1| Phoxinus eos mitochondrial DNA complete genome

TAGAACAGGCTCCTCTAGGGGGG-TTTAAGGCACCGCCAGGTCCTTTGGGTTTCAAGCTA

ATGCTCGTAGTACCCGGGCGGACGTCTAATTGTAGTTGGAAACCTAGGTTTATGGCTGAG

CATAGTGGGGTATCTAA

>M01138:27:000000000-AHTAW:1:1107:19490:11511:dbj|AP009151.1| Phoxinus eos mitochondrial DNA complete genome

TAGAACAGGCTCCTCTAGGGGGG-TCTAAGGCACCGTCAGGTCCTTTGGGTTTAAAGCTA

ATGCTCGTAGTACCCAGGCGGACGTCTAATTGTAGTTGGACATCTAGGTTTATGGATGAG

CATAGTGGGGTATCTAA

>M01138:27:000000000-AHTAW:1:1108:24501:9414:dbj|AP009151.1| Phoxinus eos mitochondrial DNA complete genome

TAGAACAGGCTCCTCTAGGGGGG-TTTAAGGCACCGTCAGGTCCTTTGGGTTTCACGCTA

ATGCTCGTAGTACCCGGGCGGCCGTCTAATTGTAGTTGGACATCTTGGTTTATGGCTGAG

CATAGTGGGGTATCTAA

>M01138:27:000000000-AHTAW:1:1109:10321:5947:dbj|AP009151.1| Phoxinus eos mitochondrial DNA complete genome

TAGAACAGGCTCCTCTAGGGGGG-TTTAAGGCCCCGTCAGGTCCTTTGGGTTTCACGCTA

ATGCTCGTCGTACCCGGGCGGACGTCTAATTGTAGTTGGACATCTCGGTTTATGGCTGAG

CATAGTGGGGTATCTAA

>M01138:27:000000000-AHTAW:1:1110:28963:9768:dbj|AP009151.1| Phoxinus eos mitochondrial DNA complete genome

TAGAACAGGCTCCTCTAGGGGGG-TCTAAGGCACCGTCAGGTCCTTTGGGTTTCAAGCTA

ATGCTCGTCGTACCCGGGCGGACGTCTAATTGTAGTTGGCCATCTAGGTTTATGGATGAG

CATAGTGGGGTATCTAA

>M01138:27:000000000-AHTAW:1:1110:5392:15413:dbj|AP009151.1| Phoxinus eos mitochondrial DNA complete genome

TAGAACAGGCTCCTCTAGGGGAG-TCTAAGGCACCGTCAGGTCCTTTGGGTTTCAGGCTA

ATGCTCGTAGTACCCGGGCGGACGTCTAATTGTAGTTGGACATCTAGGTTTATGGCCGAG

CATAGTGGGGTATCTAA

>M01138:27:000000000-AHTAW:1:1111:27978:17203:dbj|AP009151.1| Phoxinus eos mitochondrial DNA complete genome

TAGAACAGGCTCCTCTAGGGGGG-TTTAAGGCACCGTCAGGTCCTTTGGGTTTCAAGCTA

ATGCTCGGAGTACCCGGGCGGAAGTCTAATTGTAGTTGGCCATCTAGGTTTATGGCTGAG

CATAGTGGGGTATCTAA

>M01138:27:000000000-AHTAW:1:1113:20370:11820:dbj|AP009151.1| Phoxinus eos mitochondrial DNA complete genome

TAGAACAGGCTCCTCTAGGGGGG-TTTAAGGCACAGTCAGGACCTTTGGGTTTCAAGCTA

ATGCTCGTAGTACCCGGGCGGACGTCTAATTGTAGTTGGACATTTAGGTTTATGGCTGAG

CATAGTGGGGTATCTAA

>M01138:27:000000000-AHTAW:1:1116:11692:2188:dbj|AP009151.1| Phoxinus eos mitochondrial DNA complete genome

TAGAACAGGCTCCTCTAGGGGGG-TTTAAGGCACCGTCAGGTCCTTTGGGTTTTAAGCTA

ATGCTCGTAGTACCCGGGCGGACGTCTATTTGTAGTTGGACATTTAGGTTTATGGCTGAG

CATAGTGGGGTATCTAA

>M01138:27:000000000-AHTAW:1:1116:24868:15737:dbj|AP009151.1| Phoxinus eos mitochondrial DNA complete genome

TAGAACAGGCTCCTCTAGGGGGG-TTTAAGGCACCGTCAGGTCCTTTGGGTTTCAAGCTA

ATGCTCGTAGTACCCGGGCGGACGTCTATTTGTAGTTGGACACGTAGGTTTATGGCTGAG

CATAGTGGGGTATCTAA

>M01138:27:000000000-AHTAW:1:1116:25633:16773:dbj|AP009151.1| Phoxinus eos mitochondrial DNA complete genome

TAGAACAGGCTCCTCTAGGGGGA-TTTAGGGCACCGTCAGGTCCTTTGGGCTTCAAGCTA

ATGCTCGTAGTACCCGGGCGGACGTCTAATTGTAGTTGGACATCTAGGTTTATGGCTGAG

CATAGTGGGGTATCTAA

>M01138:27:000000000-AHTAW:1:1116:8006:3668:dbj|AP009151.1| Phoxinus eos mitochondrial DNA complete genome

TAGAACAGGCTCCTCTAGGGGGG-TCTAAGGCACCGTCAGGTCCTCTGGGGTTCAAGCTA

ATGCTCGTAGTACCCGGGCGGACGTCTAATTGTCGTTGGACATCTAGGTTTATGGCTGAG

CATAGTGGGGTATCTAA

>M01138:27:000000000-AHTAW:1:1117:16726:21590:dbj|AP009151.1| Phoxinus eos mitochondrial DNA complete genome

TAGAACAGGCTCCTCTAGGGGGG-TTTAAGGCACCGTCAGGTCCTCTGGGTTTCAAGCTC

ATGCTCGTAGTACCCGGGCGGACGTCTAATTGTAGTTGGACATCTCGGTTTATGGCTGAG

CATAGTGGGGTATCTAA

>M01138:27:000000000-AHTAW:1:1117:27977:8455:dbj|AP009151.1| Phoxinus eos mitochondrial DNA complete genome

TAGAACAGGCTCCTCTAGGGGGG-TATAAGGCACCGTCAGGTCCTTTGGGTTTTAAGCTA

ATGCTCGTAGTACCCGGGCGGACGTCTAATTGTAGTTGGCCATCTAGGTTTATGGCTGAG

CATAGTGGGGTATCTAA

>M01138:27:000000000-AHTAW:1:2101:5731:21503:dbj|AP009151.1| Phoxinus eos mitochondrial DNA complete genome

TAGAACAGGCTCCTCTAGGGGGG-TTTAAGGCAACGTCAGGTCCTTTGGGTTTAAAGCTA

ATGCTCGTAGTACCCGGGCGGACGTCTAATTGTAGTTGGACATCTAGGTTTATGGCTGAG

CATAGTGGGGTATCTAA

>M01138:27:000000000-AHTAW:1:2103:11227:23175:dbj|AP009151.1| Phoxinus eos mitochondrial DNA complete genome

TAGAACAGGCTCCTCTAGGGGGG-TTTAAGGCACCGTCAGGTCCTTTGGGTTTCAAGCTA

ATGCTCGTAGTACCCGGGCGGACGTCTAATTGTAGTGGGAAATCTCGGTTTATGGCTGAG

CATAGTGGGGTATCTAA

>M01138:27:000000000-AHTAW:1:2103:7391:11336:dbj|AP009151.1| Phoxinus eos mitochondrial DNA complete genome

TAGAACAGGCTCCTCTAGGGGGG-TCTAAGGCACCGTCAGGTCCTTTGGGCTTCAAGCTA

ATGCTCGTCGTACCCGGGCGGACGTCTAATTGTAGTTGGACATCTTGGTTTATGGCTGAG

CATAGTGGGGTATCTAA

>M01138:27:000000000-AHTAW:1:2106:23734:1742:dbj|AP009151.1| Phoxinus eos mitochondrial DNA complete genome

TAGAACAGGCTCCTCTAGGGGGG-TTTAAGGCACCGGCAGATCCTTTGGGTTTCAAGCTA

ATGCTCGTAGTACCCGGGCGGACGACTAATTGTAGTTGGACATCTAGGTTTATGGCTGAG

CATAGTGGGGTATCTAA

>M01138:27:000000000-AHTAW:1:2107:26014:5320:dbj|AP009151.1| Phoxinus eos mitochondrial DNA complete genome

TAGAACAGGCTCCTCTAGGGGGG-TTTAAGGCACCGGCAGGTCCTTTGGGTTTCAAGCTA

AGGCTCGTAGTACCCGGGCGGACGTCTAATTGTAGTTGGACATCTAGGCTTATGGCTGAG

CATAGTGGGGTATCTAA

>M01138:27:000000000-AHTAW:1:2107:8820:5953:dbj|AP009151.1| Phoxinus eos mitochondrial DNA complete genome

TAGAACAGGCTCCTCTAGGGGGG-TTTAAGGCACCGTCAGGTCCTTTGGGTTTCAGGCTA

CTGCTCGTAGTACCCGGGCGGACGTCTAATTGTAGTTGGACATCTCGGTTTATGGCTGAG

CATAGTGGGGTATCTAA

>M01138:27:000000000-AHTAW:1:2110:15705:10409:dbj|AP009151.1| Phoxinus eos mitochondrial DNA complete genome

TAGAACAGGCTCCTCTAGGGGGG-TTTAAGGCACCGTCAGGTCCTTTGGGTGTCAAGCTA

ATGCTCGTAGTACCCGGGCGGACGTCTAATTGTAGTCGGACATCTAGGTTTATTGCTGAG

CATAGTGGGGTATCTAA

>M01138:27:000000000-AHTAW:1:2113:18789:11257:dbj|AP009151.1| Phoxinus eos mitochondrial DNA complete genome

TAGAACAGGCTCCTCTAGGGGGG-TTTAAGGCACCGTCGGTTCCTTTGGGTCTCAAGCTA

ATGCTCGTAGTACCCGGGCGGACGTCTAATTGTAGTTGGACATCTAGGTTTATGGCTGAG

CATAGTGGGGTATCTAA

>M01138:27:000000000-AHTAW:1:2113:21291:3509:dbj|AP009151.1| Phoxinus eos mitochondrial DNA complete genome

TAGAACAGGCTCCTCTAGGGGGG-TCTAAGGCACCGGCAGGCCCTTTGGGTTTCAAGCTA

ATGCTCGTAGTACCCGGGCGGACGTCTAATTGTAGTGGGACATCTAGGTTTATGGCTGAG

CATAGTGGGGTATCTAA

>M01138:27:000000000-AHTAW:1:2114:4846:18971:dbj|AP009151.1| Phoxinus eos mitochondrial DNA complete genome

TAGAACAGGCTCCTCTAGGGGGG-TTTAAGGCACCGTCAGGTCCTTTGGGTTTCAAGCTA

ATGCTCGTAGTACCCGGGCGGACGTCTAATTGTAGTATGACATCTAGGTTTATGGCTGAG

CATAGTGGGGTATCTAA

>M01138:27:000000000-AHTAW:1:2115:18040:5884:dbj|AP009151.1| Phoxinus eos mitochondrial DNA complete genome

TAGAACAGGCTCCTCTAGGGGGG-TTTAAGGGACCGTCAGGTCCTTTGGGTTTCAAGCTA

ATGCTCGTAGTACCCGGGCGGACGTCAAATTGTAGTTGGACATCTAGGTTTATGGCTGAG

CATAGTGGGGTATCTAA

>M01138:27:000000000-AHTAW:1:1111:11123:8079:dbj|AP009151.1| Phoxinus eos mitochondrial DNA complete genome

TAGAACAGGCTCCTCTAGGGGGG-TTTAAGGCACCGTCAGGT-CTTTGGGTTTCAAGCTA

ATGCTCGTAGTACCCGGGCGGACGTCTTATTGTAGTTGGACATCTAGGTTTATGGCTGAG

CATAGTGGGGTATCTAA

>M01138:27:000000000-AHTAW:1:1116:12256:21032:dbj|AP009151.1| Phoxinus eos mitochondrial DNA complete genome

TAGAACAGGCTCCTCTAGGGGG--TTTAAGGCACCGTCAGGTCCTTTGGGTTTCAAACTA

ATGCTCGTAGTACCCGGGCGGACGTCTAATTGTAGTTGGACATCTAGGTTCATGGCTGAG

CATAGTGGGGTATCTAA

>M01138:27:000000000-AHTAW:1:2119:13983:12477:dbj|AP009151.1| Phoxinus eos mitochondrial DNA complete genome

TAGAACAGGCTCCTCTAGGGGGG-TT-AAGGGACCGTCAGGTCCTTTGGGTTTCAAGCTA

ATGCTCGTAGTACCCGGGCGGACGTTTAATTGTAGTTGGACATCTAGGTTTATGGCTGAG

CATAGTGGGGTATCTAA

>M01138:27:000000000-AHTAW:1:2119:21459:2202:dbj|AP009151.1| Phoxinus eos mitochondrial DNA complete genome

TAGAACAGGCTCCTCTAGGGGGG-TTTAAGGCACCGTCAGGTCCTTTGGGTTTCAAGCTA

ATGCTCGTAGTACCCGGGCGGACGTCTAATTGTAGTTGGACAT--AGGTTTATGGCTGAG

CATAGTGGGGTATCTAA

>M01138:27:000000000-AHTAW:1:2108:26874:7729:dbj|AP009151.1| Phoxinus eos mitochondrial DNA complete genome

TAGAACAGGCTCCTCTAGGGGG--TTTAAGGCACGGTCAGGTCCTTTGGGTT-CAAGCTA

ATGCTCGTAGTACCCGGGCGGACGTCTAATTGTAGTTGGACATCTAGGTTTATGGCTGAG

CATAGTGGGGTATCTAA

>M01138:27:000000000-AHTAW:1:1106:19587:1524:dbj|AP012081.1| Margariscus margarita mitochondrial DNA almost complete genome except for D-loop

TAGAACAGGCTCCTCTAGGGGGGGTCTAAGGCACCGTCAGGTCCTTTGGGTTTCAAGCTA

ATGCTCGTAGTACCCGGGCGGACGTCTAATTGTAGTTGGACGTCTAGGTTTATGGATGAG

CATAGTGGGGTATCTAA

>M01138:27:000000000-AHTAW:1:1116:3741:10871:dbj|AP012081.1| Margariscus margarita mitochondrial DNA almost complete genome except for D-loop

TAGAACAGGCTCCTCTAGGGGGGGTCTAAGGCACCGTCAGGACCTTTGGGTTTCAAGCTA

ATGCTCGTAGTACCCGGGTGGACGTCTAATTGTAGTTGGACGTCTAGGTTTATGGCTGAG

CATAGTGGGGTATCTAA

>M01138:27:000000000-AHTAW:1:1101:14462:21806:dbj|AP012081.1| Margariscus margarita mitochondrial DNA almost complete genome except for D-loop

TAGAACAGGCTCCTCTAGGGGAG-TCTAAGGCACCGTCAGGTCCTTTGGGTTTCAAGCTA

ATGCTCGTAGTACCCGGGCGGATGTCTAATTGTAGTTGGACGTCTAGGTTTATGGCTGAG

CATAGTGGGGTATCTAA

>M01138:27:000000000-AHTAW:1:1101:11979:15631:dbj|AP012081.1| Margariscus margarita mitochondrial DNA almost complete genome except for D-loop

TAGAACAGGCTCCTCTAGGGAGG-TCTAAGGCACCGTCAGGTCCTTTGGGTTTCAAGCTA

ATGCTCGTAGTACCCGGGCGGACGTCTAATTGTAGTTGGACGTCTAGGCTTATGGCTGAG

CATAGTGGGGTATCTAA

>M01138:27:000000000-AHTAW:1:1101:19787:7283:dbj|AP012081.1| Margariscus margarita mitochondrial DNA almost complete genome except for D-loop

TAGAACAGGCTCCTCTAGGGGGG-TCTAAGGCACCGTCAGGTCCTTTGGGTTTCACGCTA

ATGCTCGTAGTACCCGGGCGGACGTCTAATTGTAGTTGGACGTCTAGGTCTATGGCTGAG

CATAGTGGGGTATCTAA

>M01138:27:000000000-AHTAW:1:1101:25036:17092:dbj|AP012081.1| Margariscus margarita mitochondrial DNA almost complete genome except for D-loop

TAGAACAGGCTCCTCTAGGGGGG-TCTAAGGCACCGTCAGGTCCTTTGGGCTTCAAGCTA

ATGCTCGTAGTACCCGGGCGGACGTCTAATTGTAGTTGGACGTCCAGGTTTATGGCTGAG

CATAGTGGGGTATCTAA

>M01138:27:000000000-AHTAW:1:1101:11158:4350:dbj|AP012081.1| Margariscus margarita mitochondrial DNA almost complete genome except for D-loop

TAGAACAGGCTCCTCTAGGGAGG-TCTAGGGCACCGTCAGGTCCTTTGGGTTTCACGCTA

ATGCTCGTAGTACCCGGGCGGACGTCTAATTGTAGTTGGACGTCTAGGTTTATGGCTGAG

CATAGTGGGGTATCTAA

>M01138:27:000000000-AHTAW:1:1101:25606:20054:dbj|AP012081.1| Margariscus margarita mitochondrial DNA almost complete genome except for D-loop

TAGAACAGGCTCCTCTAGGGGGG-TCTAAGGCACCGTCAGGTCCTTTGGGTTTCAAGCTA

ATGCTCGTAGTACCCGGGCGGACGTCTAATTGTAGTTGGCCGTCTTGGTTTATGGCTGAG

CATAGTGGGGTATCTAA

>M01138:27:000000000-AHTAW:1:1101:18769:24630:dbj|AP012081.1| Margariscus margarita mitochondrial DNA almost complete genome except for D-loop

TAGAACAGGCTCCTCTAGGGGGG-TCTAAGGCACCGTCAGGTCCTTTGGATTTCAAGCTA

ATGCTCGTAGTACCCGGGCGGACGTCTAATTGTAGTTGGACGTCTAGGTTTATGGCTGGG

CATAGTGGGGTATCTAA

>M01138:27:000000000-AHTAW:1:1101:15106:3955:dbj|AP012081.1| Margariscus margarita mitochondrial DNA almost complete genome except for D-loop

TAGAACAGGCTCCTCTAGGGGGG-TCTAAGGCACCGCCAGGTCCTTTGGGTTTCAAGCTA

ACGCTCGTAGTACCCGGGCGGACGTCTAATTGTAGTTGGACGTCTAGGTTTATGGCTGAG

CATAGTGGGGTATCTAA

>M01138:27:000000000-AHTAW:1:1103:5472:18837:dbj|AP012081.1| Margariscus margarita mitochondrial DNA almost complete genome except for D-loop

TAGAACAGGCTCCTCTAGGGGGG-TCTAAGGCACCGTCAGGTCCTTTGGGTTTCAAGCTA

ATGCTCGTAGTACCCGGGCAGACGCCTAATTGTAGTTGGACGTCTAGGTTTATGGCTGAG

CATAGTGGGGTATCTAA

>M01138:27:000000000-AHTAW:1:1104:25157:3897:dbj|AP012081.1| Margariscus margarita mitochondrial DNA almost complete genome except for D-loop

TAGAACAGGCTCCTCTAGGGGGG-TCTAAGGCACCGTCAGGTCCTTTGGGTTTCAAGCTA

ATGCTCGAAGTACCCGGGCGGGCGTCTAATTGTAGTTGGACGTCTAGGTTTATGGCTGAG

CATAGTGGGGTATCTAA

>M01138:27:000000000-AHTAW:1:1102:8228:21236:dbj|AP012081.1| Margariscus margarita mitochondrial DNA almost complete genome except for D-loop

TAGAACAGGCTCCTCTAGGGGGG-TCTAAGGCACCGTCAGGTCCTTTGGGTTTCAAGCTA

ATGCTCGTAGTACCCGGGCGGACGTCTGATGGTAGTTGGACGTCTAGGTTTATGGCTGAG

CATAGTGGGGTATCTAA

>M01138:27:000000000-AHTAW:1:1101:20295:2151:dbj|AP012081.1| Margariscus margarita mitochondrial DNA almost complete genome except for D-loop

TAGAACAGGCTCCTCTAGGGGGG-TCTAAGGCGACGTCAGGTCCTTTGGGTTTCAAGCTA

ATGCTCGTAGTACCCGGGCGGACGTCTAATTGTAGTTGGACGTCTAGGTTTATGGCTGAG

CATAGTGGGGTATCTAA

>M01138:27:000000000-AHTAW:1:1110:27094:14327:dbj|AP012081.1| Margariscus margarita mitochondrial DNA almost complete genome except for D-loop

TAGAACAGGCTCCTCTAGGGGGG-TCTAAGGCACCGTCAGGTCCTTTGGGTTTCAAGCTA

ATGCTCGCAGTCCCCGGGCGGACGTCTAATTGTAGTTGGACGTCTAGGTTTATGGCTGAG

CATAGTGGGGTATCTAA

>M01138:27:000000000-AHTAW:1:1104:14015:12926:dbj|AP012081.1| Margariscus margarita mitochondrial DNA almost complete genome except for D-loop

TAGAACAGGCTCCTCTAGGGTGG-TCTAAGGCACCGTCAGGTCCTTTGGGTTTCAAGCTA

ATGCTCGTAGTACCCGGGCGGACGTCTAATTGTAGTTGGGCGTCTAGGTTTATGGCTGAG

CATAGTGGGGTATCTAA

>M01138:27:000000000-AHTAW:1:1102:11475:16642:dbj|AP012081.1| Margariscus margarita mitochondrial DNA almost complete genome except for D-loop

TAGAACAGGCTCCTCTAGGGGGG-TCTAAGGCACCGTCAGTTCCTTTGGGTTTCAAGCTA

ATGCTCGTAGTACCCGGGCGGACGTCTAATTGTAGTTGGACGTCTCGGTTTATGGCTGAG

CATAGTGGGGTATCTAA

>M01138:27:000000000-AHTAW:1:1106:22292:19128:dbj|AP012081.1| Margariscus margarita mitochondrial DNA almost complete genome except for D-loop

TAGAACAGGCTCCTCTAGGGGGG-TCTAAGGCACCGTCGGGTCCTTTGGGTTTCGAGCTA

ATGCTCGTAGTACCCGGGCGGACGTCTAATTGTAGTTGGACGTCTAGGTTTATGGCTGAG

CATAGTGGGGTATCTAA

>M01138:27:000000000-AHTAW:1:1104:25267:13555:dbj|AP012081.1| Margariscus margarita mitochondrial DNA almost complete genome except for D-loop

TAGAACAGGCTCCTCTAGGGGGG-TCTAAGGCACCGTCAGGCCCTTTGGGTTTCAAGCTA

GTGCTCGTAGTACCCGGGCGGACGTCTAATTGTAGTTGGACGTCTAGGTTTATGGCTGAG

CATAGTGGGGTATCTAA

>M01138:27:000000000-AHTAW:1:1103:9135:13513:dbj|AP012081.1| Margariscus margarita mitochondrial DNA almost complete genome except for D-loop

TAGAACAGGCTCCTCTAGGGGGG-TCTAAGGCACCGTCAGGTCCTTTGGGTCTCAAGCTA

ATGCTCGTAGTACCCGGGCGGACGTCTCATTGTAGTTGGACGTCTAGGTTTATGGCTGAG

CATAGTGGGGTATCTAA

>M01138:27:000000000-AHTAW:1:1102:9097:5137:dbj|AP012081.1| Margariscus margarita mitochondrial DNA almost complete genome except for D-loop

TAGAACAGGCTCCTCTAGGGGGG-TCTAAGGCACCGTCAGGTCCCTTGGGTTTCAAGCTA

ATGCTCGTAGTACCTGGGCGGACGTCTAATTGTAGTTGGACGTCTAGGTTTATGGCTGAG

CATAGTGGGGTATCTAA

>M01138:27:000000000-AHTAW:1:1101:16888:19229:dbj|AP012081.1| Margariscus margarita mitochondrial DNA almost complete genome except for D-loop

TAGAACAGGCTCCTCTAGGGGGG-TCTAAGGCACCGTCAGGTCCTTTGGGTTTCAAGCTA

ATGCCCGTAGTACCCGGGCGGACGTCTAATTGTAATTGGACGTCTAGGTTTATGGCTGAG

CATAGTGGGGTATCTAA

>M01138:27:000000000-AHTAW:1:1102:8638:17608:dbj|AP012081.1| Margariscus margarita mitochondrial DNA almost complete genome except for D-loop

TAGAACAGGCTCCTCTAGGGGGG-TCTAAGGCACCGTCAGGTCCTTTGGGTTTCAAGCTG

ATGCTCGTAGTACCCGGGCGGACGTCTAGTTGTAGTTGGACGTCTAGGTTTATGGCTGAG

CATAGTGGGGTATCTAA

>M01138:27:000000000-AHTAW:1:1101:15915:21673:dbj|AP012081.1| Margariscus margarita mitochondrial DNA almost complete genome except for D-loop

TAGAACAGGCTCCTCTAGGGGGG-TCTAAGGCACCGTCAGGTCCTTTGGGTTTCAAGCTA

ATGCACGTCGTACCCGGGCGGACGTCTAATTGTAGTTGGACGTCTAGGTTTATGGCTGAG

CATAGTGGGGTATCTAA

>M01138:27:000000000-AHTAW:1:1104:12736:21836:dbj|AP012081.1| Margariscus margarita mitochondrial DNA almost complete genome except for D-loop

TAGAACAGGCTCCTCTAGGGGGG-TCTAAGGCACCGTCAGGTCCTTTGGGTTTCAAGCTA

ATGCTCGTAGTACCCGGGCGGACGTCTAATCGTGGTTGGACGTCTAGGTTTATGGCTGAG

CATAGTGGGGTATCTAA

>M01138:27:000000000-AHTAW:1:1101:11844:14671:dbj|AP012081.1| Margariscus margarita mitochondrial DNA almost complete genome except for D-loop

TAGAACAGGCTCCTCTAGGGGGG-TCTATGGCACCGTCAGGTCCTTTGGGTTTCAAGCTA

ATGCTCGTAGTACCCGGGCGGACGTCTAACTGTAGTTGGACGTCTAGGTTTATGGCTGAG

CATAGTGGGGTATCTAA

>M01138:27:000000000-AHTAW:1:1102:25148:12008:dbj|AP012081.1| Margariscus margarita mitochondrial DNA almost complete genome except for D-loop

TAGAACAGGCTCCTCTAGGGGGG-TCTAGGGCACCGTCAGGTCCTTTGGGTTTCAAGCTA

ATGCTCGTAGTACCCGGGCGGACGTCTAATTGTAGTTGGACGTCTAGATTTATGGCTGAG

CATAGTGGGGTATCTAA

>M01138:27:000000000-AHTAW:1:1102:22394:3509:dbj|AP012081.1| Margariscus margarita mitochondrial DNA almost complete genome except for D-loop

TAGAACAGGCTCCTCTAGGGGGG-TCTAAGGCACCGTCAGGTCCTTCGGGTTTCAAGCTA

ATGCTCGTAGTACCCGGGCGGACGTCCAATTGTAGTTGGACGTCTAGGTTTATGGCTGAG

CATAGTGGGGTATCTAA

>M01138:27:000000000-AHTAW:1:1104:17393:16371:dbj|AP012081.1| Margariscus margarita mitochondrial DNA almost complete genome except for D-loop

TAGAACAGGCTCCTCTAGGGGGG-TCTAAGGCTCCGTCAGGTCCTTTGGGTTCCAAGCTA

ATGCTCGTAGTACCCGGGCGGACGTCTAATTGTAGTTGGACGTCTAGGTTTATGGCTGAG

CATAGTGGGGTATCTAA

>M01138:27:000000000-AHTAW:1:1105:11108:1530:dbj|AP012081.1| Margariscus margarita mitochondrial DNA almost complete genome except for D-loop

TAGAACAGGCTCCTCTAGGGGGG-TCTAAGGCACCGTCAGGTCCTTTGGGTTTCAAGCTA

ATACTCGTAGTACCCGGGCGGACGTCTAATTGTAGTCGGACGTCTAGGTTTATGGCTGAG

CATAGTGGGGTATCTAA

>M01138:27:000000000-AHTAW:1:1104:15703:5912:dbj|AP012081.1| Margariscus margarita mitochondrial DNA almost complete genome except for D-loop

TAGAACAGGCTCCTCTAGGGGGG-TCTAAGGCACCGTCAGGTCCTTTGTGTTTCAAGCTA

ATGCTCGTAGCACCCGGGCGGACGTCTAATTGTAGTTGGACGTCTAGGTTTATGGCTGAG

CATAGTGGGGTATCTAA

>M01138:27:000000000-AHTAW:1:1108:26112:18547:dbj|AP012081.1| Margariscus margarita mitochondrial DNA almost complete genome except for D-loop

TAGAACAGGCTCCTCTAGGGGGG-TTTAAGGCACCGTCAGGTCCTTTGGGTTTCAAGCTA

ATGCTCGTAGTACCCGGGCGGACGTCTAATTGTAGTTGGACCGCTAGGTTTATGGCTGAG

CATAGTGGGGTATCTAA

>M01138:27:000000000-AHTAW:1:1107:20994:21226:dbj|AP012081.1| Margariscus margarita mitochondrial DNA almost complete genome except for D-loop

TAGAACAGGCTCCTCTAGGGGGG-TCTAAGGCACCGTCAGGTCCTTTGGGTTTTAAGCTA

ATGCTCGTAGTACCCGGGCGGACGTCTAATTGTAGTTGGACGTTTAGGTTTATGGCTGAG

CATAGTGGGGTATCTAA

>M01138:27:000000000-AHTAW:1:1102:9808:20605:dbj|AP012081.1| Margariscus margarita mitochondrial DNA almost complete genome except for D-loop

TAGAACAGGCTCCTCTAGGGGGG-TCTAAGGCACCGTCAGGTCCTTTGGGTTTCAAGCTA

ATGCTCGTAGTACCCGGGCGGACGGCTAATTGTCGTTGGACGTCTAGGTTTATGGCTGAG

CATAGTGGGGTATCTAA

>M01138:27:000000000-AHTAW:1:1109:20542:9635:dbj|AP012081.1| Margariscus margarita mitochondrial DNA almost complete genome except for D-loop

TAGAACAGGCTCCTCTAGGGGGG-TCTAAGGCACCGTCAGGTCCTCTGGGATTCAAGCTA

ATGCTCGTAGTACCCGGGCGGACGTCTAATTGTAGTTGGACGTCTAGGTTTATGGCTGAG

CATAGTGGGGTATCTAA

>M01138:27:000000000-AHTAW:1:1101:6380:15487:dbj|AP012081.1| Margariscus margarita mitochondrial DNA almost complete genome except for D-loop

TAGAACAGGCTCCTCTAGAGAGG-TCTAAGGCACCGTCAGGTCCTTTGGGTTTCAAGCCA

ATGCTCGTAGTACCCGGGCGGACGTCTAATTGTAGTTGGACGTCTAGGTTTATGGCTGAG

CATAGTGGGGTATCTAA

>M01138:27:000000000-AHTAW:1:1102:19606:3716:dbj|AP012081.1| Margariscus margarita mitochondrial DNA almost complete genome except for D-loop

TAGAACAGGCTCCTCTAGGGGGG-TCTAAGGCACTGTCAGGTCCTTTGGGTTTCAAGCTA

ATGTTCGTAGTACCCGGGCGGACGTCTAATTGTAGTTGGACGTCTAGGTTTATGGCTGAG

CATAGTGGGGTATCTAA

>M01138:27:000000000-AHTAW:1:1101:11096:18584:dbj|AP012081.1| Margariscus margarita mitochondrial DNA almost complete genome except for D-loop

TAGAACAGGCTCCTCTAGGGGGG-TCTAAGGCACCGTCAGGTCCTTTGGGTTTCAAGCTA

ATGCTCGTAGTACTCGGGCGGACGTCTAATTGTAGTTGGACGTCTAGGTTTCTGGCTGAG

CATAGTGGGGTATCTAA

>M01138:27:000000000-AHTAW:1:1111:24699:18639:dbj|AP012081.1| Margariscus margarita mitochondrial DNA almost complete genome except for D-loop

TAGAACAGGCTCCTCTAGGGGGG-TCTAAGGCACCGTCAGGTCCTTTGGGTTTCAGGCTA

ATGATCGTAGTACCCGGGCGGACGTCTAATTGTAGTTGGACGTCTAGGTTTATGGCTGAG

CATAGTGGGGTATCTAA

>M01138:27:000000000-AHTAW:1:1119:5802:19611:dbj|AP012081.1| Margariscus margarita mitochondrial DNA almost complete genome except for D-loop

TAGAACAGGCTCCTCTAGGGGGG-TCTAAGGCACCGTCAGGTCCTTTGGGTTTCAAGCTA

ATGCTCGTAGTACCCGGGTGGACGTCTAATTGTAGTTGGACGTCTAGGTTTATGACTGAG

CATAGTGGGGTATCTAA

>M01138:27:000000000-AHTAW:1:1101:15094:23674:dbj|AP012081.1| Margariscus margarita mitochondrial DNA almost complete genome except for D-loop

TAGAACAGGCTCCTCTAGGGAGG-TCTAAGGCACTGTCAGGTCCTTTGGGTTTCAAGCTA

ATGCTCGTCGTACCCGGGCGGACGTCTAATTGTAGTTGGACGTCTAGGTTTATGGCTGAG

CATAGTGGGGTATCTAA

>M01138:27:000000000-AHTAW:1:1103:25569:9876:dbj|AP012081.1| Margariscus margarita mitochondrial DNA almost complete genome except for D-loop

TAGAACAGGCTCCTCTAGGGAGG-TCTAAGGCACCGTCAGGTCCTTTGGGTTTCAAGCTA

ATGCTCGTAGTACCTGGGCGGACGCCTAATTGTAGTTGGACGTCTAGGTTTATGGCTGAG

CATAGTGGGGTATCTAA

>M01138:27:000000000-AHTAW:1:1103:8822:7569:dbj|AP012081.1| Margariscus margarita mitochondrial DNA almost complete genome except for D-loop

TAGAACAGGCTCCTCTAGGGGGG-TCTAAAGCACCATCAGGTCCTTTGGGTTTCAAGCTA

ATGCTCGTAGTACCCGGGCGGACGTCTAATTGTAGTTGGACGTCTAGGTTTATGGCTGAG

CATAGTGGGGTATCTAA

>M01138:27:000000000-AHTAW:1:1116:19855:19317:dbj|AP012081.1| Margariscus margarita mitochondrial DNA almost complete genome except for D-loop

TAGAACAGGCTCCTCTAGGGGGG-TCCAAGGCACCGTCAGGTCCTTTGGGTTTCAAGCTA

ATGCTCGTAGTACCCGGGCGGACGTCTAATTGTAGTTGGACGTCTAGGTTCATGGCTGAG

CATAGTGGGGTATCTAA

>M01138:27:000000000-AHTAW:1:1103:20447:21321:dbj|AP012081.1| Margariscus margarita mitochondrial DNA almost complete genome except for D-loop

TAGAACAGGCTCCTCTAGGGGGG-TTTAAGGCACCGTCAGTTCCTTTGGGTTTCACGCTA

ATGCTCGTAGTACCCGGGCGGACGTCTAATTGTAGTTGGACGTCTAGGTTTATGGCTGAG

CATAGTGGGGTATCTAA

>M01138:27:000000000-AHTAW:1:1101:26518:8797:dbj|AP012081.1| Margariscus margarita mitochondrial DNA almost complete genome except for D-loop

TAGAACAGGCTCCTCTAGGGGGG-TCTAAGGCACCGTCAGGTCCTTTGGGTTTCAAGCTA

ATGCTCGTAGTACCCGGGCGGACGTCTATTTGTAGTTGGTCGTCTAGGTTTATGGCTGAG

CATAGTGGGGTATCTAA

>M01138:27:000000000-AHTAW:1:1113:10323:24130:dbj|AP012081.1| Margariscus margarita mitochondrial DNA almost complete genome except for D-loop

TAGAACAGGCTCCTCTAGGGAGG-TCTAAGGCACCGTCAGGTCCTTTGGGCTTCAAGCTA

ATGCTCGTAGTACCCGGGCGGACGTCTAATTGTAGTTGGACGTCTAGGTCTATGGCTGAG

CATAGTGGGGTATCTAA

>M01138:27:000000000-AHTAW:1:1104:12214:1285:dbj|AP012081.1| Margariscus margarita mitochondrial DNA almost complete genome except for D-loop

TAGAACAGGCTCCTCTAGGGGGG-TCTAAGGCACCGTCAGATCCTTTGGGTTTCAAGCTA

ATGCTCGTAGTACCCGGGCGGACGTCTAATTGTAGTTGGACGTCTAGGTTTTTGGCTGAG

CATAGTGGGGTATCTAA

>M01138:27:000000000-AHTAW:1:1102:10421:23833:dbj|AP012081.1| Margariscus margarita mitochondrial DNA almost complete genome except for D-loop

TAGAACAGGCTCCTCTAGGGGGG-TCTAAGGCACCGTCAGGTCCTTTGGGTTTCAAGCTA

ATGCTCGTAGTACCCGGGCGGACGTCTAATTGTAGTTGGACGTCTAGGTTTAGGGCTAAG

CATAGTGGGGTATCTAA

>M01138:27:000000000-AHTAW:1:1105:10202:7542:dbj|AP012081.1| Margariscus margarita mitochondrial DNA almost complete genome except for D-loop

TAGAACAGGCTCCTCTAGTGGGG-TCTAAGGTACCGTCAGGTCCTTTGGGTTTCAAGCTA

ATGCTCGTAGTACCCGGGCGGACGTCTAATTGTAGTTGGACGTCTAGGTTTATGGCTGAG

CATAGTGGGGTATCTAA

>M01138:27:000000000-AHTAW:1:1116:27502:15284:dbj|AP012081.1| Margariscus margarita mitochondrial DNA almost complete genome except for D-loop

TAGAACAGGCTCCTCTAGGGGGG-TCTAAGGCACCGTCAGGTCCTTTGGGTTTCAAGCTA

ATGCTCGTAGTACCCGGGCGGACGACTAAGTGTAGTTGGACGTCTAGGTTTATGGCTGAG

CATAGTGGGGTATCTAA

>M01138:27:000000000-AHTAW:1:1101:16613:8351:dbj|AP012081.1| Margariscus margarita mitochondrial DNA almost complete genome except for D-loop

TAGAACAGGCTCCTCTAGGGGGG-TATAAGGCACCGTCAGGTCCTTTGGGTTTCAAGCTA

ATGCTCGTAGTACCCGGGCGGACGTCTAATTGTAGTTAGACGTCTAGGTTTATGGCTGAG

CATAGTGGGGTATCTAA

>M01138:27:000000000-AHTAW:1:1105:18713:12654:dbj|AP012081.1| Margariscus margarita mitochondrial DNA almost complete genome except for D-loop

TAGAACAGGCTCCTCTAGGGGGG-TCTAAGGCACCGTCAGGTCCTTTGGGTTTCAAGCAA

ATGCTCGTAGTACCCGAGCGGACGTCTAATTGTAGTTGGACGTCTAGGTTTATGGCTGAG

CATAGTGGGGTATCTAA

>M01138:27:000000000-AHTAW:1:1114:11045:10388:dbj|AP012081.1| Margariscus margarita mitochondrial DNA almost complete genome except for D-loop

TAGAACAGGCTCCTCTAGGGGGG-TCTAAGGCACCGTCAAGTCCTTTGGGTTTCAAGCTA

ATGCTCGTAGTACCCGGGCGGACGTCTAATTGTAGTTGGACGTATAGGTTTATGGCTGAG

CATAGTGGGGTATCTAA

>M01138:27:000000000-AHTAW:1:1101:28561:7828:dbj|AP012081.1| Margariscus margarita mitochondrial DNA almost complete genome except for D-loop

TAGAACAGGCTCCTCTAGGGAGG-TCTAAGGCACCGTCAGGTCCTTTGGGTTTCAAGCTA

ATGCTCGTAGTACCCTGGCGGACGTCTGATTGTAGTTGGACGTCTAGGTTTATGGCTGAG

CATAGTGGGGTATCTAA

>M01138:27:000000000-AHTAW:1:1102:27809:6667:dbj|AP012081.1| Margariscus margarita mitochondrial DNA almost complete genome except for D-loop

TAGAACAGGCTCCTCTAGGGGGG-TCTAAGGCACCGTCAGGTCCTTTGGGTTTCAAGCTA

ATGCTCGTAGTACCCTTGCGGACGTCTAATTGTAGTTGGACGTCTAGGTTTATGGCTGAG

CATAGTGGGGTATCTAA

>M01138:27:000000000-AHTAW:1:1105:28578:17978:dbj|AP012081.1| Margariscus margarita mitochondrial DNA almost complete genome except for D-loop

TAGAACAGGCTCCTCTAGGGGGG-TCTAAGGCACCGTCAGGTCCTTTGGGTTTGAAGCTA

ATGCTCGTAGTACCCGGGCGGACGTCTAATTGTAGTTGGACGTCTAGGATTATGGCTGAG

CATAGTGGGGTATCTAA

>M01138:27:000000000-AHTAW:1:1106:26657:19417:dbj|AP012081.1| Margariscus margarita mitochondrial DNA almost complete genome except for D-loop

TAGAACAGGCTCCTCTAGGGAGG-TCTAAGGCACCGTCAGGTCCTTTGGGTTTCAAGCTA

ATGCTCGTAGTACCCGGGCGGACGTCTAACTGTAGTTGGACGTCTCGGTTTATGGCTGAG

CATAGTGGGGTATCTAA

>M01138:27:000000000-AHTAW:1:1103:14332:6126:dbj|AP012081.1| Margariscus margarita mitochondrial DNA almost complete genome except for D-loop

TAGAACAGGCTCCTCTAGGGAGG-TCTAAGGCACCGTCAGGTCCTTTGGGTTCTAAGCTA

ATGCTCGTAGTACCCGGGCGGACGTCTAATTGTAGTTGGACGTCTAGGTTTATGGCTGAG

CATAGTGGGGTATCTAA

>M01138:27:000000000-AHTAW:1:1110:16798:10533:dbj|AP012081.1| Margariscus margarita mitochondrial DNA almost complete genome except for D-loop

TAGAACAGGCTCCTCTAGGGAGG-TCTAAGGCACCGTCAGGTCCTTTGGGTTTCAAGCTA

ATGCTCGTAGTACCCGGGCGGGCGTCTAATTGTAGTTGGACGACTAGGTTTATGGCTGAG

CATAGTGGGGTATCTAA

>M01138:27:000000000-AHTAW:1:1107:13687:18262:dbj|AP012081.1| Margariscus margarita mitochondrial DNA almost complete genome except for D-loop

TAGAACAGGCTCCTCTAGGGGGG-TCTAAGGCACCGTCAGGTCGTGTGGGTTTCAAGCTA

ATGCTCGTAGTACCCGGGCGGACGTCTAATTGTAGTTGGACGTCTAGGTTTATGGCTGAG

CATAGTGGGGTATCTAA

>M01138:27:000000000-AHTAW:1:1108:17029:5464:dbj|AP012081.1| Margariscus margarita mitochondrial DNA almost complete genome except for D-loop

TAGAACAGGCTCCTCTAGGGAGG-TCTAAGGCACCGTCAGGTCCCTTGGGTTTCAAGCTA

ATGCTCGTAGTACCCGGGCGGACGTCTAATTGTGGTTGGACGTCTAGGTTTATGGCTGAG

CATAGTGGGGTATCTAA

>M01138:27:000000000-AHTAW:1:1111:10020:18986:dbj|AP012081.1| Margariscus margarita mitochondrial DNA almost complete genome except for D-loop

TAGAACAGGCTCCTCTAGGGGGG-TCTAAGGCACCGTCAGGTCCTATGGGTTTCAAGCTA

ATGCTCGTAGTACCCGGGCGGTCGTCTAATTGTAGTTGGACGTCTAGGTTTATGGCTGAG

CATAGTGGGGTATCTAA

>M01138:27:000000000-AHTAW:1:1117:6623:17309:dbj|AP012081.1| Margariscus margarita mitochondrial DNA almost complete genome except for D-loop

TAGAACAGGCTCCTCTAGGGAGG-TCTAAGGCACCGTCAGGTCCTTTGGGTCTCAAGCTA

ATGCTCGTAGTACCCGGGCGGACGTCTAATTGCAGTTGGACGTCTAGGTTTATGGCTGAG

CATAGTGGGGTATCTAA

>M01138:27:000000000-AHTAW:1:1103:23161:5244:dbj|AP012081.1| Margariscus margarita mitochondrial DNA almost complete genome except for D-loop

TAGAACAGGCTCCTCTAGGGGGG-TCTAAGGCACCGTCAGGTCCTTTTGGTTTCAAGCTA

ATGCTCGTAGTACCCGGGCGGACGTCTAATTTTAGTTGGACGTCTAGGTTTATGGCTGAG

CATAGTGGGGTATCTAA

>M01138:27:000000000-AHTAW:1:1107:26097:16531:dbj|AP012081.1| Margariscus margarita mitochondrial DNA almost complete genome except for D-loop

TAGAACAGGCTCCTCTAGAGGGG-TCTAAGGCACCGTCAGGTCCTTTGGGTTTCAAGCTA

ATGCTCGTTGTACCCGGGCGGACGTCTAATTGTAGTTGGACGTCTAGGTTTATGGCTGAG

CATAGTGGGGTATCTAA

>M01138:27:000000000-AHTAW:1:1101:22066:2732:dbj|AP012081.1| Margariscus margarita mitochondrial DNA almost complete genome except for D-loop

TAGAACAGGCTCCTCTAGGGAGG-TCTATGGCACCGTCAGGTCCTCTGGGTTTCAAGCTA

ATGCTCGTAGTACCCGGGCGGACGTCTAATTGTAGTTGGACGTCTAGGTTTATGGCTGAG

CATAGTGGGGTATCTAA

>M01138:27:000000000-AHTAW:1:1108:3572:12101:dbj|AP012081.1| Margariscus margarita mitochondrial DNA almost complete genome except for D-loop

TAGAACAGGCTCCTCTAGGGGGG-TCTAAGGCACGGTCAGGTCCTTTGGGTTTCAAGCTA

ATGCTCGTAGTACCCGGGAGGACGTCTAATTGTAGTTGGACGTCTAGGTTTATGGCTGAG

CATAGTGGGGTATCTAA

>M01138:27:000000000-AHTAW:1:1101:18733:17680:dbj|AP012081.1| Margariscus margarita mitochondrial DNA almost complete genome except for D-loop

TAGAACAGGCTCCTCTAGGGGGG-TCTAAGGCACCGTCAGGTCCTTTGGGTTTCAAGCTA

ATGCTCGTAGTACCCGGGCGGACGTCTAATTGTAGTTGGACGTCTAGGTTTATGGCATAG

CATAGTGGGGTATCTAA

>M01138:27:000000000-AHTAW:1:1107:11102:7178:dbj|AP012081.1| Margariscus margarita mitochondrial DNA almost complete genome except for D-loop

TAGAACAGGCTCCTCTAGGGGGG-TCTAAGGCACCGTCAGCTCGTTTGGGTTTCAAGCTA

ATGCTCGTAGTACCCGGGCGGACGTCTAATTGTAGTTGGACGTCTAGGTTTATAGCTGAG

CATAGTGGGGTATCTAA

>M01138:27:000000000-AHTAW:1:1107:23768:5029:dbj|AP012081.1| Margariscus margarita mitochondrial DNA almost complete genome except for D-loop

TAGAACAGGCTCCTCTAGGGAGG-TCTAAGGCACCGTCAGGTCCTTTGGGTTTCAAGCTA

ATGCTCGCAGCACCCGGGCGGACGTCTAATTGTAGTTGGACGTCTAGGTTTATGGCTGAG

CATAGTGGGGTATCTAA

>M01138:27:000000000-AHTAW:1:1115:18659:4267:dbj|AP012081.1| Margariscus margarita mitochondrial DNA almost complete genome except for D-loop

TAGAACAGGCTCCTCTAGGGAGG-TCTAAGGCACCGTCAGGTCCTTTGGGTTTCAAGCTA

ATGCCCGTAGGACCCGGGCGGACGTCTAATTGTAGTTGGACGTCTAGGTTTATGGCTGAG

CATAGTGGGGTATCTAA

>M01138:27:000000000-AHTAW:1:1119:22867:15897:dbj|AP012081.1| Margariscus margarita mitochondrial DNA almost complete genome except for D-loop

TAGAACAGGCTCCTCTAGGGGGG-TCTAAGGCACCGTCAGGTCCTTTGGGTTTCAAGCTA

ATGCTCGTAGTATCCGGACGGACGTCTAATTGTAGTTGGACGTCTAGGTTTATGGCTGAG

CATAGTGGGGTATCTAA

>M01138:27:000000000-AHTAW:1:1107:16359:11362:dbj|AP012081.1| Margariscus margarita mitochondrial DNA almost complete genome except for D-loop

TAGAACAGGCTCCTCTAGGGGGG-TCTAAGGCACCGTCAGGTCCATTGGGTTTAAAGCTA

ATGCTCGTAGTACCCGGGCGGACGTCTAATTGTAGTTGGACGTCTAGGTTTATGGCTGAG

CATAGTGGGGTATCTAA

>M01138:27:000000000-AHTAW:1:1107:18384:15762:dbj|AP012081.1| Margariscus margarita mitochondrial DNA almost complete genome except for D-loop

TAGAACAGGCTCCTCTAGGGGGG-TCTAAGGCACCGTCAGGTCCTTTGGGTTTCACGTTA

ATGCTCGTCGTACCCGGGCGGACGTCTAATTGTAGTTGGACGTCTAGGTTTATGGCTGAG

CATAGTGGGGTATCTAA

>M01138:27:000000000-AHTAW:1:1111:5440:10443:dbj|AP012081.1| Margariscus margarita mitochondrial DNA almost complete genome except for D-loop

TAGAACAGGCTCCTCTAGGGGGG-TTTAAGGCACCGTCAGGTCCTTTGGGTTTCAAGTTA

ATGCTCGTAGTACCCGGGCGGACGTCTAATTGTAGTTGGGCGTCTAGGTTTATGGCTGAG

CATAGTGGGGTATCTAA

>M01138:27:000000000-AHTAW:1:1117:5340:4025:dbj|AP012081.1| Margariscus margarita mitochondrial DNA almost complete genome except for D-loop

TAGAACAGGCTCCTCTAGGGGGG-TCTAAGGCACCGTCAGGTCCTTTGGGTTTCAAGCTA

ATGCTCGTAGTACCCGGGCGGACGTCTAATTGTAGTTGGACGGCTAGGTATATGGCTGAG

CATAGTGGGGTATCTAA

>M01138:27:000000000-AHTAW:1:1106:3117:13982:dbj|AP012081.1| Margariscus margarita mitochondrial DNA almost complete genome except for D-loop

TAGAACAGGCTCCTCTAGGGGGG-TCTAAGGCACCGTCAGGTCCTTTGGGTTTCAAGCTC

ATGCTCGTAGTACCCGGGCGGACGTCTAATTGTAGTTGGACGTCTAGGTTTATTGCTGAG

CATAGTGGGGTATCTAA

>M01138:27:000000000-AHTAW:1:1109:23081:20953:dbj|AP012081.1| Margariscus margarita mitochondrial DNA almost complete genome except for D-loop

TAGAACAGGCTCCTCTAGGGGGG-TCTAAGGCACAGTCAGGTCCTTTGGGTTTCAAGCTA

ATGCTCGTAGTACCCGGGCGGACGTCTACTTGTAGTTGGACGTCTAGGTTTATGGCTGAG

CATAGTGGGGTATCTAA

>M01138:27:000000000-AHTAW:1:1104:8659:14806:dbj|AP012081.1| Margariscus margarita mitochondrial DNA almost complete genome except for D-loop

TAGAACAGGCTCCTCTAGGGAGG-TCTAAGGCGCCGTCAGGTCCTTTGGGTTTCAAGCTA

ATGCTCGTAGTACTCGGGCGGACGTCTAATTGTAGTTGGACGTCTAGGTTTATGGCTGAG

CATAGTGGGGTATCTAA

>M01138:27:000000000-AHTAW:1:1112:16288:18435:dbj|AP012081.1| Margariscus margarita mitochondrial DNA almost complete genome except for D-loop

TAGAACAGGCTCCTCTAGGGGGG-TCTAAGGCACCGTCAGGTTCTTTGGGTTTCAAGCTA

ATGCTCGTAGTACCCGGGCGGACGTCTAATTGTAGTGGGACGTCTAGGTTTATGGCTGAG

CATAGTGGGGTATCTAA

>M01138:27:000000000-AHTAW:1:1119:14175:16499:dbj|AP012081.1| Margariscus margarita mitochondrial DNA almost complete genome except for D-loop

TAGAACAGGCTCCTCTAGGGAGG-TCTAAGGCACCGTCAGGTCCTTTGGGTTTCGAGCTA

GTGCTCGTAGTACCCGGGCGGACGTCTAATTGTAGTTGGACGTCTAGGTTTATGGCTGAG

CATAGTGGGGTATCTAA

>M01138:27:000000000-AHTAW:1:1103:4252:20112:dbj|AP012081.1| Margariscus margarita mitochondrial DNA almost complete genome except for D-loop

TAGAACAGGCTCCTCTAGGGAGG-TCTAAGGCACCGTCAGGTCCTTTTGGTTTCAAGCTA

ATGCTCGTAGTACCCGGGCGGACGTCTAATGGTAGTTGGACGTCTAGGTTTATGGCTGAG

CATAGTGGGGTATCTAA

>M01138:27:000000000-AHTAW:1:1110:6635:11866:dbj|AP012081.1| Margariscus margarita mitochondrial DNA almost complete genome except for D-loop

TAGAACAGGCTCCTCTAGGGAGG-TCTAAGGCACCGTCGGGTCCTTTGGGTTTCAAGCTA

CTGCTCGTAGTACCCGGGCGGACGTCTAATTGTAGTTGGACGTCTAGGTTTATGGCTGAG

CATAGTGGGGTATCTAA

>M01138:27:000000000-AHTAW:1:1112:20110:21854:dbj|AP012081.1| Margariscus margarita mitochondrial DNA almost complete genome except for D-loop

TAGAACAGGCTCCTCTAGGGAGG-CCTAAGGCACCGTCAGGTCCTTTGGGTTTCAAGCTA

ATGCTCGTAGTACCCGAGCGGACGTCTAATTGTAGTTGGACGTCTAGGTTTATGGCTGAG

CATAGTGGGGTATCTAA

>M01138:27:000000000-AHTAW:1:1103:27794:7838:dbj|AP012081.1| Margariscus margarita mitochondrial DNA almost complete genome except for D-loop

TAGAACAGGCTCCTCTAGGGAGG-TCTAAGGCACCGACAGGTCCTTTGGGTTTCAAGCTA

ATGCTCGTAGTCCCCGGGCGGACGTCTAATTGTAGTTGGACGTCTAGGTTTATGGCTGAG

CATAGTGGGGTATCTAA

>M01138:27:000000000-AHTAW:1:1106:21805:10158:dbj|AP012081.1| Margariscus margarita mitochondrial DNA almost complete genome except for D-loop

TAGAACAGGCTCCTCTAGGGAGG-TCTAAGGCATCGTCAGGCCCTTTGGGTTTCAAGCTA

ATGCTCGTAGTACCCGGGCGGACGTCTAATTGTAGTTGGACGTCTAGGTTTATGGCTGAG

CATAGTGGGGTATCTAA

>M01138:27:000000000-AHTAW:1:1111:3872:8810:dbj|AP012081.1| Margariscus margarita mitochondrial DNA almost complete genome except for D-loop

TAGAACAGGCTCCTCTAGGGGGG-TCTAAGGCACCGTCAGGTCCTTTGGGTTTCAAGCTA

CTGCTCGTAGTACCCGGTCGGACGTCTAATTGTAGTTGGACGTCTAGGTTTATGGCTGAG

CATAGTGGGGTATCTAA

>M01138:27:000000000-AHTAW:1:1104:9426:7761:dbj|AP012081.1| Margariscus margarita mitochondrial DNA almost complete genome except for D-loop

TAGAACAGGCTCCTCTAGGGAGG-TCTAAGGCACCGTCAGGTCCTTTGGGTTTCACGCTA

ATGCTCGTCGTACCCGGGCGGACGCCTAATTGTAGTTGGACGTCTAGGTTTATGGCTGAG

CATAGTGGGGTATCTAA

>M01138:27:000000000-AHTAW:1:1105:21245:13402:dbj|AP012081.1| Margariscus margarita mitochondrial DNA almost complete genome except for D-loop

TAGAACAGGCTCCTCTAGAGGGG-GCTAAGGCACCGTCAGGTCCTTTGGGTTTCAAGCTA

ATGCTCGTAGTACCCGGGCGGAAGTCTAATTGTAGTTGGACGTCTAGGTTTATGGCTGAG

CATAGTGGGGTATCTAA

>M01138:27:000000000-AHTAW:1:1110:25693:14065:dbj|AP012081.1| Margariscus margarita mitochondrial DNA almost complete genome except for D-loop

TAGAACAGGCTCCTCTAGGGAGG-TCTAAGGCACCGGCAGGTCCTTTGGGTTTCAAGTTA

ATGCTCGTAGTACCCGGGCGGACGTCTAATTGTAGTTGGACGTCTAGGTTTATGGCTGAG

CATAGTGGGGTATCTAA

>M01138:27:000000000-AHTAW:1:1111:13724:19413:dbj|AP012081.1| Margariscus margarita mitochondrial DNA almost complete genome except for D-loop

TAGAACAGGCTCCTCTAGGGGGG-TCTAAGGCACCGTCAGGTCCTTTGGGTATCAAGCTA

ATGCTCGTAGTACACGGGCGGACGTCTAATTGTAGTTGGACGTCTAGGTTTATGGCTGAG

CATAGTGGGGTATCTAA

>M01138:27:000000000-AHTAW:1:1111:8139:19925:dbj|AP012081.1| Margariscus margarita mitochondrial DNA almost complete genome except for D-loop

TAGAACAGGCTCCTCTAGGGGGG-TCTAAGGCACCGTCAGGTCCTTTGGGTTTCAAACTA

ATGCTCGTAGTACCCGGGCGGACGTCTAATTGTAGGTGGACGTCTAGGTTTATGGCTGAG

CATAGTGGGGTATCTAA

>M01138:27:000000000-AHTAW:1:1116:12761:18058:dbj|AP012081.1| Margariscus margarita mitochondrial DNA almost complete genome except for D-loop

TAGAACAGGCTCCTCTAGTGAGG-TCCAAGGCACCGTCAGGTCCTTTGGGTTTCAAGCTA

ATGCTCGTAGTACCCGGGCGGACGTCTAATTGTAGTTGGACGTCTAGGTTTATGGCTGAG

CATAGTGGGGTATCTAA

>M01138:27:000000000-AHTAW:1:1103:16387:11033:dbj|AP012081.1| Margariscus margarita mitochondrial DNA almost complete genome except for D-loop

TAGAACAGGCTCCTCTAGGGAGG-TCTAAGGCACCGTCAGGTCCTTTGGGTTTCATGCTA

ATGCTCGTAGTACCCGGGCGGACGTCTAGTTGTAGTTGGACGTCTAGGTTTATGGCTGAG

CATAGTGGGGTATCTAA

>M01138:27:000000000-AHTAW:1:2103:11803:13558:dbj|AP012081.1| Margariscus margarita mitochondrial DNA almost complete genome except for D-loop

TAGAACAGGCTCCTCTAGGGGGG-TCTAAGGCACCGTCAGGTCCTTTGGGTTTCAAGCTA

ATGCTCGTAGTACCCGGGCGGACGTCTAATTGTTGTTGGACGTCTAGGTTTATGGTTGAG

CATAGTGGGGTATCTAA

>M01138:27:000000000-AHTAW:1:1107:13851:20424:dbj|AP012081.1| Margariscus margarita mitochondrial DNA almost complete genome except for D-loop

TAGAACAGGCTCCTCTAGGGGGG-TCTAAGGTGCCGTCAGGTCCTTTGGGTTTCAGGCTA

ATGCTCGTAGTACCCGGGCGGACGTCTAATTGTAGTTGGACGTCTAGGTTTATGGCTGAG

CATAGTGGGGTATCTAA

>M01138:27:000000000-AHTAW:1:1111:3547:6694:dbj|AP012081.1| Margariscus margarita mitochondrial DNA almost complete genome except for D-loop

TAGAACAGGCTCCTCTAGGGAGG-TCTAAGGCACCGTCAGGTCCGTTGGGTTTCAAGCTA

ATGCTCATAGTACCCGGGCGGACGTCTAATTGTAGTTGGACGTCTAGGTTTATGGCTGAG

CATAGTGGGGTATCTAA

>M01138:27:000000000-AHTAW:1:1111:8065:19802:dbj|AP012081.1| Margariscus margarita mitochondrial DNA almost complete genome except for D-loop

TAGAACAGGCTCCTCTAGGGAGG-TCTAAGGCACCGTCAGGACCTTTGGGTTTCAAGCTA

ATGCTCGTAGTACCCGGGCGGACGTCTAATTGTCGTTGGACGTCTAGGTTTATGGCTGAG

CATAGTGGGGTATCTAA

>M01138:27:000000000-AHTAW:1:1104:27831:14589:dbj|AP012081.1| Margariscus margarita mitochondrial DNA almost complete genome except for D-loop

TAGAACAGGCTCCTCTAGGGGGG-TCTAAGGCCCCGTCAGGTCCTTTGGGTTTCAAGCTA

ATGCTAGTAGTACCCGGGCGGACGTCTAATTGTAGTTGGACGTCTAGGTTTATGGCTGAG

CATAGTGGGGTATCTAA

>M01138:27:000000000-AHTAW:1:1107:18754:5314:dbj|AP012081.1| Margariscus margarita mitochondrial DNA almost complete genome except for D-loop

TAGAACAGGCTCCTCTAGGGGGG-TCTAAGGCACCGTCAGGTCCTTTGGGGTTCAAGCTA

ATGCTCGTAGTACCCGGGCGGACGTCTAAGTGTCGTTGGACGTCTAGGTTTATGGCTGAG

CATAGTGGGGTATCTAA

>M01138:27:000000000-AHTAW:1:1110:3411:7750:dbj|AP012081.1| Margariscus margarita mitochondrial DNA almost complete genome except for D-loop

TAGAACAGGCTCCTCTAGGGGGG-TCTAAGGCACCGGCAGGTCCTTTGGGTTTCAAGCTA

ATGCTCGTAGTACCCGGGCGGACGTCTAATTGTAGTAGGACGTCTAGGTTTATGGCTGAG

CATAGTGGGGTATCTAA

>M01138:27:000000000-AHTAW:1:1113:24686:9018:dbj|AP012081.1| Margariscus margarita mitochondrial DNA almost complete genome except for D-loop

TAGAACAGGCTCCTCTAGGGAGA-TCTAAGGCACCGTCAGGTCCTTTGGGTTTCAAGCTA

ATGCTCGTGGTACCCGGGCGGACGTCTAATTGTAGTTGGACGTCTAGGTTTATGGCTGAG

CATAGTGGGGTATCTAA

>M01138:27:000000000-AHTAW:1:1118:15938:13355:dbj|AP012081.1| Margariscus margarita mitochondrial DNA almost complete genome except for D-loop

TAGAACAGGCTCCTCTAGGGGGG-TCTAAGGCACCGTCAGGTCCTTTGGGTTTCAAGCTA

ATGCTCGTAGTACCCGGGCGGACGTGTAATTGTAGTTGGACGACTAGGTTTATGGCTGAG

CATAGTGGGGTATCTAA

>M01138:27:000000000-AHTAW:1:2105:6138:16145:dbj|AP012081.1| Margariscus margarita mitochondrial DNA almost complete genome except for D-loop

TAGAACAGGCTCCTCTAGGGGTG-TCTAAGGCACCGTCAGGTCCTTGGGGTTTCAAGCTA

ATGCTCGTAGTACCCGGGCGGACGTCTAATTGTAGTTGGACGTCTAGGTTTATGGCTGAG

CATAGTGGGGTATCTAA

>M01138:27:000000000-AHTAW:1:1109:9074:9113:dbj|AP012081.1| Margariscus margarita mitochondrial DNA almost complete genome except for D-loop

TAGAACAGGCTCCTCTAGGGAGG-TCTAAGGCACCGTCAGGTCCTTTGGGTTTCAAGCTA

ATGCTCGTAGTAACCGGGCGGACGTCTAATTGTAGATGGACGTCTAGGTTTATGGCTGAG

CATAGTGGGGTATCTAA

>M01138:27:000000000-AHTAW:1:1116:6926:5534:dbj|AP012081.1| Margariscus margarita mitochondrial DNA almost complete genome except for D-loop

TAGAACAGGCTCCTCTAGGGAGG-TCTAAGGCACCGTCAGGTTCTGTGGGTTTCAAGCTA

ATGCTCGTAGTACCCGGGCGGACGTCTAATTGTAGTTGGACGTCTAGGTTTATGGCTGAG

CATAGTGGGGTATCTAA

>M01138:27:000000000-AHTAW:1:1108:7253:22612:dbj|AP012081.1| Margariscus margarita mitochondrial DNA almost complete genome except for D-loop

TAGAACAGGCTCCTCTAGGGGGG-TCTAAGGCACCGTCAGGTCCTTTGGGTTTCAAGCTA

ATGCTCGTAGTACCCGGGCGGACGTCCATGTGTAGTTGGACGTCTAGGTTTATGGCTGAG

CATAGTGGGGTATCTAA

>M01138:27:000000000-AHTAW:1:1109:24946:11933:dbj|AP012081.1| Margariscus margarita mitochondrial DNA almost complete genome except for D-loop

TAGAACAGGCTCCTCTAGGGAGG-TCTAAGGCACCGTCAGGTCCTTTGGGTTTCAAGCTA

ATGCTCGTAGTACCCGGGCGGACGGATAATTGTAGTTGGACGTCTAGGTTCATGGCTGAG

CATAGTGGGGTATCTAA

>M01138:27:000000000-AHTAW:1:1110:26748:14531:dbj|AP012081.1| Margariscus margarita mitochondrial DNA almost complete genome except for D-loop

TAGAACAGGCTCCTCTAGGGAGG-TCTAAGGCACCGTCAGGTCCTTTGTGTTTCAAGCTA

ATGCTCGTAGTACCCGGGCGGACGTCTAATTGTAGTTGGACGTCTAGGTTTATGGTTGAG

CATAGTGGGGTATCTAA

>M01138:27:000000000-AHTAW:1:2101:10643:14721:dbj|AP012081.1| Margariscus margarita mitochondrial DNA almost complete genome except for D-loop

TAGAACAGGCTCCTCTAGGGGGG-TCTAAGGCACCGTCAGGTCCTTTGGGTTTCAAGCTA

ATGCTCGTAGTACCCGGGCGGACGTATAATTGTAGTTGGACGTCAAGGTTTATGGCTGAG

CATAGTGGGGTATCTAA

>M01138:27:000000000-AHTAW:1:1101:23016:23221:dbj|AP012081.1| Margariscus margarita mitochondrial DNA almost complete genome except for D-loop

TAGAACAGGCTCCTCTAGGGAGG-TCTAAGGCACCGTCAGGTCCTTTGGGTTTCAAGCTA

ATGCTCGTAGTACCCGGGCGGACGTCTGTTTGTAGTTGGCCGTCTAGGTTTATGGCTGAG

CATAGTGGGGTATCTAA

>M01138:27:000000000-AHTAW:1:1101:5469:9522:dbj|AP012081.1| Margariscus margarita mitochondrial DNA almost complete genome except for D-loop

TAGAACAGGCTCCTCTAGGGGGG-TCTAAGGCACCGTCAGGTCCTTTGGGTTTCAAGCTA

CTGCTCGTAGTACCCGGGCGGACGTCTAATTGTAGTTGGACGTCTCGGTTTCTGGCTGAG

CATAGTGGGGTATCTAA

>M01138:27:000000000-AHTAW:1:1103:23407:5145:dbj|AP012081.1| Margariscus margarita mitochondrial DNA almost complete genome except for D-loop

TAGAACAGGCTCCTCTAGGGAGG-TCTAAGGCACCGTCAGGTCCTTTGGGTTTCAAGCTA

ATGCTCGTAGTACCCGGGCGGACGTCTAATTTTAGTTGGACGTCTAGTTTTATGGCTGAG

CATAGTGGGGTATCTAA

>M01138:27:000000000-AHTAW:1:1106:27020:17504:dbj|AP012081.1| Margariscus margarita mitochondrial DNA almost complete genome except for D-loop

TAGAACAGGCTCCTCTAGGGGGG-TCTAAGGCACCGTCAGGTCCTATGGGCTTCAAGCTA

ATGCTCGTAGTACCCGGGCGGACGTCTAATTGTAGTTGGCCGTCTAGGTTTATGGCTGAG

CATAGTGGGGTATCTAA

>M01138:27:000000000-AHTAW:1:1111:27698:11581:dbj|AP012081.1| Margariscus margarita mitochondrial DNA almost complete genome except for D-loop

TAGAACAGGCTCCTCTAGGGGGG-TTTAAGGCACCGTCAGGTCCTTTGGGTTTCAAGCTA

ATGCTCGTAGTTCCCGGGCGGACGTCTAATTGTAGTTGGACGTCCAGGTTTATGGCTGAG

CATAGTGGGGTATCTAA

>M01138:27:000000000-AHTAW:1:1112:26971:20199:dbj|AP012081.1| Margariscus margarita mitochondrial DNA almost complete genome except for D-loop

TAGAACAGGCTCCTCTAGGGGGG-TCTAAGGCACCGTCAGGTCCTTTGGGTTTCACGCTA

ATGCTCGGAGTACCCGGGCGGACGTCTAATTGTAGTTGGCCGTCTAGGTTTATGGCTGAG

CATAGTGGGGTATCTAA

>M01138:27:000000000-AHTAW:1:1116:10695:9332:dbj|AP012081.1| Margariscus margarita mitochondrial DNA almost complete genome except for D-loop

TAGAACAGGCTCCTCTAGGGAGG-TCTAAAGCACCGTCAGGTCCTTTGGGTTTCAAGCTA

ATACTCGTAGTACCCGGGCGGACGTCTAATTGTAGTTGGACGTCTAGGTTTATGGCTGAG

CATAGTGGGGTATCTAA

>M01138:27:000000000-AHTAW:1:1119:5860:6942:dbj|AP012081.1| Margariscus margarita mitochondrial DNA almost complete genome except for D-loop

TAGAACAGGCTCCTCTAGGGAGG-TCTAAGGCACCGTCAGGTCCTTTGAGTTTCAAGCTA

ATGCTCGTAGTACCCGGGCGGACGTCTAATTGTAGTTGGACGTCTAGGTTCATGGCTGAG

CATAGTGGGGTATCTAA

>M01138:27:000000000-AHTAW:1:2102:4994:17527:dbj|AP012081.1| Margariscus margarita mitochondrial DNA almost complete genome except for D-loop

TAGAACAGGCTCCTCTAGGGAGG-TCTAAGGCACCGTCAGGTCCTTTGGGTTTCAAGCTA

ATGCTCGTAGTACCCGGGCGGCCGTCTAATTGTAGTTGGACGTCTAGGTTTATGGATGAG

CATAGTGGGGTATCTAA

>M01138:27:000000000-AHTAW:1:1102:6873:10360:dbj|AP012081.1| Margariscus margarita mitochondrial DNA almost complete genome except for D-loop

TAGAACAGGCTCCTCTAGGGGGG-TCTAAGGCACCGTCGGGTCCTTTGGGCTTCAAGCTA

ATGCTCGTAGTACCCGGGCGGACGTCTGATTGTAGTTGGACGTCTAGGTTTATGGCTGAG

CATAGTGGGGTATCTAA

>M01138:27:000000000-AHTAW:1:1103:12780:22609:dbj|AP012081.1| Margariscus margarita mitochondrial DNA almost complete genome except for D-loop

TAGAACAGGCTCCTCTAGGGGGG-TCTAAGGCACCGTCAGATCCTTTGGGTTTCACGCTA

ATGCTCGTAGTACCCGGGCGGGCGTCTAATTGTAGTTGGACGTCTAGGTTTATGGCCGAG

CATAGTGGGGTATCTAA

>M01138:27:000000000-AHTAW:1:1103:9519:24826:dbj|AP012081.1| Margariscus margarita mitochondrial DNA almost complete genome except for D-loop

TAGAACAGGCTCCTCTAGGGAGG-TCTAAGGCACCGTCAGGTCCTTTGGGTTTCAAGCTA

ATGCTCGTAGTACCCGGGCGGACGTCTAATGTTAGTTGGACGGCTAGGTTTATGGCTGAG

CATAGTGGGGTATCTAA

>M01138:27:000000000-AHTAW:1:1105:26964:9342:dbj|AP012081.1| Margariscus margarita mitochondrial DNA almost complete genome except for D-loop

TAGAACAGGCTCCTCTAGGGGGG-TCTAAGGCACCGTCAGGTCCTTTGGGTTTCAAGCTA

ATGCTCGTAGTACCCGGGCGGACGTCTAATTGTAGTTAGCCGTCCAGGTTTATGGCTGAG

CATAGTGGGGTATCTAA

>M01138:27:000000000-AHTAW:1:1110:23215:4824:dbj|AP012081.1| Margariscus margarita mitochondrial DNA almost complete genome except for D-loop

TAGAACAGGCTCCTCTAGGGGGG-TTTAAGGCACCGTCAGGTCCTTTGGGCTTCAAGCTA

ATGCTCGTAGTACCCGGGCGGACGTCTAATTGTAGTTGGACGTCTCGGTTTATGGCTGAG

CATAGTGGGGTATCTAA

>M01138:27:000000000-AHTAW:1:1111:24556:4696:dbj|AP012081.1| Margariscus margarita mitochondrial DNA almost complete genome except for D-loop

TAGAACAGGCTCCTCTAGGGGGG-TCTAAGGCACCGTCAGGTCCTTTGGGTTTCACGCTA

ACGCTCGTAGTACCCGTGCGGACGTCTAATTGTAGTTGGCCGTCTAGGTTTATGGCTGAG

CATAGTGGGGTATCTAA

>M01138:27:000000000-AHTAW:1:1115:27623:11723:dbj|AP012081.1| Margariscus margarita mitochondrial DNA almost complete genome except for D-loop

TAGAACAGGCTCCTCTAGGGGGG-TCTAAGGCACCGTCAGGTCCTTTGGGTTTTAAGCTA

ATGCTCGTAGTATCCGGGCGGACGTCTAATTGTAGTTGGCCGTCTAGGTTTATGGCTGAG

CATAGTGGGGTATCTAA

>M01138:27:000000000-AHTAW:1:1119:2284:14464:dbj|AP012081.1| Margariscus margarita mitochondrial DNA almost complete genome except for D-loop

TAGAACAGGCTCCTCTAGGGGGG-TCTAAGGCACCGTCAGGTCCTTTGGGTTCCACGCTA

ATGCTCGTAGTACCCGGGCGGACGCCTAATTGTAGTTGGACGTCTAGGTTTATGGCTGAG

CATAGTGGGGTATCTAA

>M01138:27:000000000-AHTAW:1:2111:26774:8235:dbj|AP012081.1| Margariscus margarita mitochondrial DNA almost complete genome except for D-loop

TAGAACAGGCTCCTCTAGGGAGG-TCTAAGGCACCGTCAGGTCCTTTGGGTTTCAAGCTC

ATGCTCGTAGTACCCGGGCGGACGTCTAATTGTAGTTGGACGGCTAGGTTTATGGCTGAG

CATAGTGGGGTATCTAA

>M01138:27:000000000-AHTAW:1:1102:15812:3307:dbj|AP012081.1| Margariscus margarita mitochondrial DNA almost complete genome except for D-loop

TAGAACAGGCTCCTCTAGGGAGG-TCTAAGGCACCGTCAGGTCCTTTGGGTTTCAAGCTA

ATGCTCGTAGTACCAGGGCGGAAGTCTAATTGTAGTTGGCCGTCTAGGTTTATGGCTGAG

CATAGTGGGGTATCTAA

>M01138:27:000000000-AHTAW:1:1103:10013:1755:dbj|AP012081.1| Margariscus margarita mitochondrial DNA almost complete genome except for D-loop

TAGAACAGGCTCCTCTAGGGGGG-TTTAAGGCACCGGCAGGTCCTTTGGGTTTCAAGCTA

ATGCTCGTAGTACCCGGGCGGACGTCTAATTGTAGTTGGACGTCTAGGTCTATGGCTGAG

CATAGTGGGGTATCTAA

>M01138:27:000000000-AHTAW:1:1104:28192:18583:dbj|AP012081.1| Margariscus margarita mitochondrial DNA almost complete genome except for D-loop

TAGAACAGGCTCCTCTAGGGGGG-TCTAAGGCACCGTCAGGTCCTTTGGGTTTCACGCTA

ATGCTCGTTGTACCCGGGCGGACGTCTAATTGTAGTTGGACCTCTAGGTTTATGGCTGAG

CATAGTGGGGTATCTAA

>M01138:27:000000000-AHTAW:1:1107:22960:16412:dbj|AP012081.1| Margariscus margarita mitochondrial DNA almost complete genome except for D-loop

TAGAACAGGCTCCTCTAGGGGGG-TTTAAGGCACCGTCAGGTCCTTTGGGTTTCAAGCTA

ATGCTCGTAGTACCCGGGCGGACGTCTGATTGTTGTTGGACTTCTAGGTTTATGGCTGAG

CATAGTGGGGTATCTAA

>M01138:27:000000000-AHTAW:1:1112:28690:18398:dbj|AP012081.1| Margariscus margarita mitochondrial DNA almost complete genome except for D-loop

TAGAACAGGCTCCTCTAGGGAGG-TCTAAGGCACCGTCAGGTCCTTTGGGTTTCAAGCTA

ATGCTCGTAGTCCCCGGGCGGACGTCTAATTGTAGTTGGCCGTCTAGGTTTATGGCTGGG

CATAGTGGGGTATCTAA

>M01138:27:000000000-AHTAW:1:1114:18484:3288:dbj|AP012081.1| Margariscus margarita mitochondrial DNA almost complete genome except for D-loop

TAGAACAGGCTCCTCTAGGGGGG-TCTAAGGCCCCGCCAGGTCCTTTGGGCTTCAAGCTA

ATGCTCGTAGTACCCGGGCGGACGTCTAATTGTAGTTGGACGTCTAGGTTTATGGCTGAG

CATAGTGGGGTATCTAA

>M01138:27:000000000-AHTAW:1:1116:17759:8609:dbj|AP012081.1| Margariscus margarita mitochondrial DNA almost complete genome except for D-loop

TAGAACAGGCTCCTCTAGGGAGG-TCTAAGGCACCGTCAGGTCTTTTGGGTTTCAAGCTA

ATGCTCGTAGTACCCGGGCGGACGTCTAATTGTAGTCGGACGTCTAGGTTTATGGCTGAG

CATAGTGGGGTATCTAA

>M01138:27:000000000-AHTAW:1:2101:16669:11405:dbj|AP012081.1| Margariscus margarita mitochondrial DNA almost complete genome except for D-loop

TAGAACAGGCTCCTCTAGGGGGG-TCTAAGGCACCGTCAGGTCCTTTGGGTTTCCAGCTA

ATGCTCGTAGTACCCGGGCGGCAGTCTAATTGTAGTTGGACGTCTAGGTTTATGGCTGAG

CATAGTGGGGTATCTAA

>M01138:27:000000000-AHTAW:1:2116:4028:5657:dbj|AP012081.1| Margariscus margarita mitochondrial DNA almost complete genome except for D-loop

TAGAACAGGCTCCTCTAGGGAGG-TCTAAGGCACCGTCAGGTCCTTTGGGTTTCAAGCTA

ATGCTCGTAGTACCCGGGCGTACGTCTAATTGTAGTTGGACGTCTTGGTTTATGGCTGAG

CATAGTGGGGTATCTAA

>M01138:27:000000000-AHTAW:1:2117:17653:8026:dbj|AP012081.1| Margariscus margarita mitochondrial DNA almost complete genome except for D-loop

TAGAACAGGCTCCTCTAGGGGGG-TCTAAGGCACCGTCAGGTCCTTTGGGTTTCAAGCTA

ATGCTCGTAGTACGCGGGCGGACGTCTAATTGGAGTTGGACGTCTAGGTTTATGGCTGAG

CATAGTGGGGTATCTAA

>M01138:27:000000000-AHTAW:1:1101:29329:14090:dbj|AP012081.1| Margariscus margarita mitochondrial DNA almost complete genome except for D-loop

TAGAACAGGCTCCTCTAGGGGGG-TCTAAGGCACCGTCAGGTCCTTTGGGCTTGACGCTA

ATGCTCGTAGTCCCCGGGCGGACGTCTAATTGTAGTTGGACGTCTAGGTTTATGGCTGAG

CATAGTGGGGTATCTAA

>M01138:27:000000000-AHTAW:1:1102:26981:18626:dbj|AP012081.1| Margariscus margarita mitochondrial DNA almost complete genome except for D-loop

TAGAACAGGCTCCTCTAGGGGGG-TCTAAGGCACCGTCAGGTCCTTTGGGTTCCAAGCTA

ATGCTCGTAGTACCCGGGCGGACGTCTAATTGTAGTTGGCCGTCTAGGTCTATGGCTGAG

CATAGTGGGGTATCTAA

>M01138:27:000000000-AHTAW:1:1106:21177:1945:dbj|AP012081.1| Margariscus margarita mitochondrial DNA almost complete genome except for D-loop

TAGAACAGGCTCCTCTAGGGGGG-TCTAAGGCACCGTCAAGTCCTTTGGGTTTCAAGCTA

ATGCTCGTAGTACCCGGGCGGACGTCTAATGGTAGTTGGACGTCTAGGTTTAGGGCTGAG

CATAGTGGGGTATCTAA

>M01138:27:000000000-AHTAW:1:1106:24349:19155:dbj|AP012081.1| Margariscus margarita mitochondrial DNA almost complete genome except for D-loop

TAGAACAGGCTCCTCTAGGGGGG-TCTAAGGCACCGTCAGGTCCTTTGGGTTTCAAGCTA

ATGCTCGTCGTACCCGGGCGGACGCCTGATTGTAGTTGGACGTCTAGGTTTATGGCTGAG

CATAGTGGGGTATCTAA

>M01138:27:000000000-AHTAW:1:1106:3211:9220:dbj|AP012081.1| Margariscus margarita mitochondrial DNA almost complete genome except for D-loop

TAGAACAGGCTCCTCTAGGGGGG-TCTAAGGCACCGTCAGGTCCTTTGGGTTTCAAGCTA

ATGCTCCTAGTACCCGGGCGGACGTCTAATTGTAGTTGGACGGCTAGGTTTAGGGCTGAG

CATAGTGGGGTATCTAA

>M01138:27:000000000-AHTAW:1:1108:9671:5544:dbj|AP012081.1| Margariscus margarita mitochondrial DNA almost complete genome except for D-loop

TAGAACAGGCTCCTCTAGGGGGG-TCTAAGGCATCGTCAGGTCCTTTGGGTTTCAAGCTA

ATGCTCGTAGTACCCGGGCGGACGTCTGATTGTAGTTGGACGTCTAGGTTTATGGCTGGG

CATAGTGGGGTATCTAA

>M01138:27:000000000-AHTAW:1:1109:24053:7120:dbj|AP012081.1| Margariscus margarita mitochondrial DNA almost complete genome except for D-loop

TAGAACAGGCTCCTCTAGGAAGG-TCTAAGGCACCGTCAGGTCCTTTGGGTTTCACGCTA

ATGCTCGTAGTACCCGGGCGGACGTCGAATTGTAGTTGGACGTCTAGGTTTATGGCTGAG

CATAGTGGGGTATCTAA

>M01138:27:000000000-AHTAW:1:1110:26998:11100:dbj|AP012081.1| Margariscus margarita mitochondrial DNA almost complete genome except for D-loop

TAGAACAGGCTCCTCTAGGGAGG-TCTAAGGCACCGTCAGGTCCTTTGGGTTTCACGCTC

ATGCTCGTAGTACCCGGGCGGACGTCTAATTGTAGTTGGACGTCTCGGTTTATGGCTGAG

CATAGTGGGGTATCTAA

>M01138:27:000000000-AHTAW:1:1111:28630:11315:dbj|AP012081.1| Margariscus margarita mitochondrial DNA almost complete genome except for D-loop

TAGAACAGGCTCCTCTAGGGGGG-TCTAAGGCACCGTCAGGTCCTTTGGGTTTCACGCTA

ATGCTCGTAGTGCCCGGGCGGACGTCTAATTGTAGTTGGACGTGTAGGTTTATGGCTGAG

CATAGTGGGGTATCTAA

>M01138:27:000000000-AHTAW:1:1112:27569:10165:dbj|AP012081.1| Margariscus margarita mitochondrial DNA almost complete genome except for D-loop

TAGAACAGGCTCCTCTAGGGAGG-TCTAAGGCACCGTCAGGTCCTTTGGGTTTCAAGCTG

ATGCTCGTACTACCCGGGCGGACGTCTAATTGTAGTTGGACGTCTAGGTTTATGGCTGAG

CATAGTGGGGTATCTAA

>M01138:27:000000000-AHTAW:1:1113:21625:1180:dbj|AP012081.1| Margariscus margarita mitochondrial DNA almost complete genome except for D-loop

TAGAACAGGCTCCTCTAGGGGGG-TTTAAGGCACCGTCAGGTCCTTTGGGTTTTAAGCTA

ATGCTCGTAGTACCCGGGCGGACGTCTGATTGTAGTTGGCCTTCTAGGTTTATGGCTGAG

CATAGTGGGGTATCTAA

>M01138:27:000000000-AHTAW:1:1113:21824:2043:dbj|AP012081.1| Margariscus margarita mitochondrial DNA almost complete genome except for D-loop

TAGAACAGGCTCCTCTAGGGGGG-TCTAAGGCACCGTCAGGTCCTTTGGGCTTCAAGCTA

ATGCTCGTAGTACCCGGGCGGACGTCTAATTGTAGTTGGACGTCTAGGTTTGTGGCTGGG

CATAGTGGGGTATCTAA

>M01138:27:000000000-AHTAW:1:1114:28649:11512:dbj|AP012081.1| Margariscus margarita mitochondrial DNA almost complete genome except for D-loop

TAGAACAGGCTCCTCTAGGGGGG-TCTAAGGCACCGTCAGGTCCTTTGGGTTTCAAGCTA

ATGCTCGTAGTCCCCGGGCGGACGTATAATTGTAGTTGGCCGTCTAGGTTTATGGCTGAG

CATAGTGGGGTATCTAA

>M01138:27:000000000-AHTAW:1:1115:18452:23939:dbj|AP012081.1| Margariscus margarita mitochondrial DNA almost complete genome except for D-loop

TAGAACAGGCTCCTCTAGGGGGG-TCTAAGGCACCGTCAGGTCCTTTGGGTTCCAAGCTA

ATGCTCGTAGTACCCGGGCGGACGTCTAATTGTAGTTGGACGTCTAGGTTTATGGCGGGG

CATAGTGGGGTATCTAA

>M01138:27:000000000-AHTAW:1:1115:28473:13208:dbj|AP012081.1| Margariscus margarita mitochondrial DNA almost complete genome except for D-loop

TAGAACAGGCTCCTCTAGGGGGG-TCTAAGGCACCGTCAGGTCCGTTGGGTTTCAAGCTA

ATGCTCGTCGTACCCGGGCGGACGTCTAATTGTAGTTGGACGTCTAGGTTTATGGCTGTG

CATAGTGGGGTATCTAA

>M01138:27:000000000-AHTAW:1:1119:18473:6142:dbj|AP012081.1| Margariscus margarita mitochondrial DNA almost complete genome except for D-loop

TAGAACAGGCTCCTCTAGGGGGG-TCTAAGGCACCGTCAGGTCCTTTGGGTTTCAAGCTA

GTGCTCGTAGTACCCGGGCGGACGTCTAATGGTAGTTGGACGTCTAGGTTTATGGCTGGG

CATAGTGGGGTATCTAA

>M01138:27:000000000-AHTAW:1:2115:13527:2095:dbj|AP012081.1| Margariscus margarita mitochondrial DNA almost complete genome except for D-loop

TAGAACAGGCTCCTCTAGGGGGG-TCTAAGGCACCGTCAGGTCCTTTGGGTTTCAAGCTA

ATGCTCGTAGTACCCGGGCGGACGTCTAATTGTAGTTGGACGTCTAGGGGGATGGCTGAG

CATAGTGGGGTATCTAA

>M01138:27:000000000-AHTAW:1:2115:3978:10568:dbj|AP012081.1| Margariscus margarita mitochondrial DNA almost complete genome except for D-loop

TAGAACAGGCTCCTCTAGGGAGG-TCTAAGGCACCGTCAGGTCCTTTGGGTTTCAAGCTA

ATGCTCGTAGTACCCGGGCGTAAGTCTAAGTGTAGTTGGACGTCTAGGTTTATGGCTGAG

CATAGTGGGGTATCTAA

>M01138:27:000000000-AHTAW:1:1101:24671:6040:dbj|AP012081.1| Margariscus margarita mitochondrial DNA almost complete genome except for D-loop

TAGAACAGGCTCCTCTAGGGGGG-TCTAAGGCACCGTCAGGTCCTTTGGGTTTCAAGCTA

ATGCTCGTAGTACCCGGGCGGAAGTCACATTGTAGATGGACGTCTAGGTTTATGGCTGAG

CATAGTGGGGTATCTAA

>M01138:27:000000000-AHTAW:1:1102:10842:13066:dbj|AP012081.1| Margariscus margarita mitochondrial DNA almost complete genome except for D-loop

TAGAACAGGCTCCTCTAGGGGGG-TCTAAGGCACCGTCAGGTCCTTTGGGTTTCACGCCA

ATGCTCGTGGTACCCGGGCGGACGTCTAATTGTAGTTGGACGTCTAGGTTTATGGCTGAG

CATAGTGGGGTATCTAA

>M01138:27:000000000-AHTAW:1:1102:14256:3385:dbj|AP012081.1| Margariscus margarita mitochondrial DNA almost complete genome except for D-loop

TAGAACAGGCTCCTCTAGGGGGG-TCTAAGGCACCGTCAGGTCCTTTGGGTTTCAAGCTA

ATGCTCGGAGTACCCGGGAGGCCGTCTAATTGTAATTGGACGTCTAGGTTTATGGCTGAG

CATAGTGGGGTATCTAA

>M01138:27:000000000-AHTAW:1:1102:9131:23187:dbj|AP012081.1| Margariscus margarita mitochondrial DNA almost complete genome except for D-loop

TAGAACAGGCTCCTCTAGGGGGG-TCTAAGGCACCGGCAGGTCCGTTGGGTTTCAAGCTA

ATGCTCGTAGTACCCGGGCGGACGTCTAATGGTAGTTGGACGTCTAGGTTTATGGATGAG

CATAGTGGGGTATCTAA

>M01138:27:000000000-AHTAW:1:1103:14384:3626:dbj|AP012081.1| Margariscus margarita mitochondrial DNA almost complete genome except for D-loop

TAGAACAGGCTCCTCTAGGGGGG-TTTAAGGCACCGCCAGGTCCTTTGGGTTTCAAGCTA

ATGCTCGTAGTACCCGGGCGGACGTCTAATTGTAGTTGGACTTCTAGGTTTATGGCTGGG

CATAGTGGGGTATCTAA

>M01138:27:000000000-AHTAW:1:1103:2532:14491:dbj|AP012081.1| Margariscus margarita mitochondrial DNA almost complete genome except for D-loop

TAGAACAGGCTCCTCTAGGGGGG-TCTAAGGCACCTTCAGGTCCTTTGGGTTCCAAGCTA

ATGCTCGTAGTACCCGGGCGGACGTCTAATTGTAGTTGGGCGTCTAGGTTTATGGCTGAG

CATAGTGGGGTATCTAA

>M01138:27:000000000-AHTAW:1:1104:14444:18864:dbj|AP012081.1| Margariscus margarita mitochondrial DNA almost complete genome except for D-loop

TAGAACAGGCTCCTCTAGGGGGG-TCTAAGGCACCGTCAGTTCCTTTGGGTTTCAAGCTA

ATGCTCGTAGTACCCGGGCGGACGTCTGATTGTAGTTGGACGTCTAGGTTTATGGATGAG

CATAGTGGGGTATCTAA

>M01138:27:000000000-AHTAW:1:1104:18431:24444:dbj|AP012081.1| Margariscus margarita mitochondrial DNA almost complete genome except for D-loop

TAGAACAGGCTCCTCTAGGGGGG-TCTAAGGCACCGTCAGGTCCTTTGGGTTTCAAGCTA

ATGCTCGTAGTCCCCGGGCGGACGTCTAATGGTAGTTGGACGTCTAGTTTTATGGCTGAG

CATAGTGGGGTATCTAA

>M01138:27:000000000-AHTAW:1:1104:20029:4478:dbj|AP012081.1| Margariscus margarita mitochondrial DNA almost complete genome except for D-loop

TAGAACAGGCTCCTCTAGGGGGG-TTTAAGGCACCGTCAGGTCCTTTGGGTTTCAAGCTA

ATGCTCGTAGTCCCCGGGCGGACGTCTGATGGTAGTTGGACTTCTAGGTTTATGGCTGGG

CATAGTGGGGTATCTAA

>M01138:27:000000000-AHTAW:1:1105:14581:3979:dbj|AP012081.1| Margariscus margarita mitochondrial DNA almost complete genome except for D-loop

TAGAACAGGCTCCTCTAGGGAGG-TCTAAGGCACCGTCAGGTCCTCTGGGTTTCAAGCTA

ATGCTCGTAGTACCCGGGCGGACGTCTGATTGTAGTTGGCCGTCTAGGTTTATGGCTGGG

CATAGTGGGGTATCTAA

>M01138:27:000000000-AHTAW:1:1105:14777:1536:dbj|AP012081.1| Margariscus margarita mitochondrial DNA almost complete genome except for D-loop

TAGAACAGGCTCCTCTAGGGGGG-TCTAAGGCACCGTCAGGTCCTTTGGGTTTCAAGGTA

ATGCTCGGAGTACCCGGTCGGACGTCTAATTGTAGTTGGACGTCTAGGTTTATGGCTGAG

CATAGTGGGGTATCTAA

>M01138:27:000000000-AHTAW:1:1107:17833:12564:dbj|AP012081.1| Margariscus margarita mitochondrial DNA almost complete genome except for D-loop

TAGAACAGGCTCCTCTAGGGGGG-TCTAAGGCAACGTCAGGTCCTTTGGGTTTGACGCTA

ATGCTCGTAGTACCCGGGCGGACGTCTAATTGTAGTTGGACGTCTAGGTTTATGGCTGAG

CATAGTGGGGTATCTAA

>M01138:27:000000000-AHTAW:1:1107:22966:24462:dbj|AP012081.1| Margariscus margarita mitochondrial DNA almost complete genome except for D-loop

TAGAACAGGCTCCTCTAGGGAGG-TCTAAGGCACCGTCAGGTCCTTTTGGTTTCAAGCTA

ATGCTCGTAGTACCCTGTCGGACGTCTAATTGTAGTTGGACGTCTAGGTTTATGGCTGAG

CATAGTGGGGTATCTAA

>M01138:27:000000000-AHTAW:1:1108:7174:13479:dbj|AP012081.1| Margariscus margarita mitochondrial DNA almost complete genome except for D-loop

TAGAACAGGCTCCTCTAGGGGGG-TCTAAGGCACCGTCAGGTCCTTTGGGTTTCAAGCTA

ATGCTTGTAGTACCCGGGCGGACGTCTAATTGTTGTTGGACGTATAGGTTTATGGCTGGG

CATAGTGGGGTATCTAA

>M01138:27:000000000-AHTAW:1:1109:11328:24905:dbj|AP012081.1| Margariscus margarita mitochondrial DNA almost complete genome except for D-loop

TAGAACAGGCTCCTCTAGGGGGG-TCTAGGGCACCGTCAGGTCCCTTGGGTCTCAAGCTA

ATGCTCGTAGTACCCGGGCGGACGTCTAATTGTAGTTGGACGTCTAGGTTTATGGCTGAG

CATAGTGGGGTATCTAA

>M01138:27:000000000-AHTAW:1:1110:12629:2755:dbj|AP012081.1| Margariscus margarita mitochondrial DNA almost complete genome except for D-loop

TAGAACAGGCTCCTCTAGGGGGG-TCTAAGGCACCGTCAGGTCCTTTGGGTTTCACGCTA

ATGCTCGTAGTATCCGGGCGGACGTCGAATTGTAGTTGGACGTCTAGGTTTATGGCTGAG

CATAGTGGGGTATCTAA

>M01138:27:000000000-AHTAW:1:1110:16359:3634:dbj|AP012081.1| Margariscus margarita mitochondrial DNA almost complete genome except for D-loop

TAGAACAGGCTCCTCTAGGGGGG-TCTAAGGCACCGTCGGGTCCTTTGGGTTTCAAGCTA

ATGCTCGTAGTACCCGGGCGGACGTCTAATTGTAGTTGGACGTCTAGGTTCATGGCTAGG

CATAGTGGGGTATCTAA

>M01138:27:000000000-AHTAW:1:1110:28466:14410:dbj|AP012081.1| Margariscus margarita mitochondrial DNA almost complete genome except for D-loop

TAGAACAGGCTCCTCTAGGGGGG-TCTAAGGCACCGTCAGGTCCTTTGGGTTTCATGCTA

ATGCTCGTAGTACCCGGGCGGACGTCTAAGTGTAGTTGGCCGTCTAGGTTTATGGCTGAG

CATAGTGGGGTATCTAA

>M01138:27:000000000-AHTAW:1:1111:5836:14785:dbj|AP012081.1| Margariscus margarita mitochondrial DNA almost complete genome except for D-loop

TAGAACAGGCTCCTCTAGGGGGG-TCTAAGGCACCGTCAGGTCCTTTGGGTTTCAAGCTA

ATGCTCGTAGTACCCGGGCGGACGTCTAATTGTGGTTGGTCGTCTTGGTTTATGGCTGGG

CATAGTGGGGTATCTAA

>M01138:27:000000000-AHTAW:1:1111:9648:25035:dbj|AP012081.1| Margariscus margarita mitochondrial DNA almost complete genome except for D-loop

TAGAACAGGCTCCTCTAGGGGGG-TCTAAGGCACGGTCAGGTCCTTTGGGTTTCAAGCTA

ATGCTCGTAGTGCCCGGGCGGACGTCTAATCGTAGTTGGACGTCTAGGTTTATGGCTGAG

CATAGTGGGGTATCTAA

>M01138:27:000000000-AHTAW:1:1112:18540:6973:dbj|AP012081.1| Margariscus margarita mitochondrial DNA almost complete genome except for D-loop

TAGAACAGGCTCCTCTAGGGAGG-TCTAAGGCACTGTCAGGTCCTTTGGGTTTCAAGCTA

ATGCTCGTAGTACCCGGGCGGACGTCTAGTTGTAGTTGGACGTCCAGGTTTATGGCTGAG

CATAGTGGGGTATCTAA

>M01138:27:000000000-AHTAW:1:1113:22446:12619:dbj|AP012081.1| Margariscus margarita mitochondrial DNA almost complete genome except for D-loop

TAGAACAGGCTCCTCTAGGGAGG-TCTAAGGCACCGTCAGGTCCTTTGGGTTTCACGCCA

ATGCTCGTAGTACCCGGGCGGACGTCTAACTGTAGTTGGACGTCTAGGTTTATGGCTGAG

CATAGTGGGGTATCTAA

>M01138:27:000000000-AHTAW:1:1115:25762:9637:dbj|AP012081.1| Margariscus margarita mitochondrial DNA almost complete genome except for D-loop

TAGAACAGGCTCCTCTAGGGAGG-CCTAAGGCACCGTCAGGTCCTTTGGGTTTCAAGCTA

ATGCTCGTAGTACCCGGGCGGACGTCTAATTGTAGTTCGCCGTCTAGGTTTATGGCTGGG

CATAGTGGGGTATCTAA

>M01138:27:000000000-AHTAW:1:1115:28585:16373:dbj|AP012081.1| Margariscus margarita mitochondrial DNA almost complete genome except for D-loop

TAGAACAGGCTCCTCTAGGGAGG-TCTAAGGCACCGTCAGGTCCTTTTGGTTTCAAGCTA

ATGCTCGTAGTACCCGCGCGGACGTCTAATTGTAGTTGGACGACTAGGTTTATGGCTGAG

CATAGTGGGGTATCTAA

>M01138:27:000000000-AHTAW:1:1116:22322:1643:dbj|AP012081.1| Margariscus margarita mitochondrial DNA almost complete genome except for D-loop

TAGAACAGGCTCCTCTAGGGGGG-TCTAAGGCACCGTCAGGTCCTTTGGGTTTCAAGCTA

ATGCTCGTAGTCTCCGGGCGGACGTCTAATTGTAGTTGGTCGTCTAGGTTTATGGCTGAG

CATAGTGGGGTATCTAA

>M01138:27:000000000-AHTAW:1:1117:10383:22967:dbj|AP012081.1| Margariscus margarita mitochondrial DNA almost complete genome except for D-loop

TAGAACAGGCTCCTCTAGGGGGG-TCTAAGGCGCTGTCAGGTCCTTTGGGTTTCACGCTA

ATGCTCGTAGTACCCGGGCGGACGTCTAATTGTAGTTGGACGTCTAGGTTTATGGCTGAG

CATAGTGGGGTATCTAA

>M01138:27:000000000-AHTAW:1:1117:29406:13210:dbj|AP012081.1| Margariscus margarita mitochondrial DNA almost complete genome except for D-loop

TAGAACAGGCTCCTCTAGGGAGG-TCTAAGGCACCGTCAGGTCCTTTGGGTTTCACGCTA

ATGCTCGTAGTACCTTGGCGGACGTCTAATTGTAGTTGGCCGTCTAGGTTTATGGCTGAG

CATAGTGGGGTATCTAA

>M01138:27:000000000-AHTAW:1:2102:11225:12641:dbj|AP012081.1| Margariscus margarita mitochondrial DNA almost complete genome except for D-loop

TAGAACAGGCTCCTCTAGGGGGG-TTTAAGGCACCGTCAGGTCCTTTGGGTTCCAAGCTA

ATGCTCGTAGTACCAGGGCGGACGTCTAATTGTAGTTGGACGTCTAGGTTTATGGCTGAG

CATAGTGGGGTATCTAA

>M01138:27:000000000-AHTAW:1:2102:7619:10973:dbj|AP012081.1| Margariscus margarita mitochondrial DNA almost complete genome except for D-loop

TAGAACAGGCTCCTCTAGGGAGG-TCTAAAGCACCGTCAGGTCCTTTGGGTTTCAAGCTA

ATGCCCGTAGTACCCGGGCGGACGTCTAATTGTCGTTGGACGTCTAGGTTTATGGCTGAG

CATAGTGGGGTATCTAA

>M01138:27:000000000-AHTAW:1:2103:10195:1956:dbj|AP012081.1| Margariscus margarita mitochondrial DNA almost complete genome except for D-loop

TAGAACAGGCTCCTCTAGGGGGG-TCTAAGGCACCGTCTGGTCCTTTGGGTTTCAAGCTA

ATGCTCGTAGTACCCGGGCGAACGTCTAATTTTAGTTGGACGTCTAGGTTTATGGCTGAG

CATAGTGGGGTATCTAA

>M01138:27:000000000-AHTAW:1:2105:27632:7141:dbj|AP012081.1| Margariscus margarita mitochondrial DNA almost complete genome except for D-loop

TAGAACAGGCTCCTCTAGGTGGG-TTTAAGGCACCGTCAGGTCCTTTGGGTTTCAAGCTA

ATGCTCGTAGTCCCCGGGCGGACGTCTAATTGTAGTTGGACGTCTAGGTTTATGGCTGAG

CATAGTGGGGTATCTAA

>M01138:27:000000000-AHTAW:1:2106:23196:7319:dbj|AP012081.1| Margariscus margarita mitochondrial DNA almost complete genome except for D-loop

TAGAACAGGCTCCTCTAGGGAGG-TCTAAGGCACCGTCCGGTCCTTTGGGTTTCAAGCTA

ATGCTCGTAGTACCCGGTCGGACGTCTAATTGTAGTTGGACGTCTAGGTTTATGGCTGAG

CATAGTGGGGTATCTAA

>M01138:27:000000000-AHTAW:1:2107:10910:8480:dbj|AP012081.1| Margariscus margarita mitochondrial DNA almost complete genome except for D-loop

TAGAACAGGCTCCTCTAGGGGGG-TCTAAGGCACCGTCAGGCCCTTTGGGCTTCAAGCTA

AGGCTCGTAGTACCCGGGCGGACGTCTAATTGTAGTTGGACGTCTAGGTTTATGGCTGAG

CATAGTGGGGTATCTAA

>M01138:27:000000000-AHTAW:1:2107:16577:10224:dbj|AP012081.1| Margariscus margarita mitochondrial DNA almost complete genome except for D-loop

TAGAACAGGCTCCTCTAGGGGGG-TCTAAGGCACCGTCAGTCCCTTTGGGTTTCAAGCTA

ATGCTCGTAGTACCCGGGCGGACGTCTAATTGTAGTTGGACGTGTAGGTTTATGGCTGAG

CATAGTGGGGTATCTAA

>M01138:27:000000000-AHTAW:1:2107:17841:25187:dbj|AP012081.1| Margariscus margarita mitochondrial DNA almost complete genome except for D-loop

TAGAACAGGCTCCTCTAGGGAGG-TCTAAGGCACCGTCAGGTCCTTTGGGTTTCAAGCTA

ATGCTCGTAGTACCCGGGCGGACGTCTAATTGGCGGTGGACGTCTAGGTTTATGGCTGAG

CATAGTGGGGTATCTAA

>M01138:27:000000000-AHTAW:1:2109:11970:5279:dbj|AP012081.1| Margariscus margarita mitochondrial DNA almost complete genome except for D-loop

TAGAACAGGCTCCTCTAGGGAGG-TCTAAGGCACCGTCAGGTCCTTTGGGTTTCAAACTA

ATGCTCATAGTACCCGGGCGGATGTCTAATTGTAGTTGGACGTCTAGGTTTATGGCTGAG

CATAGTGGGGTATCTAA

>M01138:27:000000000-AHTAW:1:2109:9441:20303:dbj|AP012081.1| Margariscus margarita mitochondrial DNA almost complete genome except for D-loop

TAGAACAGGCTCCTCTAGGGAGG-TCTAAGGCACCGTCAGGTCCTTTGGGTTTCAAGCTA

ATGCTCGTAGTACCCGGGCGGATGTCTAATTGTTGTTGGCCGTCTAGGTTTATGGCTGAG

CATAGTGGGGTATCTAA

>M01138:27:000000000-AHTAW:1:2110:21920:14350:dbj|AP012081.1| Margariscus margarita mitochondrial DNA almost complete genome except for D-loop

TAGAACAGGCTCCTCTAGGGGGG-TCTAAGGCACCGTCAGGTCCTTTGGGTTTCAAGCTA

ATGCTCGTAGTAACCGGGCGGACGTATAATTGTAGTTGGACGTCTAGGTTTCTGGCTGAG

CATAGTGGGGTATCTAA

>M01138:27:000000000-AHTAW:1:2111:21482:10158:dbj|AP012081.1| Margariscus margarita mitochondrial DNA almost complete genome except for D-loop

TAGAACAGGCTCCTCTAGGGGGG-TCTAAGGCACCGGCAGGTCCGTTGGGTTTCAAGCTA

ATGCTCGTAGTACCCGGGTGGACGTCTAATTGTAGTTGGACGTCTCGGTTTATGGCTGAG

CATAGTGGGGTATCTAA

>M01138:27:000000000-AHTAW:1:2115:18904:23105:dbj|AP012081.1| Margariscus margarita mitochondrial DNA almost complete genome except for D-loop

TAGAACAGGCTCCTCTAGGGAGG-TCTAAGGCACCGTCAGGTCCTTTGGGTTTCCAGCTA

ATGCTCGTAGTACCCGGGCGGACATCTAATTGTAGTTGGACGTCTAGGTTTATGGCTGAG

CATAGTGGGGTATCTAA

>M01138:27:000000000-AHTAW:1:2116:16561:3415:dbj|AP012081.1| Margariscus margarita mitochondrial DNA almost complete genome except for D-loop

TAGAACAGGCTCCTCTAGGGGGG-TCTCAGGCACCGTCAGGTCCTTTGGGTTTCAAGCTA

ATGCGCGTAGTACCCGGGCGGACGTCTAATTGTAGTTGGACGTCTAGGTTTATGGCTGAG

CATAGTGGGGTATCTAA

>M01138:27:000000000-AHTAW:1:2117:19523:10151:dbj|AP012081.1| Margariscus margarita mitochondrial DNA almost complete genome except for D-loop

TAGAACAGGCTCCTCTAGGGGGG-TCTAAGGCACCGTCAGGTCCTCTGGGTTCCAAGCTA

ATGCTCGTAGTACCCGGGCGGACGTCTAATCGTAGTTGGACGTCTAGGTTTATGGCTGAG

CATAGTGGGGTATCTAA

>M01138:27:000000000-AHTAW:1:1115:8483:21958:dbj|AP012081.1| Margariscus margarita mitochondrial DNA almost complete genome except for D-loop

TAGAACAGGCTCCTCTAGGGGGG-TTTAAGGCACCGTCAGGTCCTTTGG-TTTCAAGCTA

ATGCTCGTAGTACCCGGGCGGACGTCTAATTGTAGTTGGACGTCTAGGTTTATGGCTGAG

CATAGTGGGGTATCTAA

>M01138:27:000000000-AHTAW:1:1101:10107:24641:dbj|AP012081.1| Margariscus margarita mitochondrial DNA almost complete genome except for D-loop

TAGAACAGGCTCCTCTAGGGGGG-TTTAAGGCACCGTCAGGTCCTTTGGGTTTCAAGCTA

-TGCTCGTAGTACCCGGGCGGACGTCTAATTGTAGTTGGACGTCTAGGTTTATGGCTGAG

CATAGTGGGGTATCTAA

>M01138:27:000000000-AHTAW:1:1105:16459:23975:dbj|AP012081.1| Margariscus margarita mitochondrial DNA almost complete genome except for D-loop

TAGAACAGGCTCCTCTAGGGGG--TCTAAGGCACCGTCAGGTCCTTTGGGTTTCAAGCTA

ATGCTCGTAATACCCGGGCGGACGTCTAATTGTAGTTGGACGTCTAGGTTTATGGCTGAG

CATAGTGGGGTATCTAA

>M01138:27:000000000-AHTAW:1:1101:18235:12339:dbj|AP012081.1| Margariscus margarita mitochondrial DNA almost complete genome except for D-loop

TAGAACAGGCTCCTCTAGGGGGG-TCTAAGGCACCGTCAGGTCCTTTGGGTTTCAAGCTA

ATGCTCGTGGTACCCGGGCGGACGTCTAATTGTAGTTGGACGTCTAGGTT-ATGGCTGAG

CATAGTGGGGTATCTAA

>M01138:27:000000000-AHTAW:1:1108:7087:13935:dbj|AP012081.1| Margariscus margarita mitochondrial DNA almost complete genome except for D-loop

TAGAACAGGCTCCTCTAGGGGGG-TCTA-GGCACCGTCAGGTCCTTTGGGTTTCAAGCTA

ATGCTCGTAGTACCCGGGCGGACGTCTAATTGCAGTTGGACGTCTAGGTTTATGGCTGAG

CATAGTGGGGTATCTAA

>M01138:27:000000000-AHTAW:1:1107:9407:10020:dbj|AP012081.1| Margariscus margarita mitochondrial DNA almost complete genome except for D-loop

TAGAACAGGCTCCTCTAGGGGG--CCTAAGGCACCGTCAGGTCCTTTGGGTTTCAAGCTA

GTGCTCGTAGTACCCGGGCGGACGTCTAATTGTAGTTGGACGTCTAGGTTTATGGCTGAG

CATAGTGGGGTATCTAA

>M01138:27:000000000-AHTAW:1:1101:20537:14790:dbj|AP012081.1| Margariscus margarita mitochondrial DNA almost complete genome except for D-loop

TAGAACAGGCTCCTCTAGGGAGG-TCTA-GGCACCGTCAGGTCCTTTGGGTTTCAAGCTA

ATGCTCGTAGTACCCGGGCGGACGTCCAATTGTAGTTGGACGTCTAGGTTTATGGCTGAG

CATAGTGGGGTATCTAA

>M01138:27:000000000-AHTAW:1:1107:21250:11146:dbj|AP012081.1| Margariscus margarita mitochondrial DNA almost complete genome except for D-loop

TAGAACAGGCTCCTCTAGGGGG--TCTAAGGCACCGTCAGGTCCTTTGGGTTTCAAGTTA

ATGCTCGTAGTACCCGGGCGGACGTCTAATTGTAGTTGGACGTATAGGTTTATGGCTGAG

CATAGTGGGGTATCTAA

>M01138:27:000000000-AHTAW:1:1114:18564:7233:dbj|AP012081.1| Margariscus margarita mitochondrial DNA almost complete genome except for D-loop

TAGAACAGGCTCCTCTAGGGGG--TCTAGGGCACCGCCAGGTCCTTTGGGTTTCAAGCTA

ATGCTCGTAGTACCCGGGCGGACGTCTAATTGTAGTTGGACGTCTAGGTTTATGGCTGAG

CATAGTGGGGTATCTAA

>M01138:27:000000000-AHTAW:1:2117:19844:22423:dbj|AP012081.1| Margariscus margarita mitochondrial DNA almost complete genome except for D-loop

TAGAACAGGCTCCTCTAGGGGG--TCTAAGGCACCGTCAGGTCCTTTGGGTTTCAAGCTA

ATGCTCGTAGTACCCGGGCGGTCGTCTAATTGTAGTTGGACGTCTAGGTTTATGGCTGCG

CATAGTGGGGTATCTAA

Part B. A phylogeny of the sequences from part A, constructed with Mega7 using the neighbor-joining algorithm, the Tamura 3-parameter mutational model, and assuming uniform rate variation among sites. A circular representation is used with branches collapsed below the 50% bootstrap support level (of 1000 replicates), with no significant clades found (complete polytomy). Note that Phoxinus eos is synonymous with Chrosomus eos.


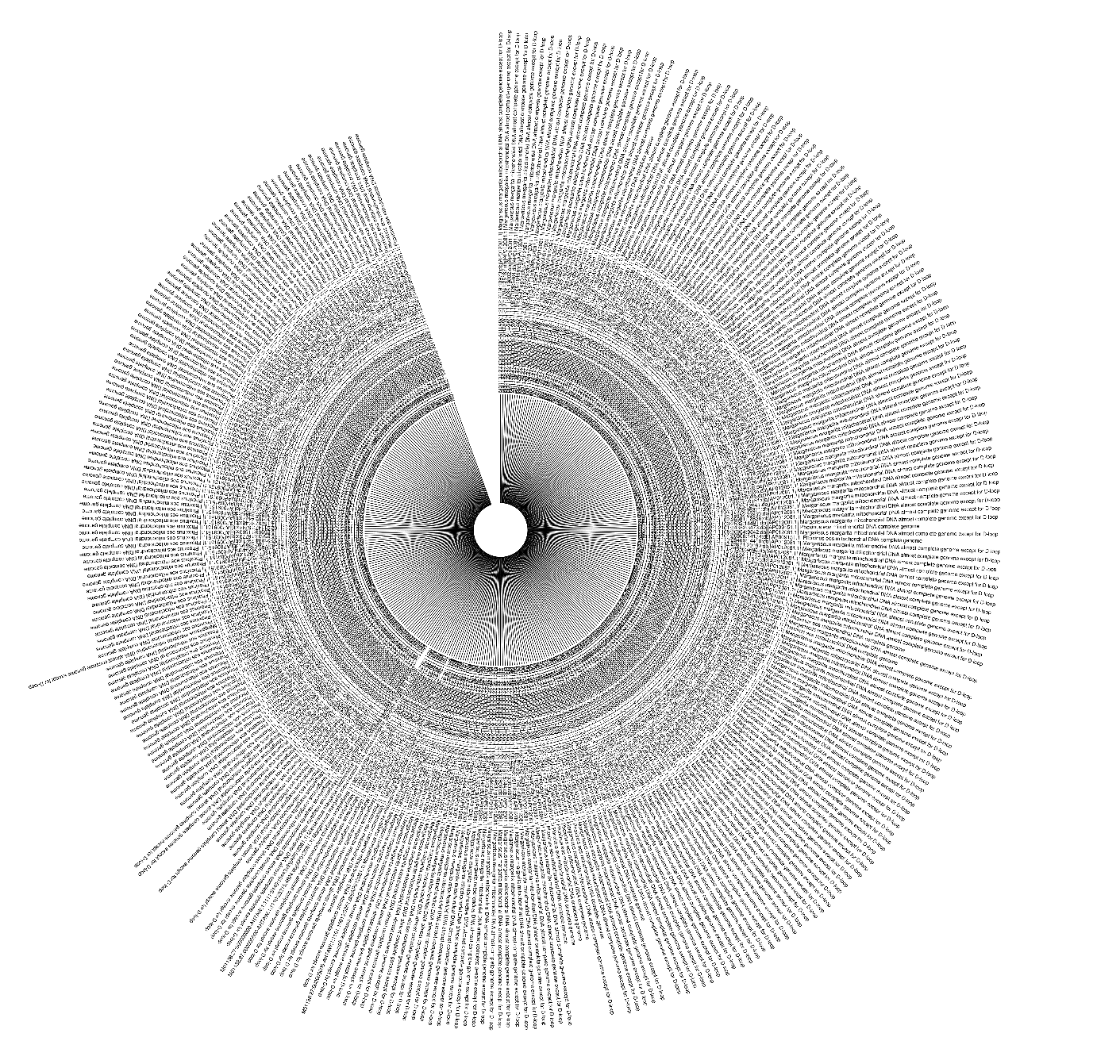

Supplement: Supplemental Information 2 [file peerj-06-5871-s002.docx]
