# Supplementary figures and images for: An experimental comparison of composite and grab sampling of stream water for metagenetic analysis of environmental DNA
Source: PeerJ. 2018 Dec 5;6:e5871. doi: 10.7717/peerj.5871 (PMC6286662; doi:10.7717/peerj.5871)

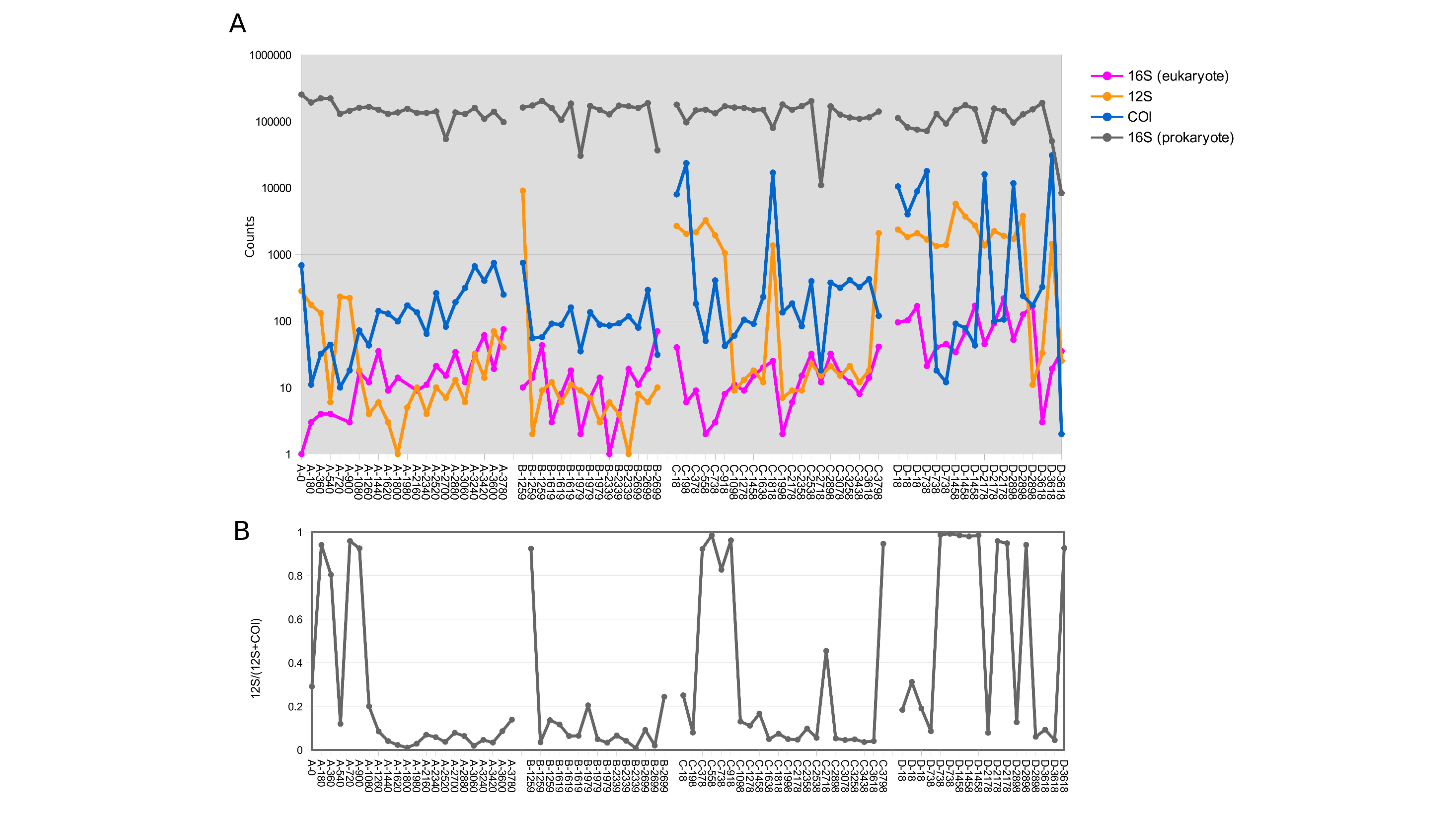

Supplement: Supplemental Information 9 — Counts by locus for each sample are shown in panel A and relative amounts of 12S and COI reads are shown in panel B. [file peerj-06-5871-s009.png]

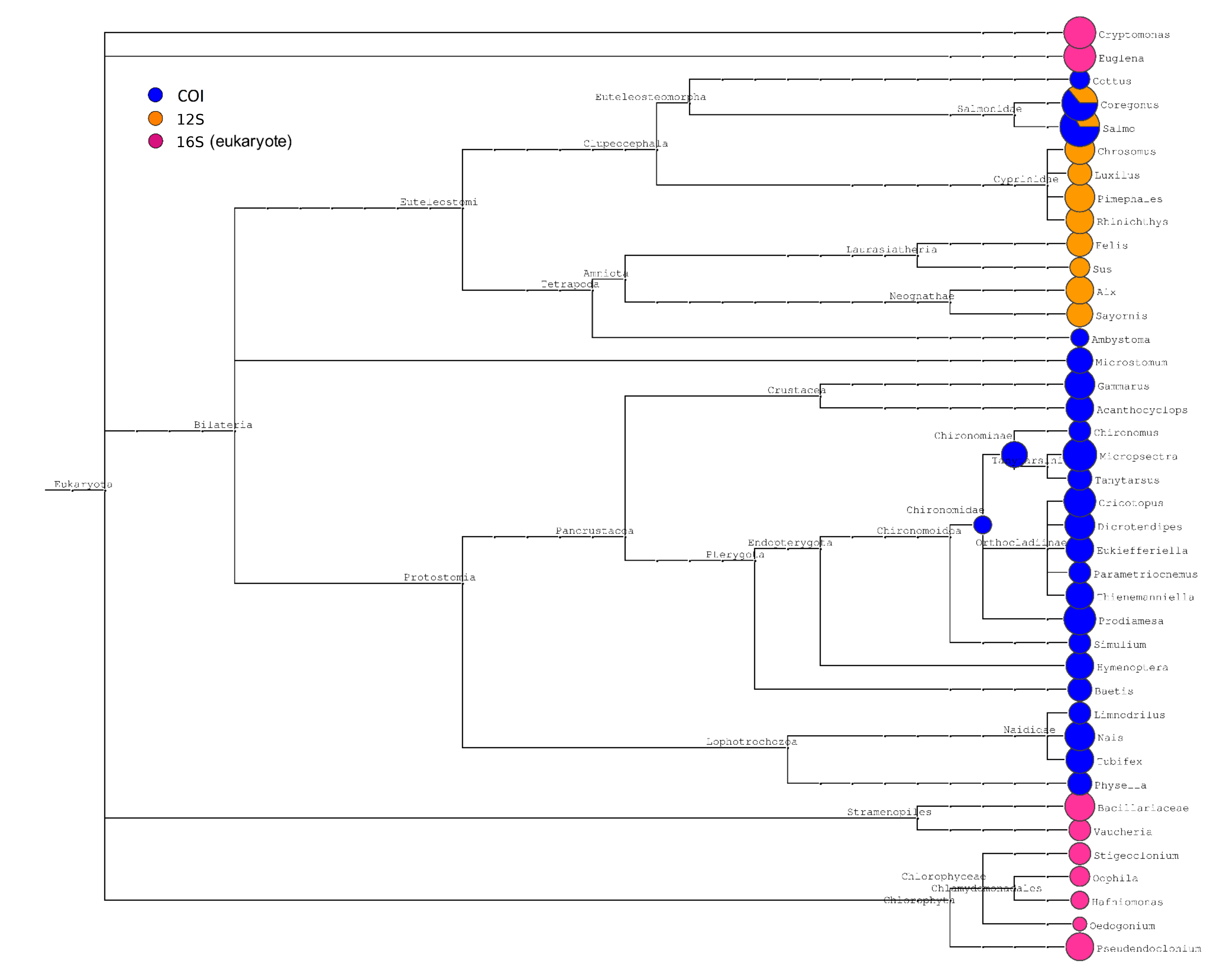

Supplement: Supplemental Information 10 — Size of circles is proportional to the log of sequence counts and colored according to the legned. The fish genera Salmo and Coregonus were recovered at both the 12S and cytochrome oxidase 1 loci. [file peerj-06-5871-s010.png]

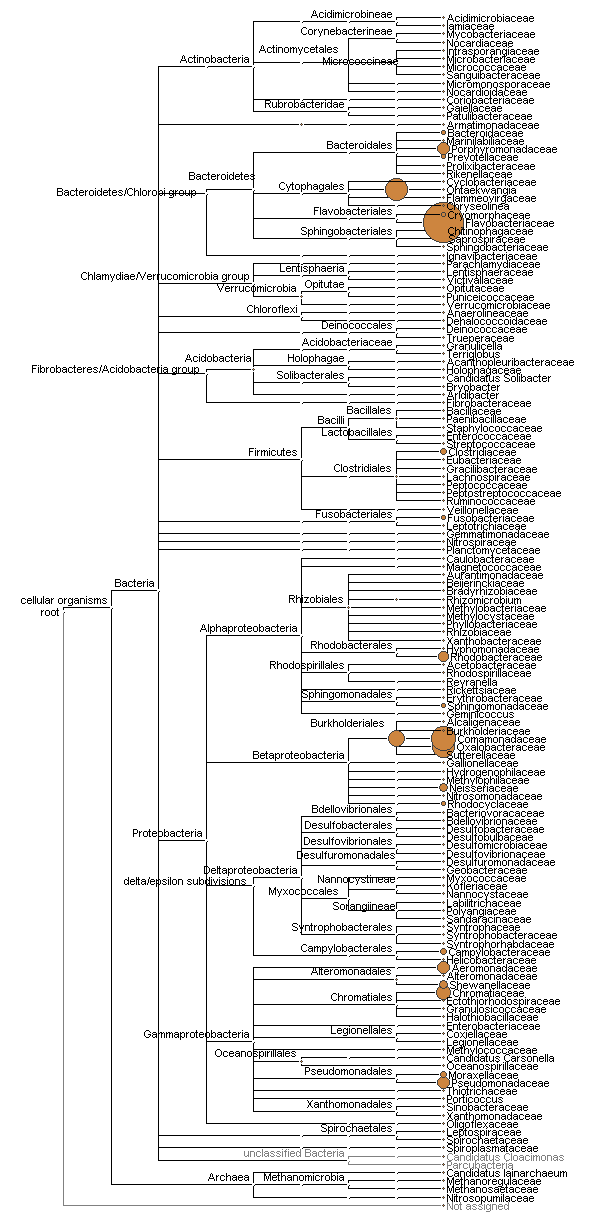

Supplement: Supplemental Information 11 — Size of circles is proportional to the square-root of sequence counts, in counts per million and summed across samples. [file peerj-06-5871-s011.png]

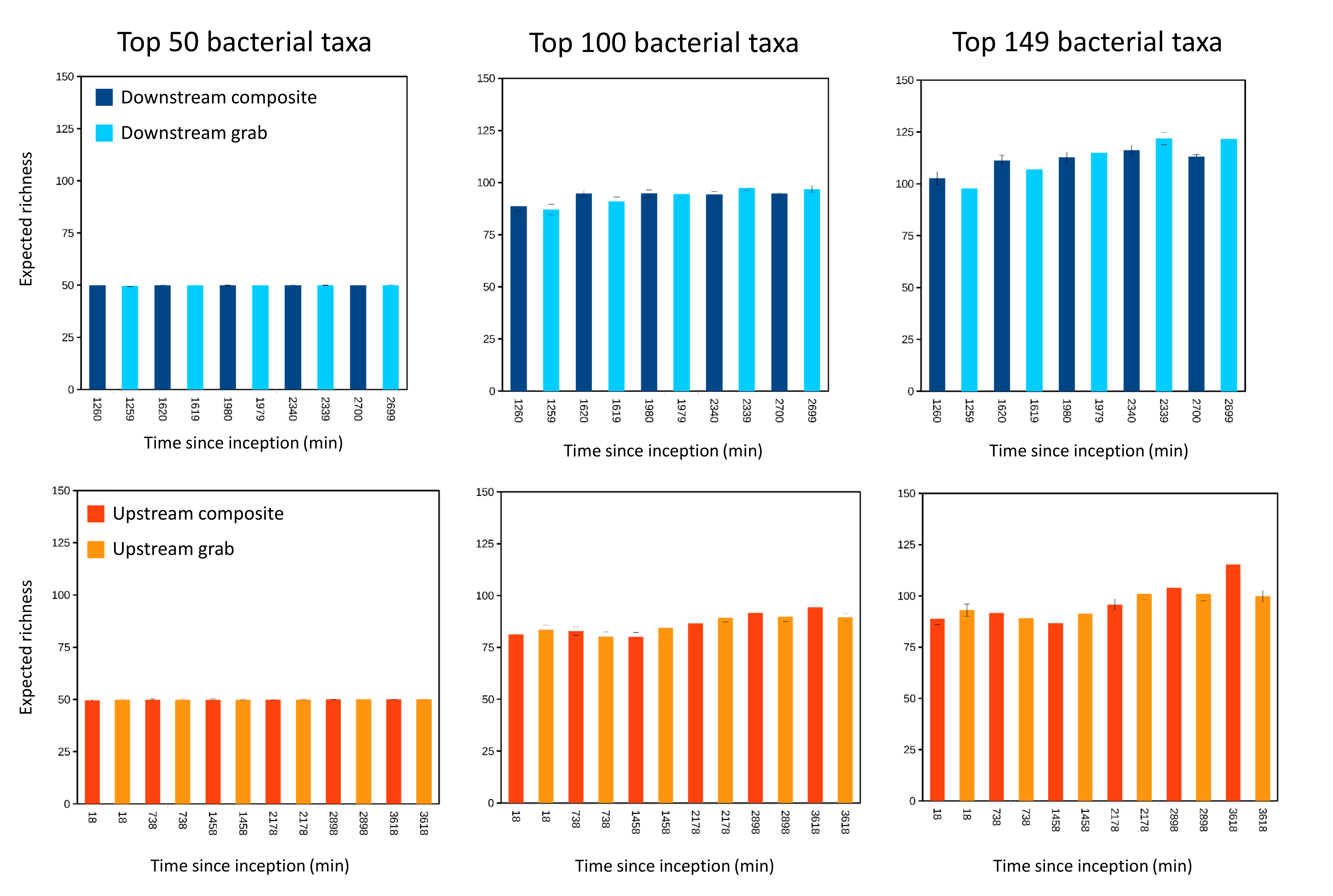

Supplement: Supplemental Information 12 — Richness of bacteria by sampler after stratification into three tiers of overall rank abundance. Rarefaction value is the minimum total count among the four samplers for each comparison. Upstream and downstream sites are shown separately (panels A–E). [file peerj-06-5871-s012.png]

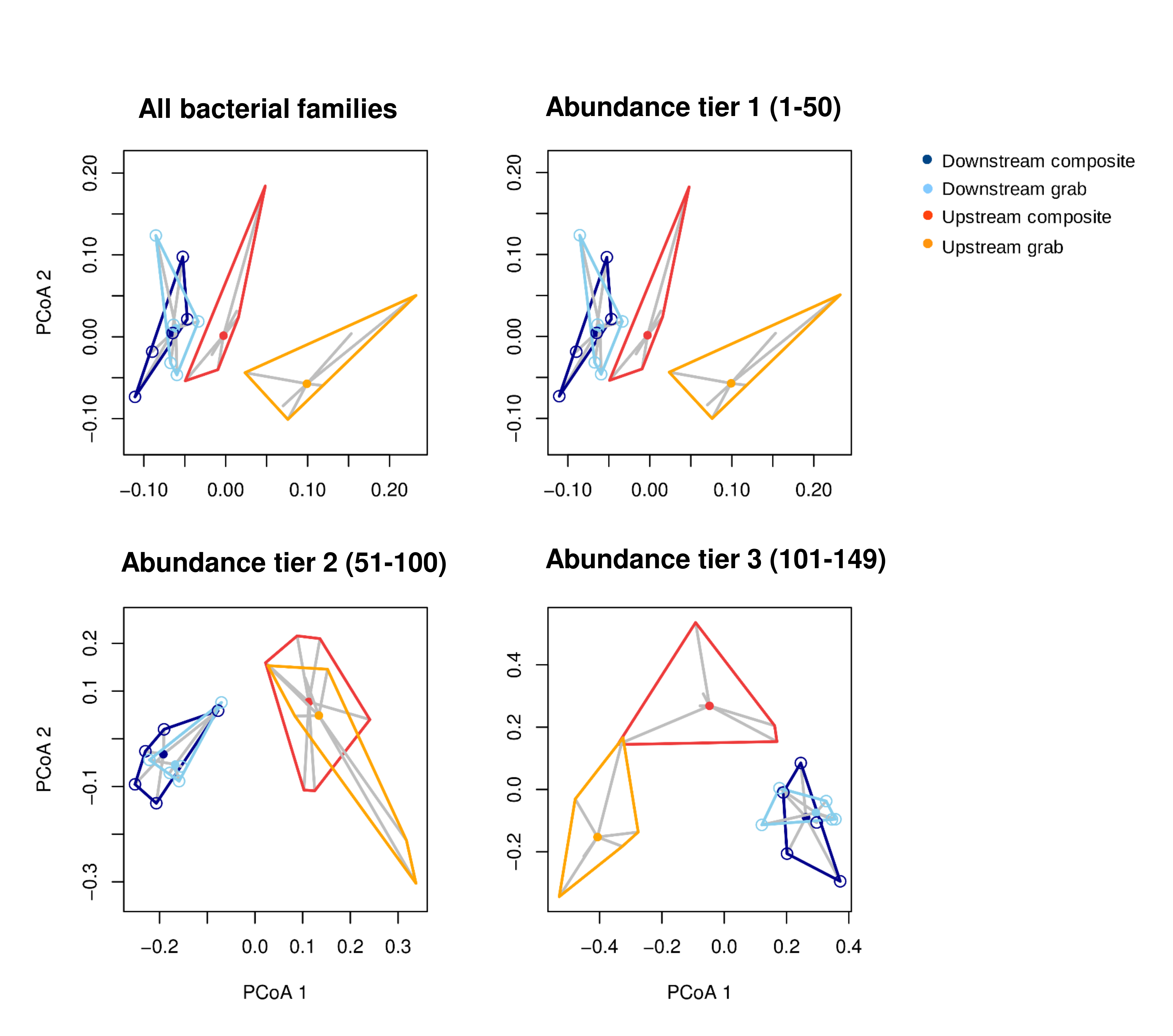

Supplement: Supplemental Information 13 — Dispersions are shown for each sampler as indicated by the legend, for all taxa as well as progressive tiers of relative abundance. [file peerj-06-5871-s013.png]

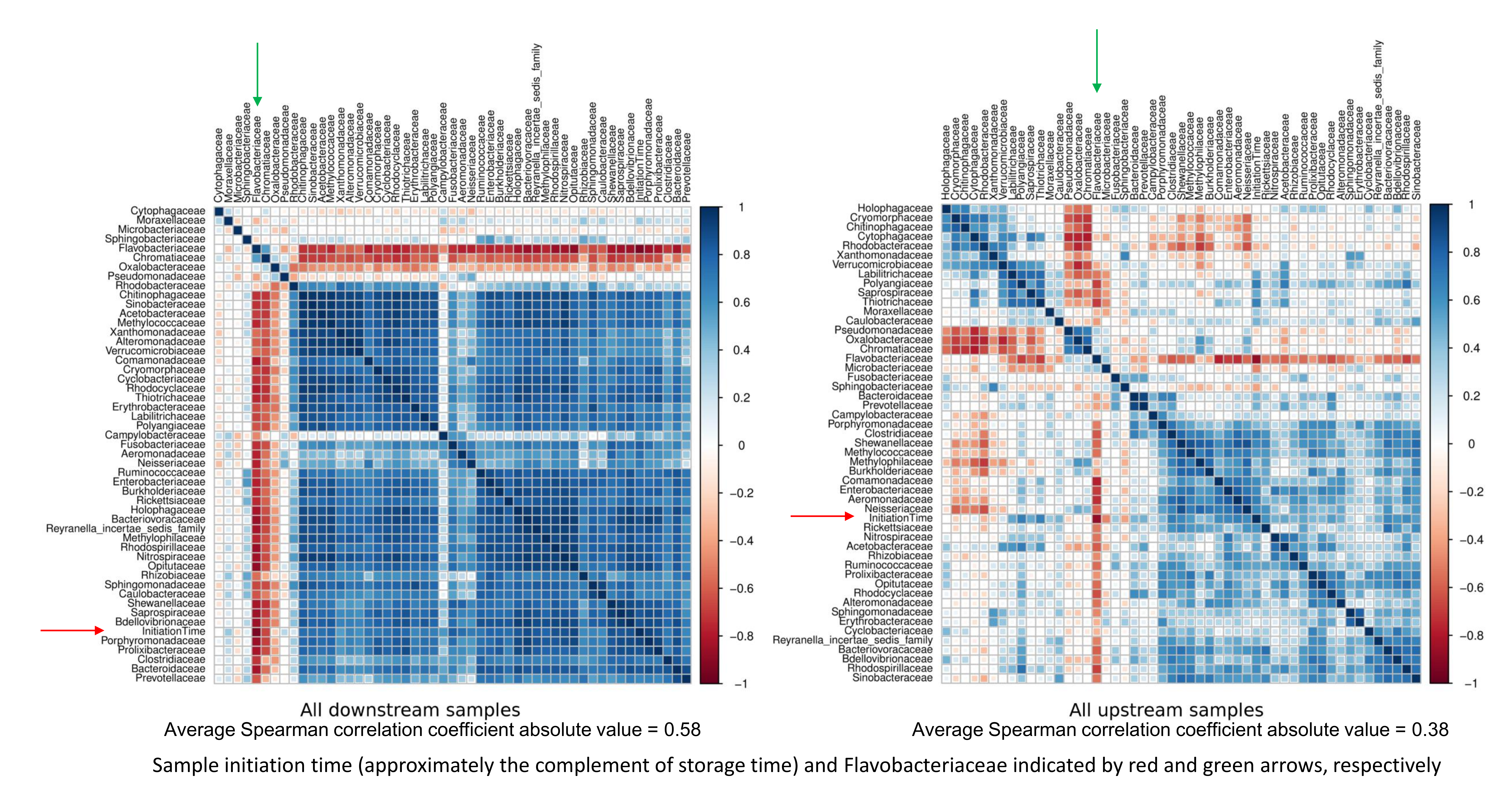

Supplement: Supplemental Information 14 — Matrices represent Spearman rank correlations between pairs of taxa, the strength and sign of which are indicated by the color scale and the size of the square in each matrix cell. [file peerj-06-5871-s014.png]
